# Exploration of commercial cyclen-based chelators for mercury-197 m/g incorporation into theranostic radiopharmaceuticals

**DOI:** 10.3389/fchem.2024.1292566

**Published:** 2024-02-08

**Authors:** Parmissa Randhawa, Imma Carbo-Bague, Patrick R. W. J. Davey, Shaohuang Chen, Helen Merkens, Carlos F. Uribe, Chengcheng Zhang, Marianna Tosato, François Bénard, Valery Radchenko, Caterina F. Ramogida

**Affiliations:** ^1^ Department of Chemistry, Simon Fraser University, Burnaby, BC, Canada; ^2^ Life Sciences Division, TRIUMF, Vancouver, BC, Canada; ^3^ Department of Molecular Oncology, BC Cancer, Vancouver, BC, Canada; ^4^ Department of Chemistry, University of British Columbia, Vancouver, BC, Canada

**Keywords:** radiopharmaceuticals, TCMC, mercury-197, Hg, radiometals, DOTAM, radiolabeling, radiopharmaceutical therapy

## Abstract

A comprehensive investigation of the Hg^2+^ coordination chemistry and ^197m/g^Hg radiolabeling capabilities of cyclen-based commercial chelators, namely, DOTA and DOTAM (aka TCMC), along with their bifunctional counterparts, *p*-SCN-Bn-DOTA and *p*-SCN-Bn-TCMC, was conducted to assess the suitability of these frameworks as bifunctional chelators for the ^197m/g^Hg^2+^ theranostic pair. Radiolabeling studies revealed that TCMC and DOTA exhibited low radiochemical yields (0%–6%), even when subjected to harsh conditions (80°C) and high ligand concentrations (10^–4^ M). In contrast, *p*-SCN-Bn-TCMC and *p*-SCN-Bn-DOTA demonstrated significantly higher ^197m/g^Hg radiochemical yields (100% ± 0.0% and 70.9% ± 1.1%, respectively) under the same conditions. The [^197 m/g^Hg]Hg-*p*-SCN-Bn-TCMC complex was kinetically inert when challenged against human serum and glutathione. To understand the differences in labeling between the commercial chelators and their bifunctional counterparts, non-radioactive ^nat^Hg^2+^ complexes were assessed using NMR spectroscopy and DFT calculations. The NMR spectra of Hg-TCMC and Hg-*p*-SCN-Bn-TCMC suggested binding of the Hg^2+^ ion through the cyclen backbone framework. DFT studies indicated that binding of the Hg^2+^ ion within the backbone forms a thermodynamically stable product. However, competition can form between isothiocyanate binding and binding through the macrocycle, which was experimentally observed. The isothiocyanate bound coordination product was dominant at the radiochemical scale as, in comparison, the macrocycle bound product was seen at the NMR scale, agreeing with the DFT result. Furthermore, a bioconjugate of TCMC (TCMC-PSMA) targeting prostate-specific membrane antigen was synthesized and radiolabeled, resulting in an apparent molar activity of 0.089 MBq/nmol. However, the complex demonstrated significant degradation over 24 h when exposed to human serum and glutathione. Subsequently, cell binding assays were conducted, revealing a *K_i_* value ranging from 19.0 to 19.6 nM. This research provides crucial insight into the effectiveness of current commercial chelators in the context of ^197m/g^Hg^2+^ radiolabeling. It underscores the necessity for the development of specific and customized chelators to these unique “soft” radiometals to advance ^197m/g^Hg^2+^ radiopharmaceuticals.

## Graphical Abstract

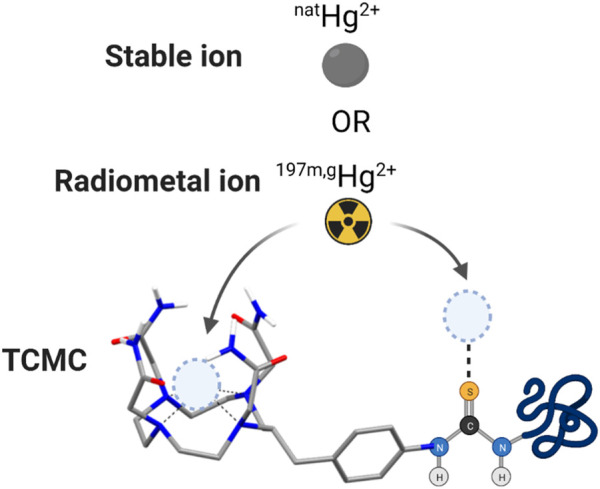

## 1 Introduction

With the expansion of radiopharmaceutical therapy (RPT) and diagnostic nuclear imaging of oncogenic diseases, the desire to add new elements to the current toolbox of medical isotopes has flourished within the last 20 years. Researchers have been actively seeking new medical isotopes with suitable physical decay properties for radiopharmaceutical applications, particularly those that can be used for *thera*peutic and diag*nostic* applications. These complementary isotopes form a so-called *theranostic* pair. One particular class of isotopes that has garnered significant attention recently is those emitting Meitner-Auger electrons (MAEs)^1^, which are useful for RPT (Ku et al., 2019). MAEs emit Auger electrons and conversion electrons (CEs), both are types of particulate radiation of high potency with short-effective ranges resulting in target-specific therapy with a reduction in side effects to healthy tissues, similar to alpha emitters. Additionally, many MAE emitters are readily accessible via production on small medical cyclotrons (Radchenko and Hoehr, 2020; Filosofov, Kurakina, and Radchenko, 2021) and neutron activation (Kim and Adams, 1967).

Among the MAE emitter candidates, isomers mercury-197 m and mercury-197 g (^197 m/g^Hg) have received interest over the past years due to the high number of MAEs released per decay and their ability to form a true radio-theranostic pair (Randhawa et al., 2021a). This same-element theranostic pair utilizes the gamma-ray emission (*E*γ = 133.98 keV, *I*γ = 34.8%) of ^197 m^Hg (*t*_1/2_ = 23.8 h, isomeric transition (IT) (91%)) for single-photon emission computed tomography (SPECT) imaging and the conversion electron (average yield/decay = 2.4) and AE (average yield/decay = 42.6) emissions of both ^197 m^Hg and ^197g^Hg (*t*_1/2_ = 64.1 h, electron capture (EC) (100%)) for therapy (Randhawa et al., 2021a). The successful utilization of this same-element theranostic pair carries enormous potential, as it offers the possibility of utilizing the same drug architecture for both imaging and therapeutic applications, potentially allowing for increased accuracy in monitoring of the radiopharmaceutical’s distribution and therapeutic efficacy.

However, the use of ^197 m/g^Hg in radiopharmaceutical design is currently limited by a lack of knowledge surrounding how to incorporate this element efficiently and securely into a drug construct. Metal-based radiopharmaceuticals commonly consist of four parts: the radioisotope; the bifunctional chelate (BFC); a linker; and a targeting vector (e.g., antibody or peptide). The BFC is a critical component as it must stably bind the radiometal affixing it to the biomolecule targeting vector, ensuring the radiation payload is successfully delivered to the target site. Therefore, exploiting the clinical capability of novel radiometals heavily depends on identifying efficient BFCs for each radiometal.

**FIGURE 1:**
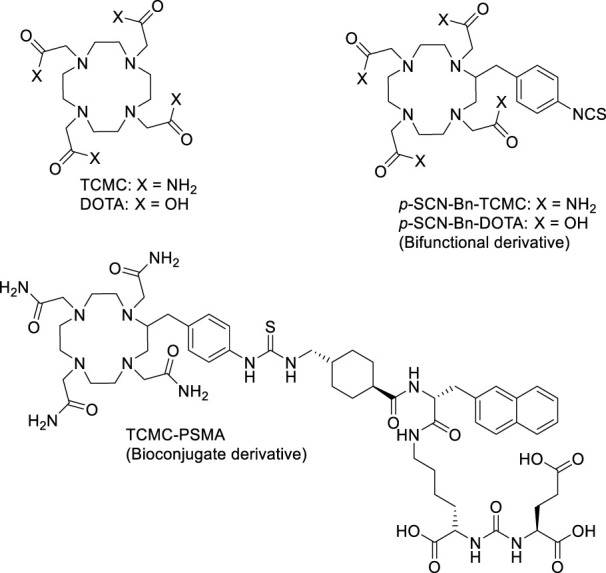
Chemical structures of the ligands DOTA, TCMC, *p*-SCN-Bn-DOTA, *p*-SCN-Bn-TCMC, and bioconjugate TCMC-PSMA discussed and studied with ^197m/g^Hg^2+^ herein.

The most prominent chelating ligands exploited in radiopharmaceutical design tend to be commercially available, given their ease of access and convenience for use. The ubiquitous commercial standard, 1,4,7,10-tetraazacyclododecane-1,4,7,10-tetraacetic acid (DOTA) (Figure 1), forms kinetically inert and thermodynamically stable complexes with a variety of trivalent and divalent radiometal ions, and is used prominently for complexation of radiometals such as ^225^Ac^3+^, ^44^Sc^3+^, ^111^In^3+^, ^177^Lu^3+^, ^64^Cu^2+^, ^203^Pb^2+^ among others (Hanaoka et al., 2009; Paganelli et al., 2014; Price and Chris, 2014; Zhang et al., 2017; Kent et al., 2019; Ramogida et al., 2019; Stenberg et al., 2020; McNeil et al., 2021; Yang et al., 2021).

Although the non-radioactive Hg^2+^-DOTA complex has been reported in the literature (Kodama, 1994), in our previous work, the ^197m/g^Hg^2+^ radiolabeling of DOTA was found to be unsuccessful—an expected result given the metal’s preferred coordination to soft Lewis bases according to the Hard-Soft-Acid-Base (HSAB) theory (Randhawa et al., 2021b; Randhawa et al. 2022; Randhawa et al. 2023). However, a promising alternative lies in the chelator 1,4,7,10-tetrakis-(carbamoylmethyl)-1,4,7,10-tetraazacyclododecane (TCMC or DOTAM) (Figure 1). TCMC exchanges the “harder” carboxylic acids of DOTA for “softer” amide donor arms. TCMC is used almost exclusively for the complexation of lead radioisotopes (Chappell et al., 2000; McNeil et al., 2021)—an intermediate metal ion according to HSAB theory (Chappell et al., 2000). As a well-established chelate, the bifunctional isothiocyanate-based derivative of TCMC, *S*-2-(4-isothiocyanatobenzyl)-1,4,7,10-tetraaza-1,4,7,10-tetra(2-carbamoylmethyl)cyclododecane (*p*-SCN-Bn-TCMC), is also commercially available (Figure 1). Notably, TCMC complexes with ^212^Pb^2+^ at exceptionally low chelator concentrations [85 kBq/µmol] (McNeil et al., 2023), forming a highly kinetically inert complex, escalating its use in clinical trials (Meredith R. F. et al., 2014; Meredith R. et al., 2014; Delpassand et al., 2022; dos Santos et al., 2019).

The ^nat^Hg^2+^ complex of TCMC has been previously reported (Hancock, Reibenspies, and Maumela, 2004), suggesting a 6-coordinate binding site based on its crystal structure, with all four nitrogen atoms in the macrocyclic backbone and two oxygen atoms from adjacent pendant amide groups coordinating the metal. Therefore, TCMC has the potential to have an increased affinity to ^197m/g^Hg^2+^ compared to DOTA based on its softer nature.

In this study, a comparative investigation of commercial chelators [TCMC, *p*-SCN-Bn-TCMC and S-2-(4-isothiocyanatobenzyl)-1,4,7,10-tetraazacyclododecane tetraacetic acid (*p*-SCN-Bn-DOTA)] (Figure 1) with ^197m/g^Hg^2+^ have been conducted for the first time. Given the limited literature on ^197m/g^Hg labeling with chelators, elucidation of the structure, bonding and radiolabeling behaviours of ^nat^Hg/^197m/g^Hg^2+^ with commercially available chelators is fundamental knowledge needed to gain a better understanding of the coordination behavior of radiomercury. Although the identification of better-suited ^197m/g^Hg^2+^ chelators such as N-benzyl-2-(1,4,7,10-tetrathia-13-azacyclopentadecan-13-yl)acetamide (NS_4_-BA) have been most recently reported by our group (Randhawa et al., 2023), the studies presented herein will provide necessary information to guide the future design of chelators for this isomer pair, crucial for its future use in clinical and preclinical applications.

With the objective above, the coordination chemistry of both the TCMC and *p*-SCN-Bn-TCMC with Hg^2+^ were studied using nuclear magnetic resonance (NMR) spectroscopy accompanied by density functional theory (DFT) calculations to aid in structure elucidation and bonding. Following ^197 m/g^Hg radiolabeling, the *in vitro* stabilities of [^197m/g^Hg][Hg(TCMC)]^2+^ and [^197m/g^Hg][Hg (*p*-SCN-Bn-TCMC)]^2+^ were investigated. Further, a model TCMC bioconjugate was synthesized by conjugation of *p*-SCN-Bn-TCMC to the prostate-specific membrane antigen (PSMA)-targeting peptide, (((S)-5-((R)-2-((1R,4R)-4-(aminomethyl)cyclohexane-1-carboxamido)-3-(naphthalen-2-yl)propanamido)-1-carboxypentyl)carbamoyl)-L-glutamic acid (TCMC-PSMA, Figure 1). The ^197 m/g^Hg radiolabeling and the *in vitro* stability of the bioconjugate were assessed to determine the effect of bifunctionalization on ^197 m/g^Hg incorporation. Cell binding studies were undertaken to ascertain the effect of labeled bioconjugation affinity to bind the surface receptor PSMA moiety. These studies provide valuable insight into the potential application of TCMC and its derivatives as chelators for targeted radiopharmaceuticals using ^197m/g^Hg, and emphasizes the crucial factors to consider when working with ^197m/g^Hg and evaluating the effectiveness of chelating ligands in this context.

## 2 Experimental

### 2.1 Materials and methods

All solvents and reagents were purchased from commercial suppliers and used as received unless otherwise noted. Ultrapure concentrated hydrochloric acid (HCl, 99.99% trace metal grade, 37%), sodium hydroxide (NaOH, ACS reagent, ≥97%, pellets), sodium deuteroxide (NaOD, 30 wt. % in D_2_O), deuterium chloride (DCl, 35 wt. % in D_2_O), dimethyl sulfoxide (DMSO), DOTA, and human serum were purchased from Sigma Aldrich (St. Louis, MO). TCMC and the bifunctional chelators *p*-SCN-Bn-TCMC and *p-*SCN-Bn-DOTA were purchased from Macrocyclics Inc. (Plano, TX, United States). Millipore system (Direct-Q^®^ 3UV with Pump, 18 MΩ cm^−1^) provided ultrapure water. Deuterated solvents used for NMR analysis were purchased from Sigma or Cambridge Isotope Laboratories Inc. and exhibited an isotopic purity between 99.8% and 99.9%. Solvents noted as “dry” were obtained following storage over 3 Å molecular sieves under an argon environment. All NMR spectra were recorded on Bruker AVANCE III 600 MHz with a QCI cryoprobe, Bruker AVANCE III 500 MHz, or Bruker AVANCE III 400 MHz instruments. Chemical shifts are reported in parts per million (ppm) and are referred to the residual solvent peak. Multiplicity is reported as follows: s = singlet, t = triplet, m = multiplet, and br = broad peak. Coupling constants (*J*) are reported in hertz (Hz). High-resolution electrospray-ionization mass spectrometry (ESI-HRMS) was performed on an Agilent 6210 time-of-flight (TOF) instrument using a Halo LC-MS column (5 µm, C18, 90 Å, 2.1 × 50 mm) (solvent system—A: 90% H_2_O, 10% MeCN, 6 mM NH_4_OAc; B: 10% H_2_O, 90% MeCN, 6 mM NH_4_OAc; gradient: 0% B (0–0.5 min), 0%–100% B (0.5–6.0 min), 100% B (6.0–6.7 min), 100%–0% B (6.7–6.8 min), 0% B (6.8–8 min); flow rate: 0.4 mL/min. An Agilent Technologies 1,100 high-pressure liquid chromatography (HPLC) system equipped with a quaternary pump, UV detector, Kinetex LCMS column (5 µm, C18, 100 Å, 150 × 100 mm), and fraction collector was used for the analysis and purification of TCMC-PSMA. Fourier-transform infrared spectroscopy (FTIR) data were obtained using a PerkinElmer FT-IR spectrometer. Ultraviolet-visible (UV-visible) spectra were recorded using a Perkin Elmer UV-Vis lambda 850 spectrometer interfaced to UV-WinLab. A quartz cuvette (1 mL) was used for all experiments. The radiolabeling of ligands was monitored using silica-impregnated instant thin-layer chromatography paper (iTLC-SG, Agilent Technologies, Santa Clara, CA, United States). Data were analyzed on an Eckert & Ziegler AR-2000 TLC scanner and processed with Eckert & Ziegler WinScan software (Hopkinton, United States). A Capintec CRC-55tR dose calibrator well counter set at the ^197g^Hg calibration (calibration number: 197) Capintec activity readings were cross-referenced with the gamma spectrometer value to ensure accuracy in the reading values. The Capintec was used to measure the activity before radiolabeling reactions. Radio-HPLC was carried out using an Agilent 1,200 instrument equipped with a Halo column (5 μm, C18, 90 Å, 50 mm × 3 mm) Waters Alliance HT 2795 separation module equipped with a Raytest Gabi Star NaI (Tl) detector, and a Waters 996 photodiode array (PDA) detector. Bio-rad Mini-PROTEAN Tetra Vertical Electrophoresis Cell instrument was used for all sodium dodecyl sulphate-polyacrylamide gel electrophoresis (SDS-PAGE) measurements with 4%–20% Mini-PROTEAN^®^ TGX™ precast protein gels. The SDS-PAGE gel electrophoresis reagents, including the MW standards, TGS buffer, Laemmli sample buffer, and Bio-Safe^TM^ Coomassie stain, were all also purchased from Bio-rad.

Caution!!! ^197m/g^Hg^2+^ produces ionizing radiation and should be handled in laboratories approved for radioactive work using safe lab practices.

Caution!!! Mercury is a toxic heavy metal, and its compounds should be treated accordingly.

### 2.2 Synthesis of TCMC-PSMA

PSMA was synthesized according to published procedures (H. T. Kuo et al., 2018a). PSMA was purified by semi-preparative HPLC prior to conjugation (solvent system—A: 0.1% trifluoroacetic acid (TFA) in deionized water, B: 0.1% TFA in MeCN; gradient: 5%–80% B over 20 min; flow rate: 3.0 mL/min; UV detection at 220 nm). Following a similar procedure to a purified solution of PSMA (0.8 mg, 0.0012 mmol, 1.0 equiv.) in dry DMF (0.2 mL) was added to a solution of *p*-SCN-Bn-TCMC (3.2 mg, 0.0046 mmol, 3.8 equiv.) and *N*,*N*-diisopropylethylamine (6.3 μL, 0.035 mmol, 29 equiv.) in dry DMF (0.3 mL) (Thiele et al., 2017). The solution was left to react at ambient temperature overnight. The reaction was subsequently dried under air flow and washed with diethyl ether. The crude product was dissolved in 1:1 MeCN/H_2_O and purified using the HPLC method described above: *p*-SCN-Bn-TCMC (*t*_R_ = 9.3 min), PSMA (*t*_R_ = 9.8 min), TCMC-PSMA (*t*_R_ = 10.5 min). Product fractions were collected, lyophilized, and obtained as a TFA salt (49% yield). ESI-HRMS *m*/*z* calcd. for [C_57_H_82_N_14_O_13_S+H]^+^ 1,203.598; found 1,203.575 [M + H]^+^, LC-HRMS *t*_R_ = 5.034 min.

### 2.3 Non-radioactive Hg^2+^ complexation for NMR and MS experiments

#### 2.3.1 [Hg(TCMC)]^2+^

Mercury dichloride (HgCl_2_) in deuterated water (D_2_O, 0.03 M; 0.25 mL) was added to a solution of TCMC in D_2_O (0.027 M, adjusted to pD 6 using NaOD; 0.25 mL) giving a ligand/Hg^2+^ ratio of approx. 1:1.1, and the pD of the solution was adjusted to 5.4 and 7.4 using 1 M NaOD or 1 M DCl. The solution was heated for 1 h at 80°C. For NOESY experiments, a higher concentration of 10^–2^ M metal complex in solution was used which was not soluble in D_2_O. As a result, DMSO-*d*_*6*_ was used as an NMR solvent to increase the solubility of the complex instead of D_2_O. These solutions were used for NMR and MS experiments. ESI-HRMS *m*/*z* calcd. for [C_16_H_32_N_8_O_4_Hg]^2+^ 301.113, [C_16_H_31_N_8_O_4_Hg]^+^ 601.217 and [C_16_H_32_N_8_O_4_HgCl]^+^ 637.194; found (pH 5&7) 301.109 [M]^2+^, 601.209 [M-H]^+^ and 637.184 [M + Cl]^+^. ^1^H NMR (600 MHz, D_2_O, 25°C, pD 5.4) δ 3.34 (br, 12H, NC*H*_2_- pendant arms), 2.96 (br, 6H, NC*H*_2_- backbone), 2.60 (br, 6H, NC*H*_2_- backbone) *(integrations are not exact due to significant overlap of peaks). ^13^C{^1^H} NMR (151 MHz, D_2_O, 25°C, pD 5.4) δ 174.31 (*C*=O), 54.92 (N*C*H_2_- pendant arms), 53.38 (N*C*H_2_- backbone), ^1^H NMR (600 MHz, D_2_O, 25°C, pD 7.4) δ 3.37 (s, 8H, NC*H*_2_- pendant arms), 2.85 (s, 8H, NC*H*_2_- backbone), 2.63 (s, 8H, NC*H*_2_- backbone). ^13^C{^1^H} NMR (151 MHz, D_2_O, 25°C, pD 7.4) δ 174.35 (*C*=O), 53.45 (N*C*H_2_- pendant arms), 50.39 (N*C*H_2_- backbone).

#### 2.3.2 [Hg(*p*-SCN-Bn-TCMC)]^2+^

The same protocol described above for TCMC was used with its bifunctional counterpart. Briefly, HgCl_2_ in D_2_O (0.019 M, 0.25 mL) was added to a solution of *p*-SCN-Bn-TCMC in D_2_O (0.017 M; 0.25 mL) giving a ligand/Hg^2+^ ratio of approx. 1:1.1, and the pD of the solution was adjusted to 5.4 and 7.4 using 1 M NaOD or 1 M DCl. The solution was heated for 1 h at 80 °C. For NOESY experiments, a higher concentration of 10^–2^ M metal complex in solution was used which was not soluble in D_2_O. As a result, DMSO-*d*_*6*_ was used as an NMR solvent to increase the solubility of the complex instead of D_2_O. These solutions were used for NMR and MS experiments. ESI-HRMS *m*/*z* calcd. for [C_24_H_37_N_9_O_4_HgS]^2+^ 374.619, [C_24_H_36_N_9_O_4_HgS]^+^ 748.232 and [C_24_H_37_N_9_O_4_HgSCl]^+^ 784.208; found (pH 5&7) 374.618 [M]^2+^, 748.229 [M-H]^+^ and 784.201 [M + Cl]^+^. ^1^H NMR (600 MHz, D_2_O, 25 °C, pD 5.4) δ 7.31–7.08 (m, 4H, Ar- C*H*), 3.58–3.36 (m, 4H, NC*H*_2_- pendant arms/backbone), 3.35–2.85 (m, 10H, NC*H*_2_- pendant arms/backbone), 2.80–2.25 (m, 11H, NC*H*_2_- pendant arms/backbone). ^13^C{^1^H} NMR (151 MHz, D_2_O, 25°C, pD 5.4) δ 174.23 (*C*=O), 130.79 (Ar- *C*H), 130.35 (Ar- *C*H), 125.98 (Ar- *C*H), 123.24 (Ar- *C*H), 59.33 (N*C*H_2_- pendent arm), 53.88 (N*C*H_2_- pendent arm), 53.46 (N*C*H_2_- pendent arm), 53.29 (N*C*H_2_- pendent arm), 50.61 (N*C*H_2_-backbone), 50.45 (N*C*H_2_-backbone), 50.34 (N*C*H_2_-backbone), 50.04 (N*C*H_2_-backbone), 46.85 (N*C*H_2_-backbone), 46.58 (N*C*H_2_-backbone), 46.50 (N*C*H_2_-backbone), 41.54 (N*C*H_2_-backbone). ^1^H NMR (600 MHz, D_2_O, 25°C, pD 7.4) δ 7.25–7.09 (m, 4H, Ar- C*H*), 3.54–3.39 (m, 4H, NC*H*_2_- pendant arms/backbone), 3.24–2.87 (m, 9H, NC*H*_2_- pendant arms/backbone), 2.78–2.33 (m, 12H NC*H*_2_- pendant arms/backbone). ^13^C{^1^H} NMR (151 MHz, 25°C, D_2_O) δ 174.23, (C=O) 130.34 (Ar- *C*H), 129.84 (Ar- *C*H), 125.99 (Ar- *C*H), 121.84 (Ar- *C*H), 53.92 (N*C*H_2_- pendent arm), 53.88 (N*C*H_2_- pendent arm), 53.35 (N*C*H_2_- pendent arm), 53.31 (N*C*H_2_- pendent arm), 50.62 (N*C*H_2_-backbone), 50.51 (N*C*H_2_-backbone), 50.34 (N*C*H_2_-backbone), 50.05 (N*C*H_2_-backbone), 46.86 (N*C*H_2_-backbone), 46.60 (N*C*H_2_-backbone), 46.51 (N*C*H_2_-backbone), 41.55 (N*C*H_2_-backbone), 31.58 (Bn- *C*H_2_- CH).

#### 2.3.3 [Hg(*p*-SCN-Bn-DOTA)]^2-^

A solution of *p*-SCN-Bn-DOTA (4.6 mg, 0.0069 mmol, 1.0 equiv.) in MeOH (0.2 mL) was added to a solution of HgCl_2_ (2.1 mg, 0.0076 mmol, 1.1 equiv.) in MeOH (0.2 mL). The reaction mixture was heated for 1 h at 80 °C. After 1 h of heating, a white precipitate was observed, and subsequently collected by centrifugation and dissolved in DMSO for MS experiments. ESI-HRMS *m*/*z* calcd. for [C_24_H_32_N_4_O_8_HgS]^+^ 752.1678; found 752.166 [M-H]^+^.

#### 2.3.4 [Hg(TCMC-PSMA)]^2+^

A solution of HgCl_2_ in deionized H_2_O (10 μL, 10^–2^ M) was added to a solution of TCMC-PMSA (10 μL, 10^–2^ M) which was diluted to a total volume of 100 μL (final concentration 10^–3^ M). This solution was used for HPLC and MS experiments. LC-MS *t*_*R*_ = 2.435 min, ESI-HRMS *m*/*z* calcd. for [C_57_H_82_N_14_O_13_SHg]^2+^ 702.281; found 702.200, [M]^2+^.

### 2.4 UV-vis spectrophotometry

Stock solutions (2 × 10^−3^ M) of TCMC, *p*-SCN-Bn-TCMC, and HgCl_2_ were prepared in deionized water. The pH was noted to be neutral (pH 7) for all stock solutions. A serial dilution of the stock was used to prepare solutions at 2 × 10^−5^ M. Control solutions of either the ligands or the metal ion were prepared by the addition of an aliquot (500 μL) of either the ligands or the metal solutions to deionized water (500 μL). For Hg^2+^ concentration-dependent studies, a HgCl_2_ stock solution (10^–4^ M) was prepared and added to a solution containing ligand (500 μL, 2 × 10^−5^ M) to give 0.2, 0.4, 0.6, 0.8, or 1.0 molar equivalent of Hg^2+^. Deionized water was then added such that the total volume per sample was consistent (1.0 mL). Data were collected in the range of 200–500 nm.

### 2.5 Fourier transform infrared spectroscopy

Hg-complexes of *p*-SCN-Bn-TCMC or TCMC were first prepared by adding a solution of ligand (3.3 mg, 0.0016–0.0027 mmol, 1.0 equiv.) in MeOH (0.2 mL) (and 10 μL of 1 M HCl—for TCMC only) to a solution of HgCl_2_ (0.5–0.81 mg, 0.0018–0.0030 mmol, 1.1 equiv.) in MeOH (0.2 mL). Upon addition, a precipitate immediately formed, and the reaction mixtures were heated for 1 h at 80°C, subsequently cooled to ambient temperature and centrifuged (14,000 rpm, 2 min). The filtrate was decanted, and the solid pellet was washed 3 times with MeOH and then air dried. ESI-MS confirmed the complex formation. Solid pellets for FTIR spectra collection were placed on a diamond lens and pressed using the force gauge of analysis.

### 2.6 Production of mercury-197 m/g

Production of ^197m/g^Hg^2+^ was achieved through proton irradiation of natural gold (Au) targets via the ^197^Au (p,n)^197m/g^Hg nuclear reaction at the TR13 (13 MeV) cyclotron at TRIUMF—Canada’s particle accelerator center, following previously published procedures, with calculated rate of productions of 4 MBq/µA·h for ^197m^Hg and 2.9 MBq/µA·h for ^197g^Hg (Chen et al., 2022). Briefly, Au targets were prepared by the addition of 200–270 mg of Au foil to a 10 mm diameter indent (0.25 mm deep) of a tantalum backing (1 mm in thickness) and melted thereon in a furnace at 1,250°C (Rd-G—RD Webb Company—Natick MA, United States). The Au target was dissolved in *aqua regia* (3 mL), and the solution was then loaded onto a prepared column of LN resin. ^197m/g^Hg^2+^ was eluted in 6 M HCl (4 mL) while the ^197^Au was retained on the resin. The ^197m/g^Hg^2+^ solution matrix was then exchanged to a 0.1 M HCl solution by multiple steps of evaporation and reconstitution. The final activity ranged from 90 to 140 MBq of ^197m/g^Hg^2+^ obtained as HgCl_2_ in 250–350 μL 0.1 M HCl. The radionuclide purity was evaluated using gamma (γ)-ray spectroscopy on an N-type co-axial high-purity germanium (HPGe) gamma spectrometer (CANBERRA, Mirion Technologies, Inc., San Ramon, CA, United States), calibrated with a 20 mL ^152^Eu and ^133^Ba source. Samples were prepared by mixing aliquots of ^197m/g^Hg^2+^ activity (1.2 MBq) with deionized water in a 20 mL glass vial to make a 20 mL sample and measured at a distance of 150 mm from the detector for 10 min, ensuring dead times were below 10%. Spectra were analyzed using Genie-2000 software, using the 133.98 keV (*I*_γ_ = 33.5%) and 164.97 keV (*I*_γ_ = 0.2618%) γ-lines of [^197m^Hg]Hg^2+^, and the 77.35 keV (*I*_γ_ = 18.7%) γ-line of [^197g^Hg]Hg^2+^ for activity calculations (“Live Chart of Nuclides. IAEA., 2023”). The radionuclidic purity was >99%.

### 2.7 ^197m/g^Hg^2+^ radiolabeling studies

As isothiocyanate-containing compounds are known to readily undergo hydrolysis (Kawakishi and Namiki, 1969), solutions were freshly prepared the day of use. Radiolabeling procedures closely followed those previously developed by our group (Randhawa et al., 2023). Stock solutions of TCMC, DOTA, *p*-SCN-Bn-TCMC, *p*-SCN-Bn-DOTA, and TCMC-PMSA (10^–3^ M) in deionized water (dilute HCl for TCMC) were used to prepare serial dilutions at ligand concentrations of 10^−4^, 10^−5^ M, 10^−6^ M, and 10^−7^ M which were diluted in deionized water. An aliquot (10 μL) of each ligand solution (or deionized water, for negative control) was diluted with sodium acetate (NaOAc) buffer (1 M; pH 5) such that the final reaction volume was 100 μL. An aliquot of [^197m/g^Hg]HgCl_2_ (1.0–1.2 MBq, 3–10 μL) was added and mixed gently at 80°C. Complex formation was monitored for each reaction by acquiring the non-isolated percentage radiochemical yield (%RCY) at varying time points (10–60 min). This was achieved firstly by quenching the reaction by extracting an aliquot (10 μL) of the reaction solution and adding it to an equal volume of dimercaptosuccinic acid (DMSA) solution (50 mM, pH 5, 10 μL). The quenched solution was gently mixed and analyzed by spotting a portion (10 μL) of the mixture onto the bottom of an iTLC-SG plate (1 cm × 10 cm, baseline at 1 cm) and then developed using DMSA solution (50 mM, pH 5) as the mobile phase. Under these conditions, the [^197m^Hg]Hg^2+^-complexes remain at the baseline (*R*_f_ = 0), while the unchelated, ‘free’ ^197m/g^Hg^2+^ migrates towards the solvent front (*R*_f_ = 1). TLC plates were analyzed on an Eckert & Ziegler AR-2000 TLC scanner and processed with Eckert & Ziegler WinScan software. Radiolabeling yields were calculated by integrating the peaks in the radio-chromatogram.

#### 2.7.1 TCMC-PMSA radio-HPLC

Solvent System—A: 90% H_2_O, 10% MeCN 6 mM NH_4_OAc; B: 10% H_2_O, 90% MeCN 6 mM NH_4_OAc; gradient—0% B (0–0.5 min), 0%–100% B (0.5–7 min), 100% B (7–8 min), 100%–0% B (8–9.5 min), 0% B (9.5–10 min). [^197m/g^Hg][Hg(TCMC-PSMA)]^2+^, *t*_*R*_ = 4.736 min, [^nat^Hg][Hg(TCMC-PSMA)]^2+^, *t*_*R*_ = 4.500 min and TCMC-PSMA, *t*_*R*_ = 4.561 min.

### 2.8 Glutathione (GSH) competition assay

GSH competition assay procedures closely followed those previously developed by our group (Randhawa et al., 2023). [^197m/g^Hg][Hg(*p*-SCN-Bn-TCMC)]^2+^ and [^197m/g^Hg][Hg (TCMC-PSMA)]^2+^ (prepared as described above) or radiolabeling controls (deionized water instead of the ligand) were added to a 50 mM *L*-glutathione (GSH) solution (1:22 *v*/*v* GSH:reaction solution dilution), and the mixtures were incubated at 37 °C over 24 h. The final GSH concentration was chosen to mimic *in vivo* conditions within cells (2.12 mM) (Dickinson and Forman, 2002). The proportion of intact radiolabeled complex was monitored over the course of 24 h using iTLC-SG and *L*-glutathione (50 mM) as the mobile phase. Under these conditions, uncomplexed ^197m/g^Hg^2+^ resulting from GSH transchelation traveled to the solvent front (*R*_f_ = 1) while intact [^197m/g^Hg][Hg(*p*-SCN-Bn-TCMC)]^2+^ or [^197m/g^Hg][Hg (TCMC-PSMA)]^2+^ remained at the baseline (*R*_f_ = 0).

### 2.9 Human serum stability assay

[^197m/g^Hg][Hg (*p*-SCN-Bn-TCMC)]^2+^ and [^197m/g^Hg][Hg (TCMC-PSMA)]^2+^ (prepared as described above) or radiolabeling controls (deionized water instead of the ligand) were diluted in human serum (1:1 *v/v* dilution), and the solutions were incubated at 37 °C over 24 h. The metal-complex stabilities were monitored over 24 h using SDS-PAGE. At each time point, an aliquot (10 μL) of the reaction mixture was mixed with Laemmli sample buffer (10 μL) and was directly loaded onto the SDS-PAGE gel. The SDS-PAGE was run at ambient temperature and 150 V until the dye front reached the resolving gel (1 h). Following electrophoresis, the gel was scanned with the radio-TLC scanner to determine the percentage of intact complex. The same protocol was used with free [^197m/g^Hg]Hg^2+^ and the ^197m/g^Hg^2+^ complexes diluted in phosphate-buffered saline (PBS) (5 µL; 1:1 *v/v* dilution) to assess their electrophoretic mobility.

### 2.10 LogD_7.4_ measurements

Aliquots of each ^197m/g^Hg^2+^ radiolabeled bioconjugate (10 μL) were added to a biphasic mixture of *n*-octanol (700 μL) and phosphate buffered saline (PBS, 700 μL, pH 7.4). The mixture was vortexed for 2 min at ambient temperature and then separated via centrifugation (10 min, 3,000 rpm). Aliquots of *n*-octanol (100 μL) and PBS (100 μL) were collected, and the activity in each portion was determined via gamma spectroscopy. LogD_7.4_ is defined as log_10_ [(activity in *n*-octanol phase)/(activity in buffer phase)].

### 2.11 Density functional theory calculations

Density functional theory (DFT) calculations were performed using the Gaussian 16 (Revision B.01) program package with the Becke, 3-parameter, Lee-Yang-Parr (B3LYP) functional (Becke, 1993; Becke, 2009; Frisch et al., 2016). Non-metallic atoms (C, H, N, O, and S) were modeled using the triple-ζ 6-311G** basis set (Krishnan et al., 1980; McLean and Chandler, 1980), while the Stuttgart Dresden (SDD) small-core effective core potential (ECP) with the associated SDD basis set was utilized for Hg^2+^ to include scalar relativistic effects (as obtained from Basis Set Exchange) (Häussermann et al., 1993; Feller, 1996; Schuchardt et al., 2007; Pritchard et al., 2019). Empirical dispersion was employed using Grimme’s dispersion correction with Becke-Johnson damping (D3-BJ) (Grimme et al., 2010; Grimme, Ehrlich, and Goerigk, 2011). Geometry optimizations were performed without imposing any symmetry constraints. All ligands and complexes were optimized in the gas phase and aqueous solution (dielectric constant ε = 78.36) using the polarizable continuum model with the integral equation formalism variant (IEFPCM), which creates solvent cavities *via* a set of overlapping spheres (Cossi et al., 2003). Frequency calculations indicated no imaginary frequencies were present at the optimized molecular geometries, which suggests that they are real minima of the respective potential energy surfaces. Single-point energy calculations were performed at the same level of theory. Unless specified, all calculations were performed at 25 °C and 1 atm. Initial input geometries were constructed using X-ray crystallographic data for [Hg(TCMC)]^2+^ (CCDC 241674), and all other structures were manually constructed based on the former data (Hancock, Reibenspies, and Maumela, 2004). Calculation results were visualised and interpreted using GaussView version 5.0.9 and VMD version 1.9.4a53 (Humphrey, Dalke, and Schulten, 1996; Dennington, Keith, and Millam, 2016). Root-mean-square deviations were calculated according to Eq. (1):

$$RMSD=\sqrt{\frac{1}{N}\sum_{i=1}^{n}\delta_{i}^{2}}$$ (1)

where δ is the difference in bond lengths (Å) of the respective systems.

### 2.12 Cell binding assay

#### 2.12.1 Radiotracer preparation

Radiolabeling of TCMC-PSMA (5 μL, 10^−4^ M) with [^197m/g^Hg]HgCl_2_ (0.5 MBq, 1.2 μL, 0.01 M HCl) was performed in NaOAc buffer (1 M, 43.8 μL, pH 5) at 80 °C. The reaction solution was allowed to stand for 1 h at 80 °C, and quantitative %RCY was confirmed by quenching an equal volume of the reaction mixture with DMSA (50 mM, pH 5) and monitored *via* iTLC-SG using DMSA (50 mM, pH 5.5) as the mobile phase. To prevent the radioactivity from sticking to the well plate, 10% DMSO was added to the reaction mixture.

#### 2.12.2 Receptor binding assay

*In vitro*, competition-binding assays were conducted as previously reported using LNCaP prostate cancer cells and Piflufolastat ^18^F (^18^F-DCFPyL) as the radioligand (H.-T. Kuo et al., 2018b). Briefly, LNCaP cells (200,000/well) were plated onto a 24-well poly-*D*-lysine coated plate for 48 h. Growth media was removed and replaced with HEPES buffered saline (50 mM HEPES, pH 7.5, 0.9% NaCl, 5% fetal bovine serum), and the cells were incubated for 1 h at 37°C. ^18^F-DCFPyL (0.1 nM) was added to each well (in triplicate) containing various concentrations (0.01 nM–10 µM) of both tested compounds ([^197 m/g^Hg][Hg (TCMC-PSMA)]^2+^ or [^nat^Hg][Hg(TCMC-PSMA)]^2+^). The assay mixtures were further incubated for 1 h at 37 °C with gentle agitation. The buffer and radioactive complex were removed, and cells were washed twice with cold HEPES buffered saline. To harvest the cells, 0.25% trypsin solution (400 µL) was added to each well, incubated for 10 min and then collected and measured. Radioactivity was measured on a PerkinElmer (Waltham, MA) Wizard2 2480 automatic gamma counter. Nonlinear regression analyses and inhibition constant (K_*i*_) calculations were performed using the GraphPad Prism 7 software.

### 2.13 Analysis/statistics

For stability (Figure 8) and 10^–4^ M labeling (Supplementary Figure S47) data, a 2-way ANOVA analysis was performed using Graphpad Prism software.

## 3 Results and discussion

### 3.1 Characterization of non-radioactive Hg^2+^ complexes

Non-radioactive Hg^2+^ complexes of TCMC and *p*-SCN-Bn-TCMC were studied to assess their ability to coordinate Hg^2+^ and investigate the effect of the bifunctional moiety on Hg^2+^ binding.

The 1D (^1^H and ^13^C{^1^H}) and 2D (^1^H-^1^H COSY, ^1^H-^13^C HSQC, and ^1^H-^13^C HMBC) NMR spectra were obtained for both TCMC and *p*-SCN-Bn-TCMC (Supplementary Figures S1−S8 and Supplementary Figures S17−S24, respectively) as well as for their corresponding Hg^2+^ complexes (Supplementary Figures S19−S16 and Supplementary Figures S25−S32, respectively) at different pH, herein the pH 7 coordination chemistry is discussed as it is biologically relevant and both pH’s gave comparable results. Mass spectra were collected for all the NMR samples, and complex formation was confirmed by the diagnostic ^nat^Hg^2+^ isotope pattern (Figure 2).

**FIGURE 2:**
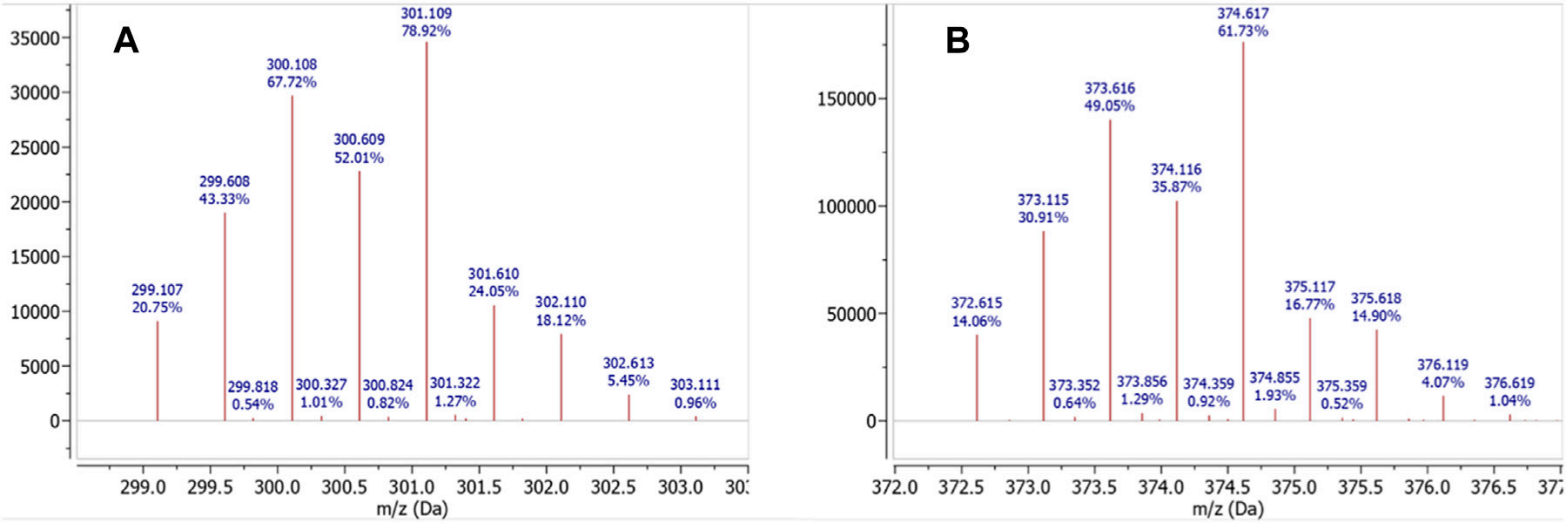
HRMS for **(A)** [Hg(TCMC)]^2+^ *m*/*z* calcd. for [C_16_H_32_N_8_O_4_Hg]^2+^ 301.113; found 301.109 [M]^2+^, and **(B)** [Hg(*p*-SCN-Bn-TCMC)]^2+^ *m*/*z* calcd. for [C_24_H_37_N_9_O_4_HgS]^2+^ 374.619; found 374.617 [M]^2+^.

The ^1^H NMR spectra of both [Hg(TCMC)]^2+^ and [Hg(*p*-SCN-Bn-TCMC)]^2+^ demonstrated evidence of metal binding.

**FIGURE 3:**
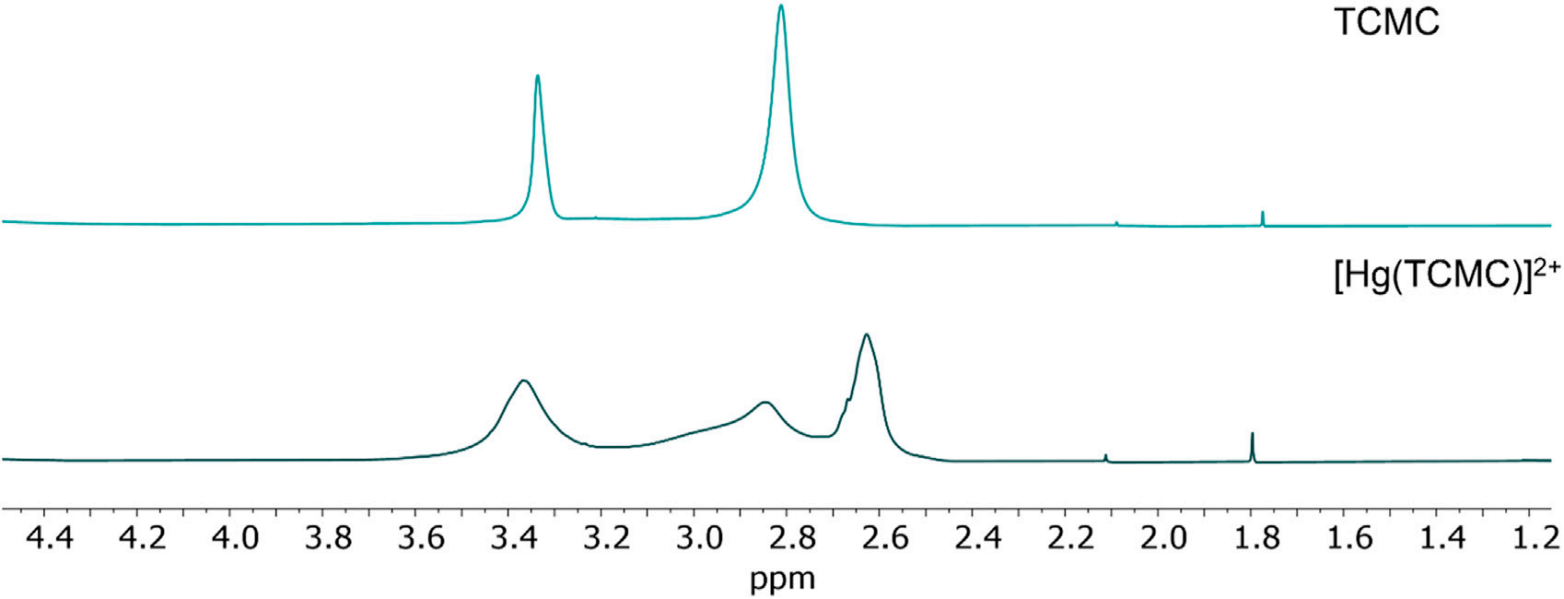
^1^H NMR in D_2_O (600 MHz, 25 °C) at pH 7 (pD 7.4) for TCMC (top) and the [Hg(TCMC)]^2+^ complex (bottom).

The hydrogens of the TCMC ligand (pH 7/pD 7.4) resonate as two singlets at δ = 3.34 ppm (NCH_2_ pendant arms) and δ = 2.81 ppm (NCH_2_ ring), indicating that the ligand adopts a highly symmetric conformation in solution. The [Hg(TCMC)]^2+ 1^H NMR spectrum displayed markedly broader peaks at δ = 3.37 ppm, δ = 2.85 ppm, and δ = 2.63 ppm in a 12H: 6H: 6H ratio. Upon comparison with the spectra of the free TCMC, several notable differences were observed. The NCH_2_ signals of the pendant arms (δ = 3.34 ppm), although significantly broadened, exhibited only slight deshielding, suggesting there may be little to no coordination of the amide arms, or rapid exchange too fast to be resolved on the NMR timescale. However, the signals characteristic of the cyclen ring underwent a distinct transformation, splitting into two enlarged singlets (δ = 2.96 and 2.90 ppm). The broadening of all NCH_2_ signals suggests fluxionality and conformational changes in the ring upon binding to the metal ion in solution (Figure 3). Additionally, the splitting of the singlet characteristic of the cyclen ring indicates that those NCH_2_ protons became non-magnetically equivalent after metal chelation, differentiating both sides of the macrocyclic ring.

The [Hg(TCMC)]^2+^ crystal structure has been previously reported, and Hg^2+^-coordination was observed with the four macrocyclic tertiary nitrogen atoms and two amide oxygen atoms forming a 6-coordinate structure (Hancock, Reibenspies, and Maumela, 2004). The ^1^H NMR spectrum does not match the expected integration and splitting based on the crystal structure reported in the literature. Variable-temperature (VT) ^1^H NMR experiments were conducted in the investigated temperature range (10–50 °C), and an increased broadness of the peaks could be observed at lower temperatures (Supplementary Figure S34). In contrast, a marked sharpening of the peaks was observed at increased temperatures (>40 °C), further suggesting the presence of a fluxional species in solution at ambient temperature, giving rise to the broadening in the peaks seen in Figure 3 (Atwood et al., 2006).

The ^13^C{^1^H} NMR spectrum of [Hg(TCMC)]^2+^ compared to TCMC (both at pH 7/pD 7.4, Supplementary Figures S6, S14, respectively) exhibits non-conformities in the carbon resonances. The ^13^C{^1^H} NMR chemical shifts of TCMC occur at δ = 174.0, 56.3, and 50.3 ppm for the amide and two aliphatic N-bound carbons, while the [Hg(TCMC)]^2+^ (pH 7/pD 7.4) spectrum displays a strong carbon resonance at δ = 174.35 ppm. Upfield, resonances were resolved: a strong signal at δ = 53.5 ppm and a weak signal at δ = 50.4. The signal corresponding to the amide carbon of TCMC is much weaker, while the corresponding metal complex displayed strong amide resonances in the same chemical shift region. This could be due to the fixed position of the pendant amide arms when bound to Hg^2+^, as opposed to the free rotations and multiple conformations available in the free ligand, resulting in a weakened ^13^C signal in the free ligand spectrum.

**FIGURE 4:**
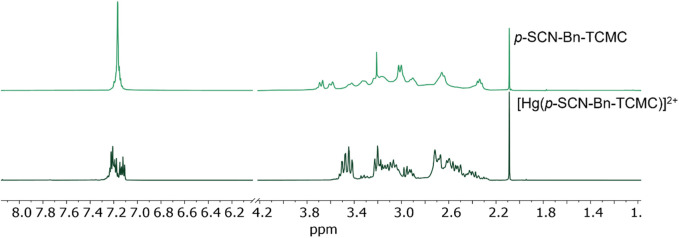
^1^H NMR in D_2_O (600 MHz, 25 °C) at pH 7 (pD 7.4) for *p*-SCN-Bn-TCMC (top) and the [Hg(*p*-SCN-Bn-TCMC)]^2+^ complex (bottom).

Several changes in the ^1^H NMR spectrum of [Hg(*p*-SCN-Bn-TCMC)]^2+^ are observed upon Hg^2+^ complexation. The *p*-SCN-Bn-TCMC ^1^H NMR spectrum (pH 7/pD 7.4) exhibits multiplet peaks at δ = 7.17 ppm, corresponding to the four benzyl protons, and several broad multiplets between δ = 3.77–2.24 ppm, corresponding to the aliphatic protons. Upon complexation, marked spectral changes appear. The benzyl protons are split into two multiplets centred at δ = 7.37 and 6.92 ppm. Peaks in the aliphatic region between δ = 3.53–2.09 ppm appear markedly sharper and separated compared to those of the free ligand with a slight downfield shift. This is likely due to the increased structural rigidity of the backbone upon metal complexation, leading to less rotational freedom on the NMR timescale, resulting in well-defined peaks (Figure 4). A marked contribution of the aromatic ring either through -NCS or π-interactions could also be inferred as seen by the splitting of the aromatic hydrogens in the metal-complex ^1^H NMR spectrum, as previously observed for benzyl-containing Hg^2+^ complexes (Randhawa et al., 2023).

Like [Hg(TCMC)]^2+^, VT-^1^H NMR experiments were undertaken for the [Hg(*p*-SCN-Bn-TCMC)]^2+^ complex between 10–50 °C (Supplementary Figure S36). Interestingly, with varying temperatures, no substantial changes in the NMR spectra were observed, supporting our hypothesis that a more rigid structure is formed in solution when Hg^2+^ is bound to *p*-SCN-Bn-TCMC.

Furthermore, the 2D NOESY spectra of [Hg(*p*-SCN-Bn-TCMC)]^2+^ (Supplementary Figure S33) was acquired to determine the spatial configuration of the ligand in solution and the possibility of the metal binding being affected by the benzyl isothiocyanate. NOESY correlations of the benzyl isothiocyanate and macrocyclic backbone protons were observed in both the [Hg (*p*-SCN-Bn-TCMC)]^2+^ and *p*-SCN-Bn-TCMC (Supplementary Figure S33) NMR spectra, suggesting that the macrocyclic and pendant arm protons and the benzyl protons would be in close proximity in solution.

Similarly to TCMC, the amide carbon resonance in the *p*-SCN-Bn-TCMC ^13^C{^1^H} NMR spectrum was not well-resolved (Supplementary Figure S18 (pH 5/pD 5.4) and Supplementary Figure S32 (pH 7/pD 7.4)); in comparison to the corresponding complex where a peak at δ = 174.23 ppm was observed in the ^13^C{^1^H} NMR spectrum of [Hg(*p*-SCN-Bn-TCMC)]^2+^ (Supplementary Figure S26 (pH 5/pD 5.4) and Supplementary Figure S30 (pH 7/pD 7.4)), suggesting an increase in structural rigidity upon metal complexation.

Unfortunately, no valuable insight could be drawn from the infrared (Supplementary Figures S39, 40) and UV-vis (Supplementary Figure S41) spectra as no distinct or diagnostic changes upon complexation were observed.

### 3.2 Radiolabeling with mercury-197 m/g

**FIGURE 5:**
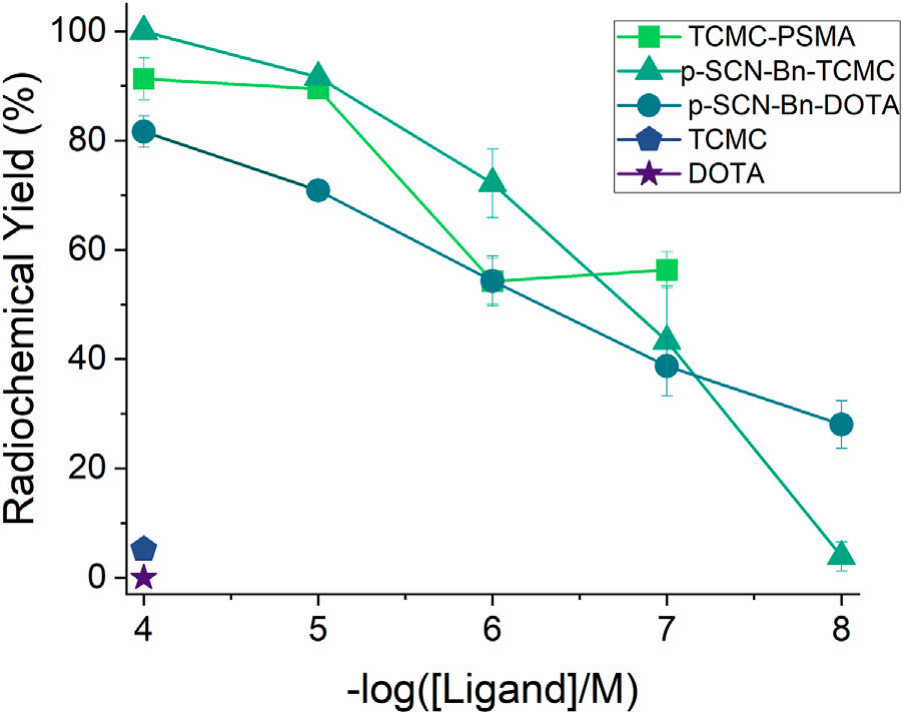
^197m/g^Hg^2+^ (1 MBq, 100 µL reaction volume) non-isolated radiochemical yields (RCY %) for the TCMC derivatives (TCMC, *p*-SCN-Bn-TCMC, and TCMC-PSMA) and DOTA derivatives (DOTA, *p*-SCN-Bn-DOTA) at ligand concentrations of 10^−4^ M at 1 h, 80 °C, pH 5 (*n* = 3).

**FIGURE 6:**
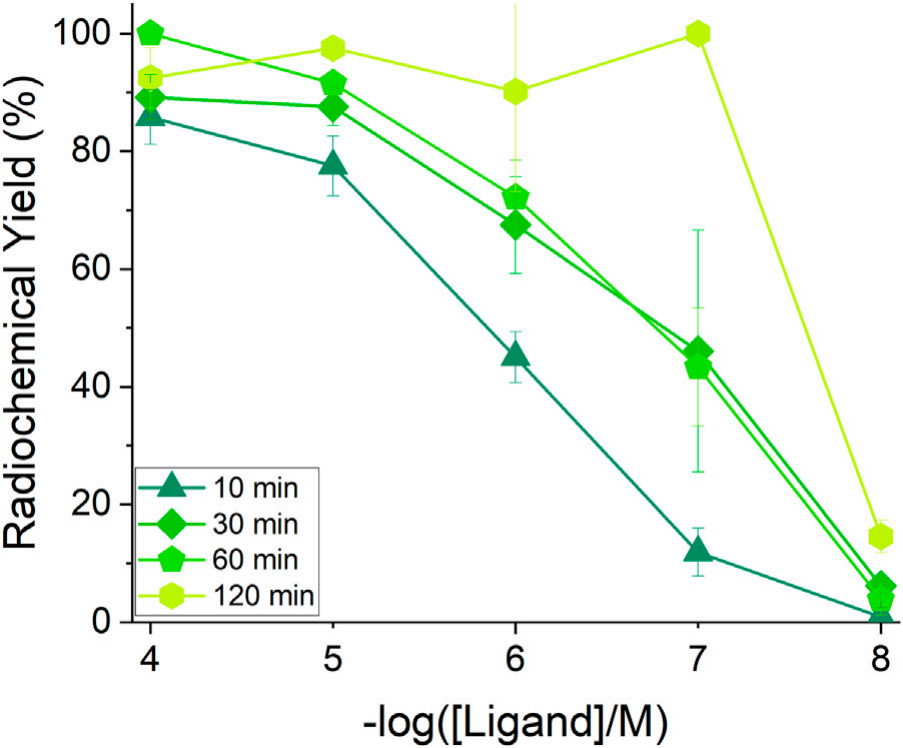
^197m/g^Hg^2+^ (1 MBq, 100 µL reaction volume) non-isolated radiochemical yields (RCY %) for *p*-SCN-Bn-TCMC radiolabeling reactions at pH 5 and 80 °C at varying ligand concentrations (10^−4^ M–10^−8^ M, *n* = 3) and reaction times (10, 30, 60 and 120 min).

^197m/g^Hg^2+^ radiolabeling studies of TCMC and *p*-SCN-Bn-TCMC were undertaken to assess the suitability of the ligands for radiopharmaceutical application. ^197m/g^Hg^2+^ radiolabeling of DOTA was also conducted for comparison with both TCMC and the bifunctional derivatives. No radiometal incorporation (radiochemical yield, RCY = 0%) was observed under any tested condition with DOTA (*C*_DOTA_ = 10^−4^ M, 1 h, ambient temperature or 80 °C, pH 5), as previously observed (Randhawa et al., 2023). ^197m/g^Hg^2+^ radiolabeling of TCMC resulted in a very moderate increase in radiometal incorporation (RCY = 5.2 ± 1.7%; *C*_TCMC_ = 10^−4^ M, 1 h, 80 °C, pH 5) (Figure 5; Supplementary Figure S45). Strikingly, its bifunctional counterpart, *p*-SCN-Bn-TCMC, exhibited a high affinity to [^197m/g^Hg]Hg^2+^, achieving a RCY of 85.8 ± 4.7% (*C*_*p*-SCN-Bn-TCMC_ = 10^−4^ M, 10 min, 80 °C, pH 5; Figure 6; Supplementary Figure S46). However, elevated temperatures were required to induce quantitative ^197m/g^Hg^2+^ incorporation, as insignificant radiolabeling was observed for reactions conducted at 37 °C at high ligand concentration. Ambient temperature reactions were consequently not attempted.

Concentration-dependent radiolabeling of *p*-SCN-Bn-TCMC was performed, and RCY values were tracked over 60 min or 120 min (Figure 6, S45). Under reaction conditions of pH 5, 1 h and 80 °C, RCYs were determined to be 100 ± 0.0%, 91.6 ± 0.4%, 72.2 ± 6.3%, 43.3 ± 10.1% and 3.9 ± 2.7% (*n* = 3) for ligand concentrations of 10^−4^, 10^−5^, 10^−6^, 10^−7^, and 10^−8^ M, respectively.

Given *p*-SCN-Bn-TCMC’s ability to incorporate radio-mercury compared to its non-bifunctional analogue, ^197m/g^Hg^2+^ radiolabeling studies with *p*-SCN-Bn-DOTA were also attempted to investigate if this marked difference can be attributed to the presence of the sulfur-containing isothiocyanate moiety. Interestingly, *p*-SCN-Bn-DOTA (Figure 5) was also able to incorporate ^197m/g^Hg^2+^ moderately well but not at the capacity of *p*-SCN-Bn-TCMC, exhibiting a RCY after 1 h at 80°C (*C*_*p*-SCN-Bn-DOTA_ = 10^−4^ M, pH 5) of 70.9 ± 1.1%, compared to 100 ± 0.0% for *p*-SCN-Bn-TCMC under the same conditions.

The significant disparity in radiometal incorporation yields between the non-bifunctional ligands and those containing the benzyl isothiocyanate group suggests that the isothiocyanates present in the bifunctional ligands play a crucial role in enabling efficient complexation of [^197m/g^Hg]Hg^2+^. Given sulfur’s high affinity to selectively coordinate Hg^2+^ (Atwood et al., 2006; Riccardi et al., 2013), the ability of ^197m/g^Hg^2+^ to coordinate through the isothiocyanate was investigated further by radiolabeling phenyl isothiocyanate alone. ^197m/g^Hg^2+^ radiolabeling of phenyl isothiocyanate resulted in 85.7 ± 3.7% RCY (Supplementary Figure S47) under the same labeling conditions of the *p*-SCN-Bn-TCMC (1 h at 80 °C, 10^–4^ M ligand, pH 5), thus substantiating the relevance of this moiety in metal binding.

The binding mode of the Hg^2+^ formed complexes with both TCMC and *p*-SCN-Bn-TCMC complex inferred from the NMR suggests coordination through the macrocyclic backbone. However, radiolabeling results indicated a potential alternative binding mechanism at the nanomolar scale of radiochemical reactions and it is evident that the isothiocyanate plays a role in binding. An alternative coordination product may arise, such as the formation of a bis-substituted linear complex, binding the ^197m/g^Hg^2+^ via the isothiocyanates. This is highly probable due to Hg^2+^’s preference to form linear complexes (Kaupp and Von Schnering, 1994; Olsen et al., 2015). The explanation for the disparity in the metal-complexation for NMR and radiolabeling can be attributed to the significantly different metal-to-ligand ratios (NMR = metal:ligand; 1:1, radiolabeling = 1:1,000–1,00,000) in the experimental conditions which can make a direct comparison of their results difficult. The radiolabeling reactions occur under dilute conditions, with the chelator present in significant excess compared to the radiometal (chelator concentration <10^−4^ M, radiometal concentration 10^−9^ M or lower), which would permit the bis-substituted complex to form.

Since *p*-Bn-SCN-TCMC was able to effectively incorporate ^197m/g^Hg^2+^, preparation and isolation of the TCMC-PSMA bioconjugate *via* sulfur containing thiourea bond formation was pursued. Afterwards, radiolabeling of the bioconjugate to form [^197 m/g^Hg][Hg(TCMC-PSMA)]^2+^ (80 °C, *C*_TCMC-PSMA_ = 10^−4^ M, pH 5) (Figure 5) was conducted, the radiolabeling yields for the bioconjugate were statistically lower than the RCYs of *p*-SCN-Bn-TCMC at early time points under the same conditions (10 min: 65.6 ± 1.8% vs. 85.8 ± 4.6%, 30 min: 68.2 ± 0.2% vs. 89.2 ± 3.9%, for TCMC-PSMA and *p*-SCN-Bn-TCMC, respectively) (Supplementary Figure S46); however, as the reaction was left to proceed the RCY of the bioconjugate increased to 91.3 ± 3.8% after 60 min (Supplementary Figure S45). The slower observed radiolabeling kinetics may be attributed to the introduction of steric hindrance from the presence of the PSMA moiety. Furthermore, concentration-dependent radiolabeling was performed with TCMC-PSMA. Under reaction conditions of pH 5, 1 h at 80 °C, RCYs were determined to be 89.5 ± 0.5%, 54.2 ± 4.2%, 56.3 ± 3.4% for bioconjugate concentrations of 10^−5^, 10^−6,^ and 10^−7^ M, respectively, providing, an apparent molar activity of approximately 0.089 MBq/nmol for the bioconjugate (Figure 5). In comparison to the reported [^212^Pb][Pb(TCMC-PSMA)]^2+^ molar activity of 0.5–2.4 MBq/nmol (Stenberg et al., 2022), a 100-fold difference is observed to the molar activity in this study. Molar activities of 0.5 MBq/nmol and higher are generally needed for preclinical studies. Therefore, the relateively low apparent molar activity of the Hg-TCMC-PSMA tracer would hinder our ability to advance the tracer to *in vivo* studies. Overall, the ^197m/g^Hg^2+^ radiolabeling efficiency of the thiourea-containing TCMC-PSMA compound was lower than the bifunctional isothiocyanate-containing chelator *p-*SCN-Bn-TCMC alone. These results further support the hypothesis that the ^197m/g^Hg^2+^ may coordinate through the isothiocyanate at the radiolabeling scale. However, radiolabeling was still achieved in the presence of the sulfur containing thiourea, as these functional groups have also been documented in the literature to coordinate Hg^2+^ (Lin et al., 2010; Olsen et al., 2015; Li et al., 2023).

In addition to monitoring the radiolabeling of the TCMC-PSMA bioconjugate through instant thin layer chromatography (iTLC), the formation of the ^197m/g^Hg^2+^ complex was confirmed through reverse-phase radio-high performance liquid chromatography (RP-radio-HPLC) (for experimental conditions and optimization, see Supplementary Material, Figure 7; Supplementary Figure S42). Previous to these efforts, radio-HPLC of ^197m/g^Hg^2+^-tracers in our group has been unsuccessful (Randhawa et al., 2023) due to the ‘sticky’ nature of [^197m/g^Hg]Hg^2+^. The [^197 m/g^Hg][Hg(TCMC-PSMA)]^2+^ complex exhibited a broad peak with a retention time equal to 4.74 min, which was corroborated by co-injection of the non-radioactive complex (retention time of 4.50 min; offset due to detector set-up); the success of the elution could be attributed to the lipophilicity of the tracer. The broadness of the peak could be due to the stickiness of the radiotracer but can also indicate the formation of a fluxional product.

**FIGURE 7:**
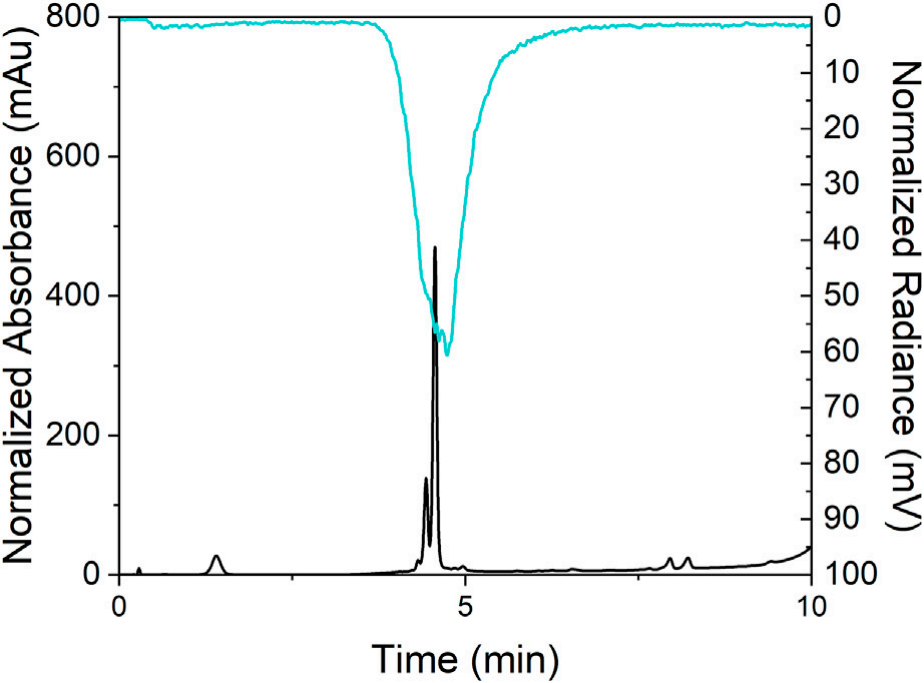
The radio-HPLC trace of [^197m/g^Hg][Hg(TCMC-PSMA)]^2+^ labeling reaction (top, *t*_R_ = 4.74 min) and the UV-HPLC chromatograph of prepared complex [^nat^Hg][Hg(TCMC-PSMA)]^2+^ (bottom, *t*_R_ = 4.50 min) co-injected.

### 3.3 Kinetic inertness of radiolabeled complexes

The *in vitro* kinetic inertness of both [^197m/g^Hg][Hg(*p*-SCN-Bn-TCMC)]^2+^ and [^197m/g^Hg][Hg(TCMC-PSMA)]^2+^, was assessed in human serum and glutathione (GSH) using previously established methods (Randhawa et al., 2023).

**FIGURE 8:**
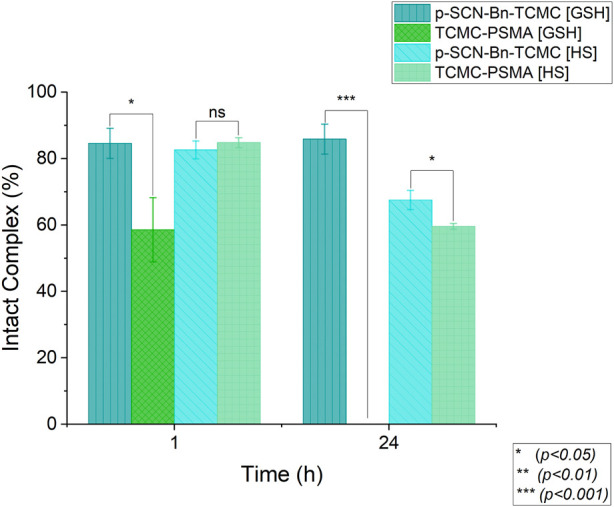
Human serum (HS) and glutathione (GSH) integrity of [^197m/g^Hg][Hg (*p*-SCN-Bn-TCMC)]^2+^ and [^197m/g^Hg][Hg (TCMC-PSMA)]^2+^ (∼0.009 MBq/nmol, 100 µL reaction volume, *n* = 3) at 37 °C over 24 h. Error bars represent the SD, a two-way ANOVA was used to calculate the significant differences.

GSH was incubated with the [^197 m/g^Hg]Hg^2+^-complexes at 37 °C and analyzed at 1 h and 24 h by radio-iTLC. The percent intact complex of [^197m/g^Hg][Hg(*p*-SCN-Bn-TCMC)]^2+^ was determined to be 84.6 ± 4.5% after 1 h, and remained relatively unchanged after 24 h (85.9 ± 4.5%) (Figure 8). This indicates the formation of a kinetically inert complex when challenged against GSH. [^197m/g^Hg][Hg(TCMC-PSMA)]^2+^, conversely, degraded significantly after 1 h (58.6 ± 9.6%), and had completely degraded after 24 h, demonstrating poor kinetic inertness against GSH.

Sodium dodecyl sulfate-polyacrylamide gel electrophoresis (SDS-PAGE) was used to determine the kinetic inertness of [^197m/g^Hg][Hg(*p*-SCN-Bn-TCMC)]^2+^ and [^197m/g^Hg][Hg(TCMC-PSMA)]^2+^ against human serum over 24 h at 37°C.

As shown in Figure 8, the percent intact complex in human serum was determined at 1 h and 24 h to be 82.0 ± 2.7% and 67.5 ± 2.9% for [^197 m/g^Hg][Hg(*p*-SCN-Bn-TCMC)]^2+^, and 84.8 ± 1.4% and 59.6 ± 0.9% [^197 m/g^Hg][Hg(TCMC-PSMA)]^2+^, respectively. These results indicate that both [^197 m/g^Hg][Hg(*p*-SCN-Bn-TCMC)]^2+^ and [^197 m/g^Hg][Hg(TCMC-PSMA)]^2+^ are equally moderately kinetically inert against human serum. The drop in the percentage of intact ^197m/g^Hg^2+^ complex over 24 h is likely related to the presence of biologically relevant substrates (most likely sulfur-rich proteins) that compete and displace the chelator-bound metal ion, indicating *in vivo* “free” (endogenous protein-bound) ^197m/g^Hg^2+^ will be present. Taken together, these results demonstrate the overall integrity of [^197 m/g^Hg][Hg(TCMC-PSMA)]^2+^ was found to be comparatively inferior to that of [^197 m/g^Hg][Hg(*p*-SCN-Bn-TCMC)]^2+^ as a possible result of the difference in the coordination environment.

### 3.4 logD_7.4_ measurements of the bioconjugate

The hydrophilicity of the radiolabeled bioconjugate was evaluated by measuring the partition coefficient between *n*-octanol and phosphate-buffered saline (PBS, 0.01 M, pH 7.4). The radiolabeled bioconjugate [^197 m/g^Hg][Hg(TCMC-PSMA)]^2+^ was found to have a logD_7.4_ value of −1.21 ± 0.2, indicating the tracer is moderately lipophilic, which is in agreement with other PSMA tracers found within the literature (Han et al., 2017).

### 3.5 Density functional theory calculations

**FIGURE 9:**
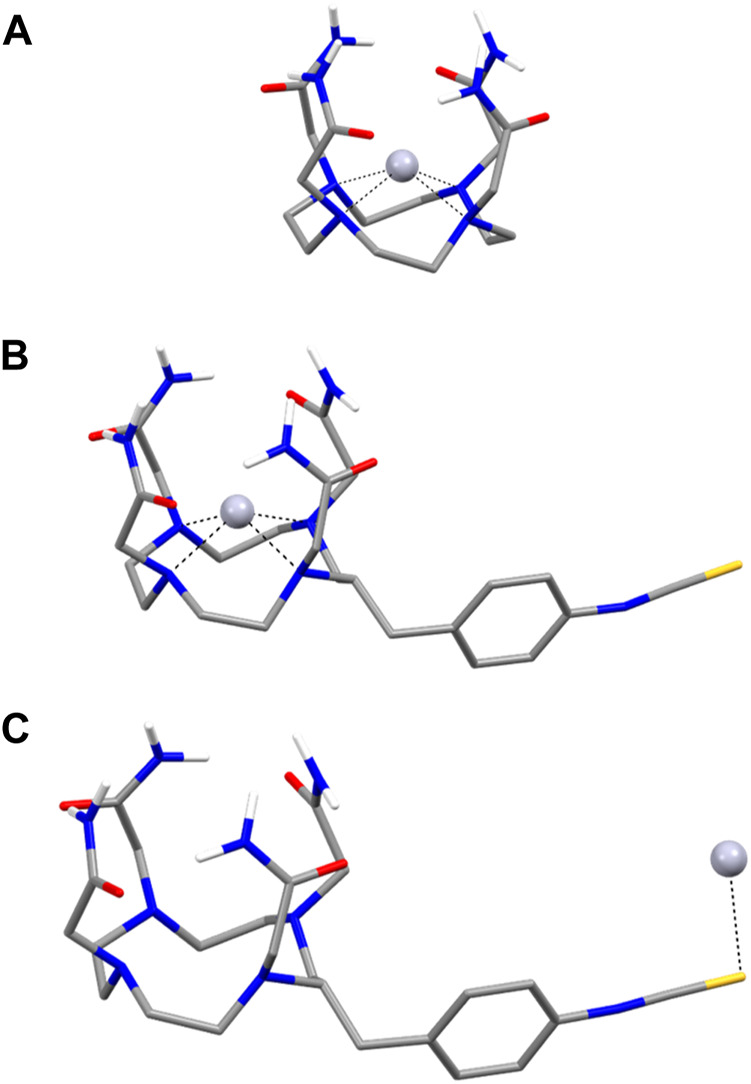
Lowest energy B3LYP-D3/6-311G**/SDD (Hg)/IEFPCM optimized geometries of **(A)** [Hg(TCMC)]^2+^, **(B)** [Hg-*p*-SCN-Bn-TCMC)]^2+^ and **(C)** TCMC-NCS-Hg with complexation at the isothiocyanate. Carbon-bonded hydrogen atoms have been omitted for clarity.

In lieu of single-crystal X-ray diffraction data, density functional theory (DFT) calculations were employed to further elucidate the coordination chemistry of the aforementioned commercial chelators with Hg^2+^. Additionally, the most likely aqueous solution structures of the Hg^2+^ complexes were calculated in order to gain insight into the potential binding modes of the radiolabeled bioconjugates *in vivo*, which has become an increasingly utilized tool in radiocomplex design (Läppchen et al., 2018; Brown et al., 2019; Holland, 2020; Davey, Forsyth, and Paterson, 2022; Kelderman et al., 2022). For all ligands and Hg^2+^ complexes, the calculated structures were optimized at the B3LYP-D3/6-311G**/SDD (Hg) level of theory at both 25 °C and 80 °C to investigate whether significant differences result in the resultant coordination chemistries under radiolabeling temperature conditions. Representations of the coordination geometries modeled in aqueous solvent using the polarizable continuum model with the integral equation formalism variant (IEFPCM), which creates solvent cavities via a set of overlapping spheres, are outlined in Figure 9.

At both 25 °C and 80 °C, the lowest energy [Hg(TCMC)]^2+^ complex is 4-coordinate, bonding through the macrocyclic N atoms (average Hg-N bond length = 2.277 Å (25 °C and 80 °C)) with no association observed with the pendant arm amides (average Hg-O bond length = 3.728 Å (25 °C and 80 °C)) confirming the NMR complex results (*vide supra*). Instead, the amides exhibit intermolecular hydrogen bonding, which seemingly helps to stabilize the resulting complex. The interplanar Hg-N and Hg-amide distances, as defined by the distance between the Hg^2+^ cation and centroids calculated from the 4 macrocyclic N atoms and 4 amide O atoms, respectively, are 0.877 Å and 1.533 Å at both temperatures, indicating a stronger association for the macrocyclic ring than the pendant donor atoms for the Hg^2+^ cation. The coordination of the [Hg(TCMC)]^2+^ complex calculated by DFT differs from that of the previously reported crystal structure mentioned above. This could be a result of the differences in the stabilization of the complex in the solution phase compared to the solid phase.

The lowest energy conformer for the bifunctional derivative is also a 4-coordinate complex, with slightly longer average Hg-N bond lengths (2.295 Å) and Hg-O bond lengths (3.769 Å). The interplanar Hg-N (0.906 Å) and Hg-amide (1.524 Å) distances are relatively similar to the TCMC complex. Root mean square deviation of 0.04 Å was found between both complexes, indicating minimal structural distortion between the two coordination geometries due to the bifunctional isothiocyanate arm. Much higher degrees of distortion were found between gas and solution structures, which may indicate that solvent effects play a significant role in stabilizing these complexes.

The electrostatic potential maps of [Hg(TCMC)]^2+^ and [Hg (*p*-SCN-Bn-TCMC)]^2+^ in the presence of water at both temperatures are shown in Supplementary Figure S48. The [Hg (TCMC)]^2+^ exhibits a relatively even positive surface charge distribution, with calculated dipole moments of μ = 2.98 Debye (D) at both 25°C and 80°C. This agrees with the relatively more symmetric nature of the resulting complex, as seen in the NMR spectra. Conversely, the calculated dipole moments of [Hg(*p*-SCN-Bn-TCMC)]^2+^ (μ = 23.4 D) demonstrate a much more polarized, asymmetrically charged complex. This also agrees with the highly asymmetric structure suggested by NMR experiments (*vide supra*).

Due to the incongruous radiolabeling results presented between the TCMC ligand and the bifunctional version, we sought to calculate the Gibbs free energies and Gibbs free enthalpies of formation for both chelators with Hg^2+^, to gain insight into which possible modes of coordination were most stable. Thermodynamic determination of Hg^2+^ solvation-free energies and binding constants were not considered in these calculations due to the use of an implicit solvation model (Devarajan et al., 2018; Asaduzzaman et al., 2019) as well as theoretical difficulty in calculating log *K* values for charged species. Free energies of formation (Δ*G*°) and free enthalpies of formation (Δ*H*°) were calculated for [Hg (TCMC)]^2+^ at both temperatures (Eq. (2)).

$$L+{\left[\text{Hg}{\left(H_{2}O\right)}_{6}\right]}^{2+}\;\to \;{\left[\text{Hg}\left(L\right)\right]}^{2+}+6H_{2}O$$ (2)

At both investigated temperatures, the formation of [Hg (TCMC)]^2+^ demonstrated negative Δ*G*° values (−36.3 kcal/mol and −36.1 kcal/mol at 25 °C and 80 °C, respectively), indicating that the formation of the complex is a thermodynamically favorable process under both conditions. However, at both temperatures, positive enthalpy values were obtained (4.38 and 4.57 kcal/mol, respectively), indicating a disfavoured thermodynamic process under these conditions. This may help to explain the lack of radiolabeling efficiency for this chelator with Hg^2+^.

The binding of Hg^2+^ to *p*-SCN-Bn-TCMC was evaluated both at the macrocycle cavity and the isothiocyanate functional group. The formation of the macrocyclic complex was significantly more stable at 25 °C (Δ*G*° = −146.5 kcal/mol and Δ*H*° = −107.2 kcal/mol) compared to binding through the isothiocyanate (Δ*G*° = −87.7 kcal/mol and Δ*H*° = −44.5 kcal/mol). A competition is likely formed between end-on isothiocyanate binding and binding through the macrocycle. We observe this competition experimentally, as the macrocycle bound product is formed at the NMR scale, and the isothiocyanate bound product is formed at the radiolabeling scale (*vide supra*). The computational finding agrees with the NMR data; however, due to the dilute conditions under which radiolabeling experiments are performed, the correlation to computational results is difficult.

### 3.6 Cell binding assay

**FIGURE 10:**
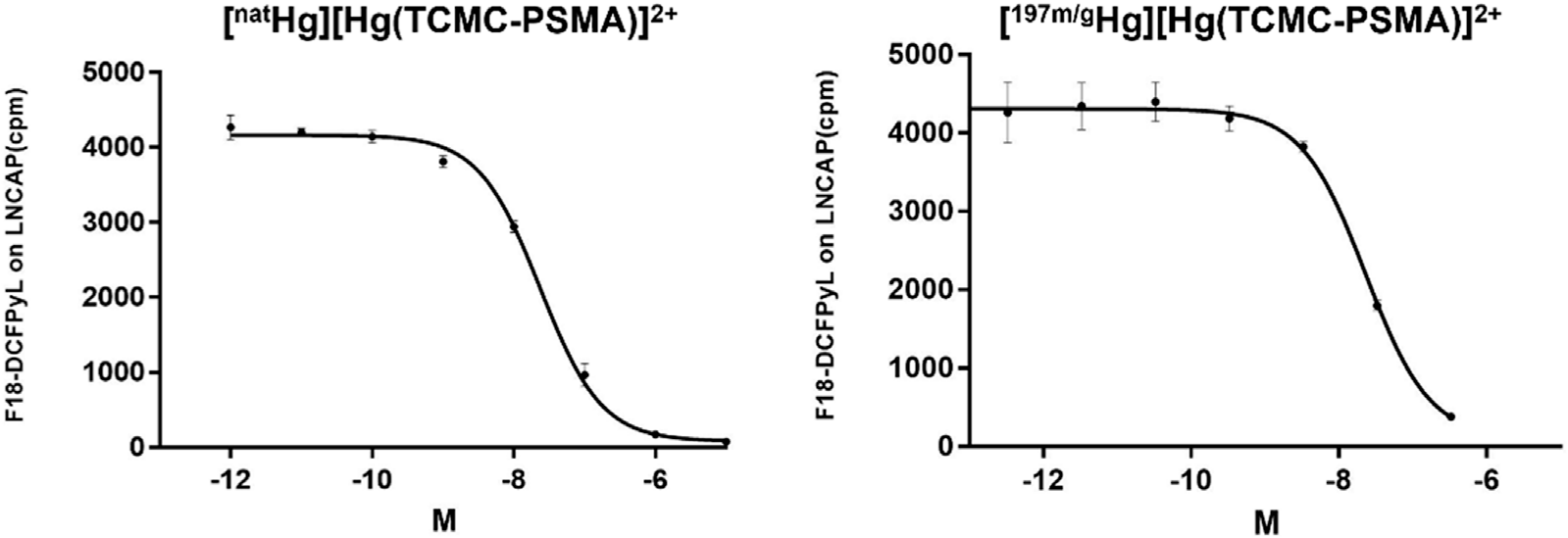
Titration of [^nat^Hg][Hg(TCMC-PSMA)]^2+^ as a competitor for ^18^F-DCFPyL constant 0.1 nM, *K*_*i*_ = 19.6 nM (*left*)*,* Titration of [^197 m/g^Hg][Hg(TCMC-PSMA)]^2+^ as a competitor for ^18^F-DCFPyL constant 0.1 nM, *K*_*i*_ = 19.0 nM (*right*); Conditions: LNCaP prostate cancer cells (200,000 cells/well), incubated for 1 h at 37 °C, containing a final 1% DMSO concentration.

To determine the ability of [^197 m/g^Hg][Hg(TCMC-PSMA)]^2+^ to bind to the prostate cancer cell surface receptors on LNCaP cells, cell binding assays with both [^197 m/g^Hg][Hg(TCMC-PSMA)]^2+^ (10,000-fold excess of non-complexed ligand present) and [^nat^Hg][Hg(TCMC-PSMA)]^2+^ (all ligands were complexed with Hg^2+^) were conducted. Both Hg-tracers successfully inhibited binding of the ^18^F-DCFPyL to PSMA in a dose-dependent manner (Figure 10) with resultant calculated *K*_i_ values of 19.0 and 19.6 nM, respectively. Comparing this value to that of [^177^Lu]Lu-PSMA-617 (*K*_i_ = 0.24 ± 0.06 nM) (H. T. Kuo et al., 2018a), a 100-fold difference in the *K*_i_ is observed.

The *K_i_* values were calculated based on the 511 keV γ line of ^18^F. However, the low energy γ lines of ^197m/g^Hg were also monitored in the [^197 m/g^Hg][Hg(TCMC-PSMA)]^2+^ assay (data not plotted). A dose-dependent increase in the activity of ^197m/g^Hg was measured, confirming that the displacement of [^18^F]-DCFPyL was directly related to the presence of higher concentrations of tracer. Notably, the similar *K*_i_ values for both radioactive ([^197 m/g^Hg][Hg(TCMC-PSMA)]^2+^; 19.6 nM) and non-radioactive ([^nat^Hg][Hg(TCMC-PSMA)]^2+^; 19.0 nM) complexes show that the 10,000-fold excess of unlabeled TCMC-PSMA in the radioactive tracer mixture does not significantly affect the receptor binding affinity compared to the non-radioactive tracer which was prepared in a 1:1 metal-ligand ratio (Figure 10). This result is significant, as it suggests if the radiolabeling of TCMC-PSMA likely involves the sulfur containing thiourea and not just the macrocyle backbone, the biological activity of the Hg-TCMC-PSMA is not affected by the potiential binding mode/location of Hg^2+^ on the TCMC-PSMA molecule. The 100-fold difference in *K*_i_ values between the Hg-TCMC-PSMA tracer and the previously studied Lu-PSMA-617 (H. T. Kuo et al., 2018b) tracer may be a result of the difference in chelator and overall charge of the complexes.

### 3.7 Conclusion

A fundamental investigation of cyclen-based commercial chelators (TCMC and DOTA) and their commercial bifunctional counterparts bearing *para-*benzyl-isothiocyanate functional groups (*p*-SCN-Bn-DOTA and *p*-SCN-Bn-TCMC) was conducted to examine their coordination chemistry and radiolabeling capabilities with [^197 m/g^Hg]Hg^2+^. Radiolabeling studies demonstrated TCMC and DOTA have poor RCY (0%–6%) even under harsh conditions (1 h, pH 5 and 80°C). In sharp contrast, [^197 m/g^Hg]Hg^2+^ labeling of the bifunctional derivatives *p*-SCN-Bn-TCMC and *p*-SCN-Bn-DOTA achieved yields of 91.6 ± 0.1% and 70.9 ± 1.1%, respectively, under the same conditions. *p*-SCN-Bn-TCMC was moderately kinetically inert against human serum (67.5 ± 2.9% over 24 h) and glutathione (85.87 ± 4.5% over 24 h). The corresponding non-radioactive metal complexes were evaluated using NMR spectroscopy and DFT calculations to determine the differences in labeling between the commercial chelators and their bifunctional counterparts. Both NMR spectra of TCMC and *p*-SCN-Bn-TCMC highlight binding for the Hg^2+^ through the core backbone framework. DFT studies demonstrate binding of the Hg^2+^ within the backbone is indeed the thermodynamically stable product. However, competition can form between isothiocyanate binding and binding through the macrocycle, which was experientially observed. The isothiocyanate bound coordination product was dominant at the radiochemical scale as, in comparison, the macrocycle bound product was seen at the NMR scale, agreeing with the DFT result. The TCMC-PSMA bioconjugate derivative was synthesized and labeled with ^197m/g^Hg^2+^ under the same conditions, resulting in an apparent molar activity of 0.089 MBq/nmol; however, when challenged with human serum (59.6% ± 0.9% over 24 h) and glutathione (0% over 24 h), significant degradation of the tracer was observed. [^197m/g^Hg]Hg-TCMC-PSMA was then subjected to cell binding assays, resulting in a *K*_i_ of 19.0–19.6 nM. This work provides essential details for the advancement of ^197m/g^Hg^2+^ radiopharmaceuticals, demonstrating the need for the development of specific and custom chelators for these exotic soft radiometals, as the use of these commercial chelators is not feasible for future advancement of the theranostic pair ^197m/g^Hg^2+^ in nuclear medicine applications due to the TCMC-PSMA bioconjugate’s poor *in vitro* stability against HS and GSH, suboptimal apparent molar activity and deficient *K*_i_ values. In particular, attention needs to be taken when designing bifunctional chelators for radiomercury, as the isothiocyanate (-NCS) bifunctional handle may interfere with radiolabeling even after conjugation to a biomolecule targeting vector (to form the resulting thiourea). This study also demonstrates the potential impact of introducing sulfur atoms into the chelator construct, which may act as appropriate donor atoms for ^197m/g^Hg^2+^ chelation. Consequently, current and future work in our group is focused on designing sulfur-rich macrocyclic chelators, such as NS_4_ and NS_4_-BA, which show improved ^197 m/g^Hg^2+^ incorporation yields. Bifunctional derivatives which incorporate the isothiocyanate group (or any sulfur atoms) as the bioconjugation handle should be avoided—given sulfur’s propensity to interfere with mercury coordination.

## Data Availability

The raw data supporting the conclusion of this article will be made available by the authors, without undue reservation.

## Footnotes

1 A committee of international experts has determined after careful review of her work that Lise Meitner did not discover Auger electrons but may instead have observed conversion electrons (CEs), as such we have coined the term ‘Meitner-Auger electron (MAE) emitter’ for such isotopes that may be used for radiopharmaceutical therapy (RPT) which emit both Auger electrons and CEs—since both the Auger and CEs contribute to therapeutic dose. We hope that this is respectful of the Auger nomenclature while still giving credit to Meitner’s contributions to this field.
