# Supplementary material for: Exploration of commercial cyclen-based chelators for mercury-197 m/g incorporation into theranostic radiopharmaceuticals
Source: Front Chem. 2024 Feb 8;12:1292566. doi: 10.3389/fchem.2024.1292566 (PMC10881723; doi:10.3389/fchem.2024.1292566)
Supplement: Supplementary file 1 [file DataSheet1.PDF]

Supplementary Material

Exploration of Commercial Cyclen-Based Chelators for Mercury-197m/g Incorporation into Theranostic Radiopharmaceuticals

Parmissa Randhawa[a,b], Imma Carbo-Bague[a,b], Patrick R. W. J. Davey[a,b], Shaohuang Chen[a,b], Helen Merkens[c], Carlos F. Uribe[c], Chengcheng Zhang[c], Marianna Tosato[a,b], François Bénard[c], Valery Radchenko[b,d], Caterina F. Ramogida[a,b]*

[a] Department of Chemistry, Simon Fraser University, 8888 University Drive, Burnaby, British Columbia V5A 1S6, Canada

[b] Life Sciences Division, TRIUMF, 4004 Wesbrook Mall, Vancouver, British Columbia V6T 2A3, Canada

[c] Department of Molecular Oncology, BC Cancer, Vancouver, British Columbia, V5Z 1L3.

[d] Department of Chemistry, University of British Columbia, 2036 Main Mall, Vancouver, British Columbia V6T 1Z1, Canada

* **Corresponding author**: cfr@sfu.ca; https://www.sfu.ca/chemistry/department/faculty-staff/profiles/research-faculty/cfr.html

Twitter: Caterina F. Ramogida: @CaterinaRamogi1; Parmissa Randhawa: @parmissaran; Imma Carbo-Bague:@icbague; Marianna Tosato: @mariannatosato; Patrick R. W. J. Davey: @prwjdavey

Table of Contents

NMR Characterization S3
MS Characterization S39
Infrared Spectroscopy S41
UV-Vis Spectroscopy S42
Radiolabeling and Radiolabeling Method Development S44
DFT Calculations – Cartesian Coordinates & Electrostatic Potential Maps S49
References S60

NMR Characterization

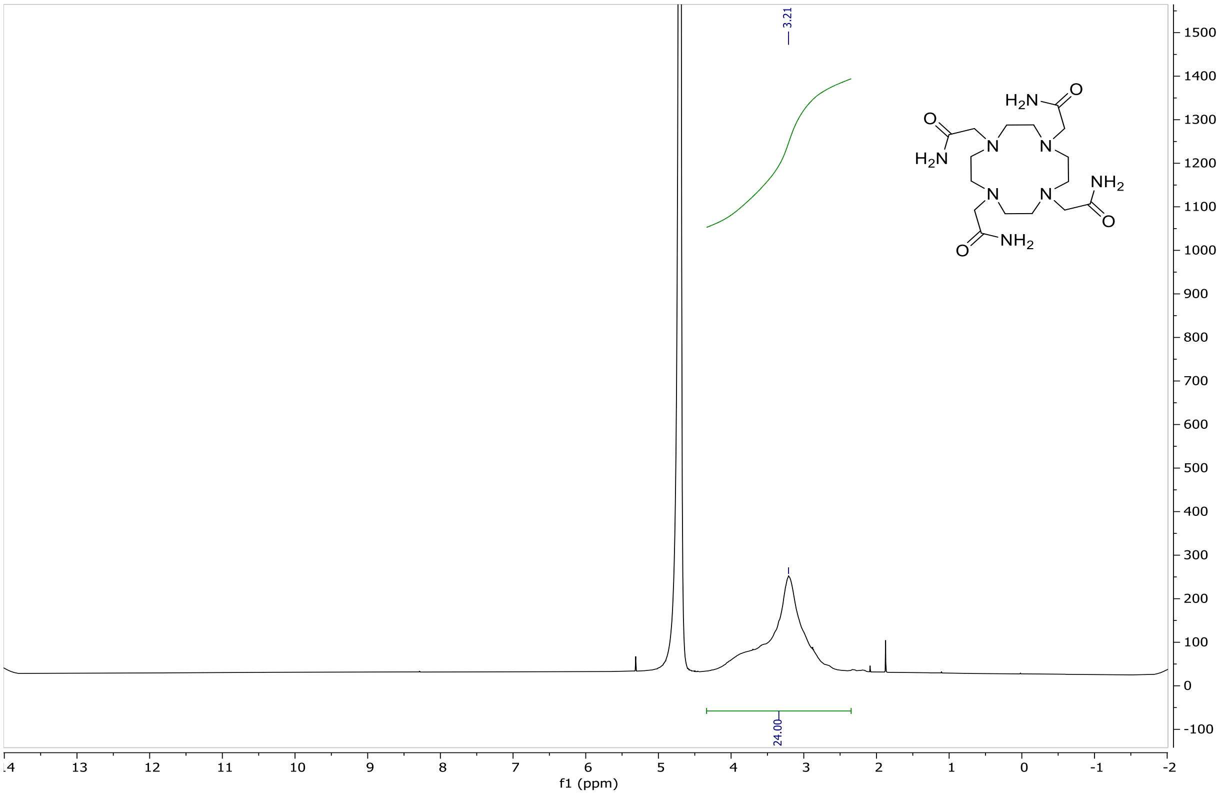

Figure S1. ^{1}H NMR (600 MHz, D_2O, 25 ºC): TCMC (pD 5.4).

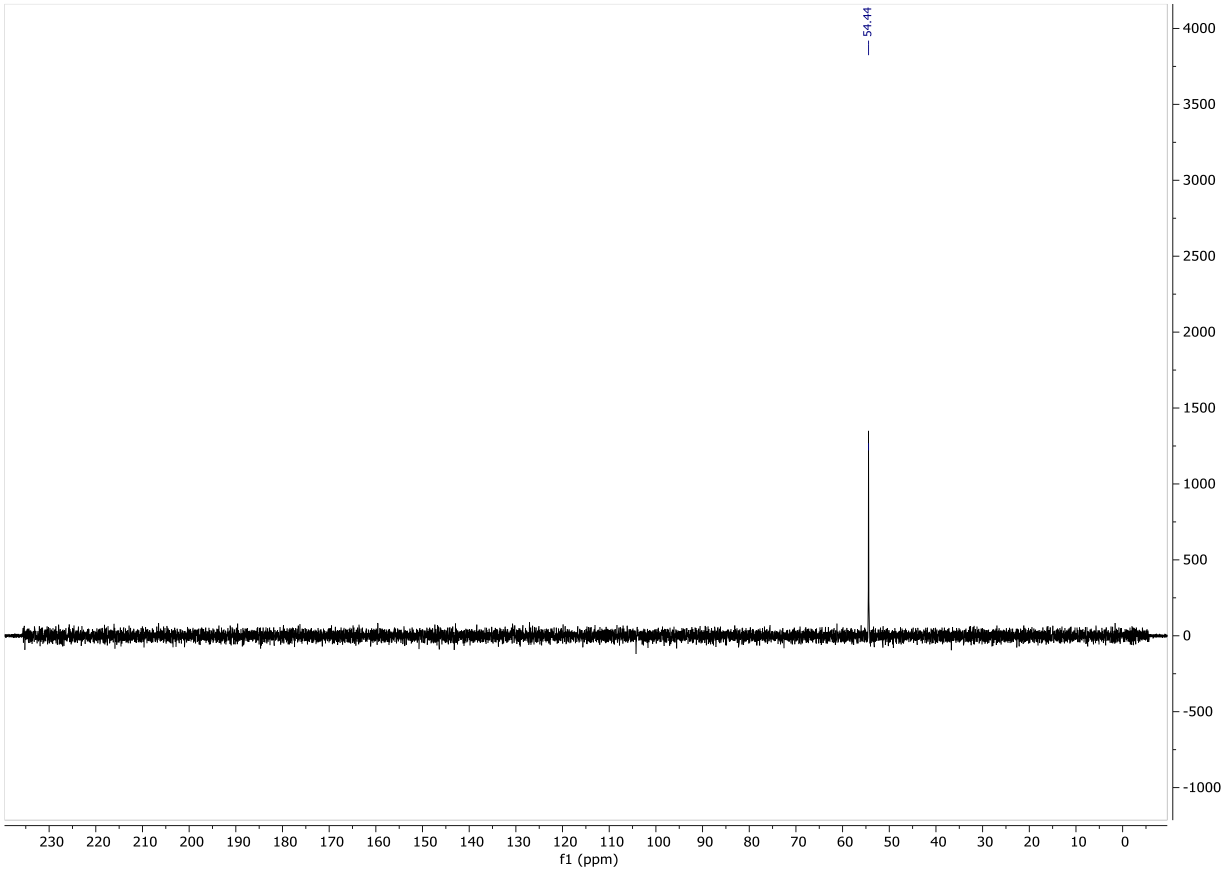

Figure S2. ^{13}C NMR (151 MHz, D_2O, 25 ºC): TCMC (pD 5.4).

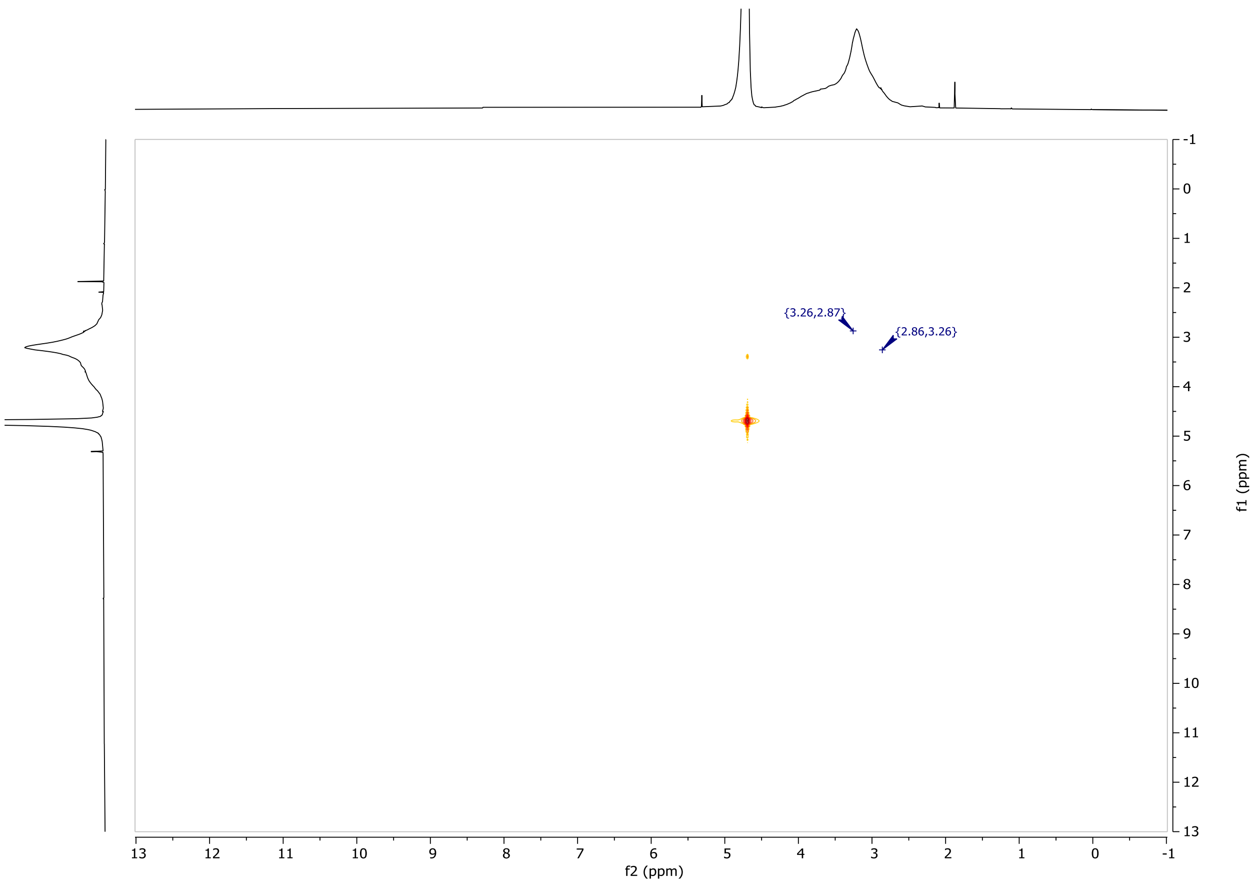

Figure S3. ^1H-^1H COSY NMR (600 MHz, D_2O, 25 °C): TCMC (pD 5.4).

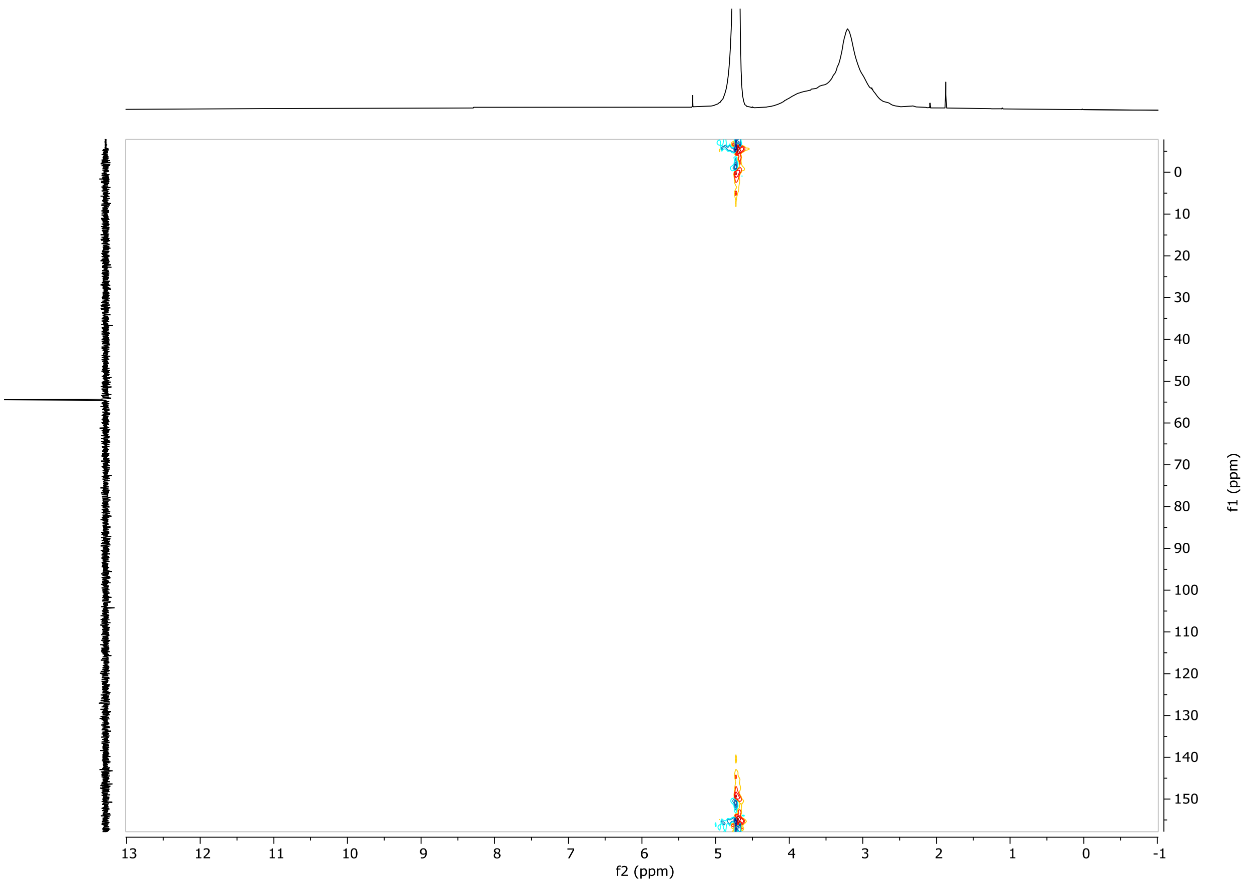

Figure S4. ^{1}H-^{13}C HSQC NMR (600 MHz, D_2O, 25 ºC): TCMC (pD 5.4).

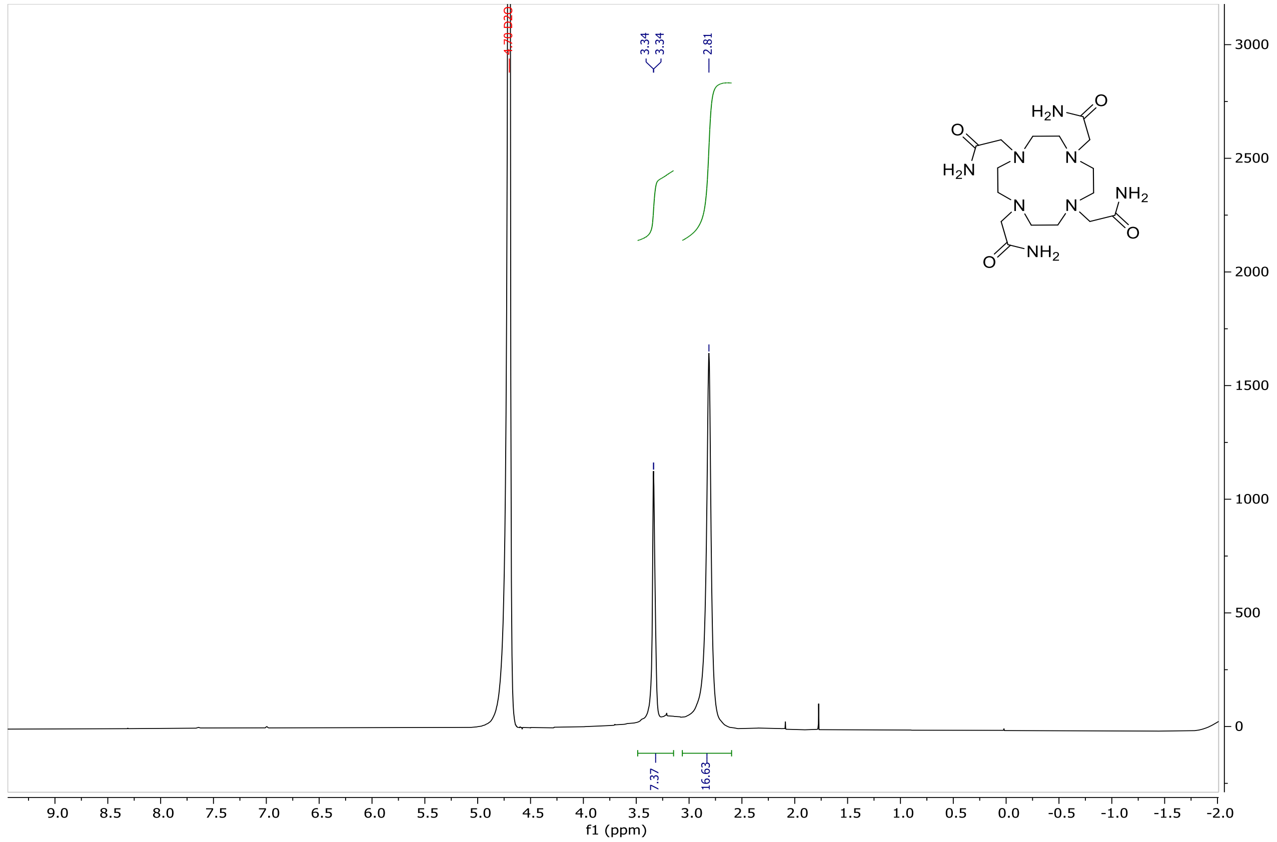

Figure S5. ^{1}H NMR (600 MHz, D_2O, 25 °C): TCMC (pD 7.4).

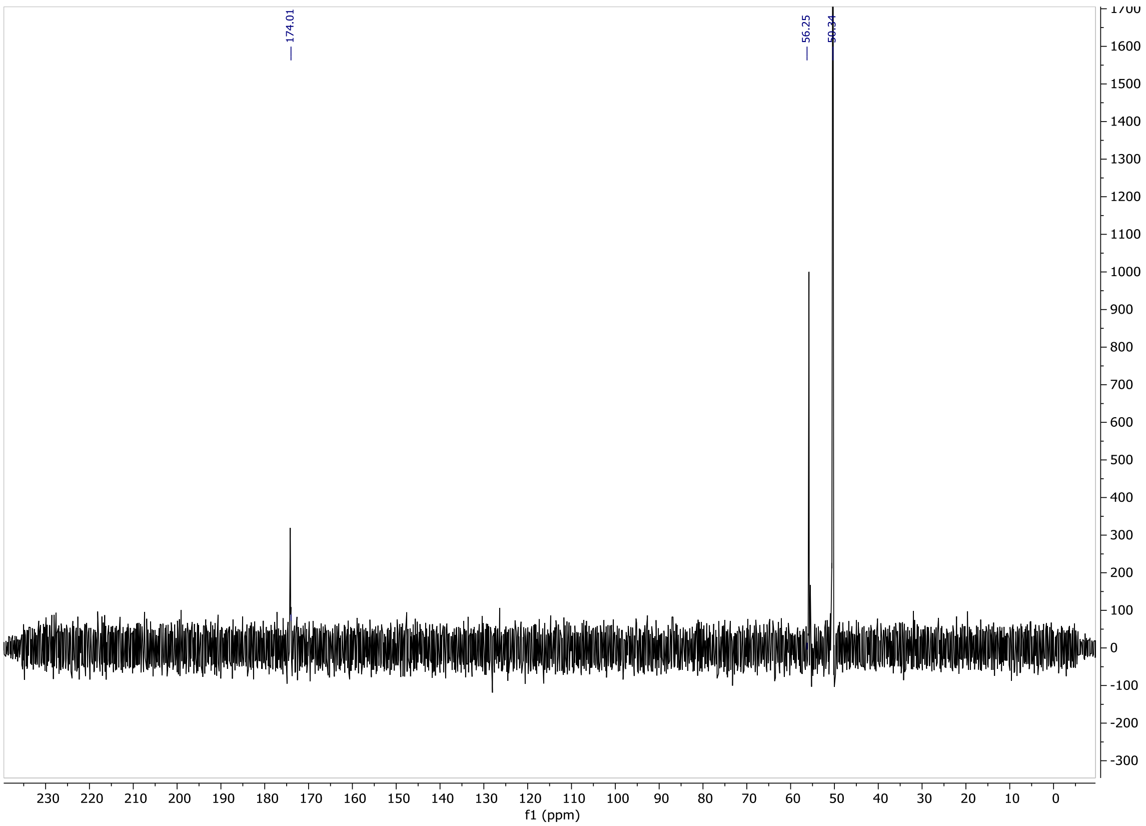

Figure S6. ^{13}C NMR (151 MHz, D_2O, 25 ºC): TCMC (pD 7.4).

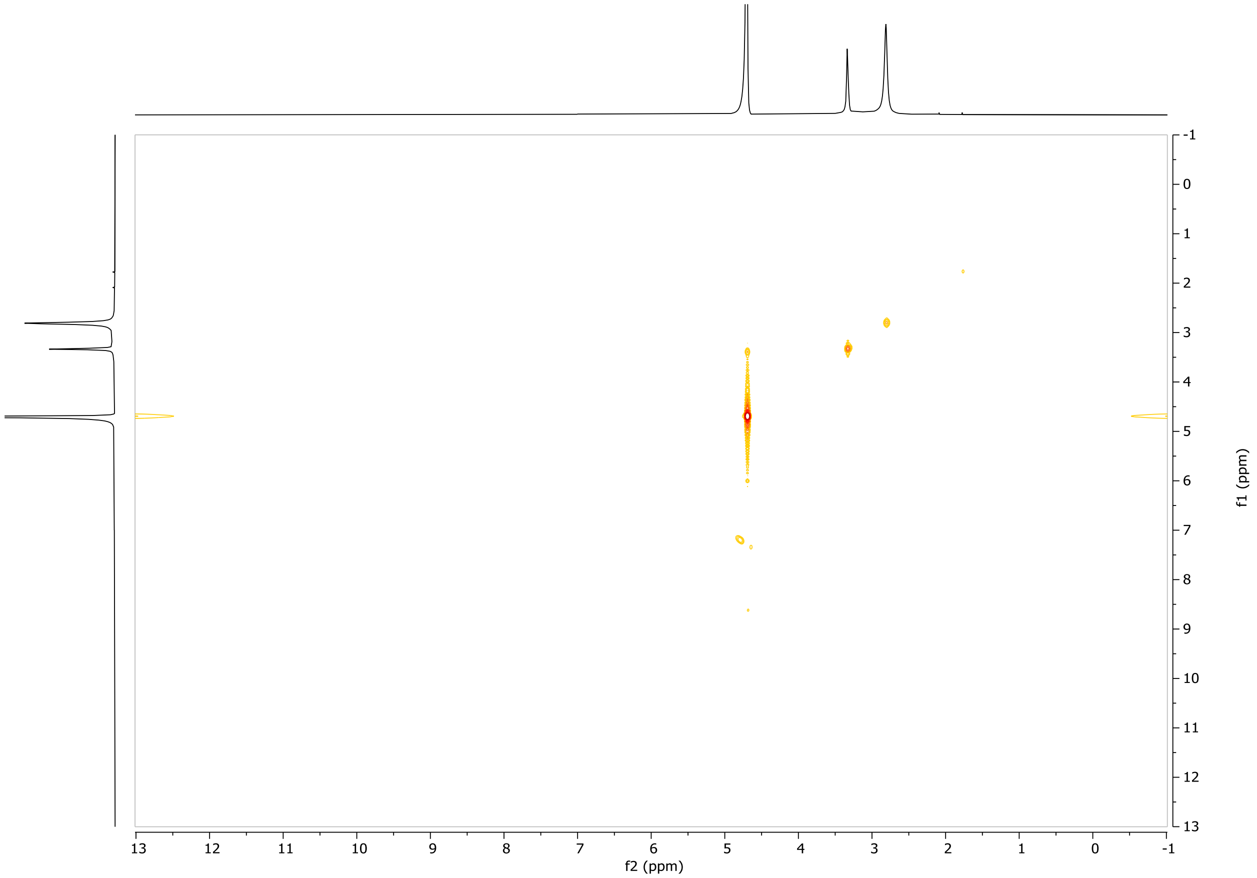

Figure S7. ^1H-^1H COSY NMR (600 MHz, D_2O, 25 °C): TCMC (pD 7.4).

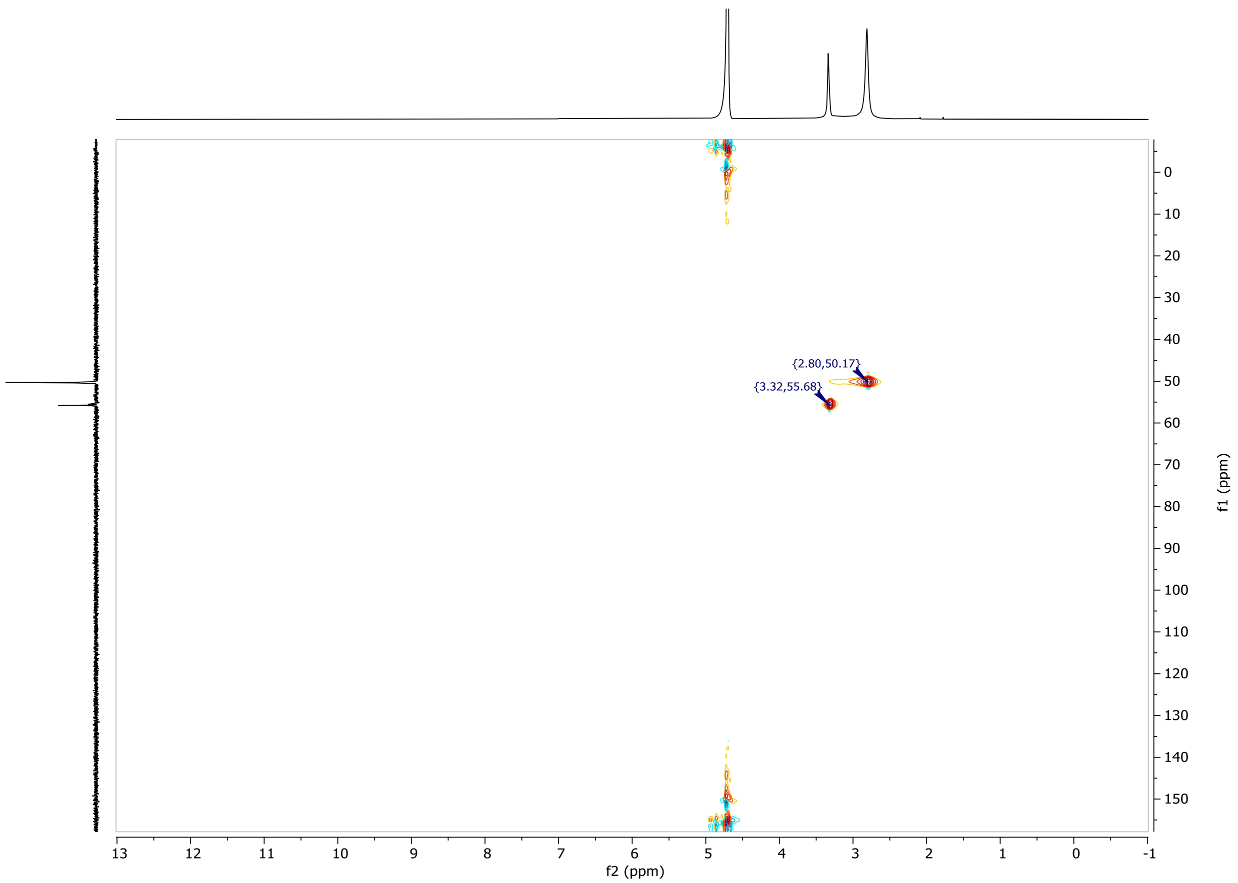

Figure S8. ^{1}H-^{13}C HSQC NMR (600 MHz, D_2O, 25 ºC): TCMC (pD 7.4).

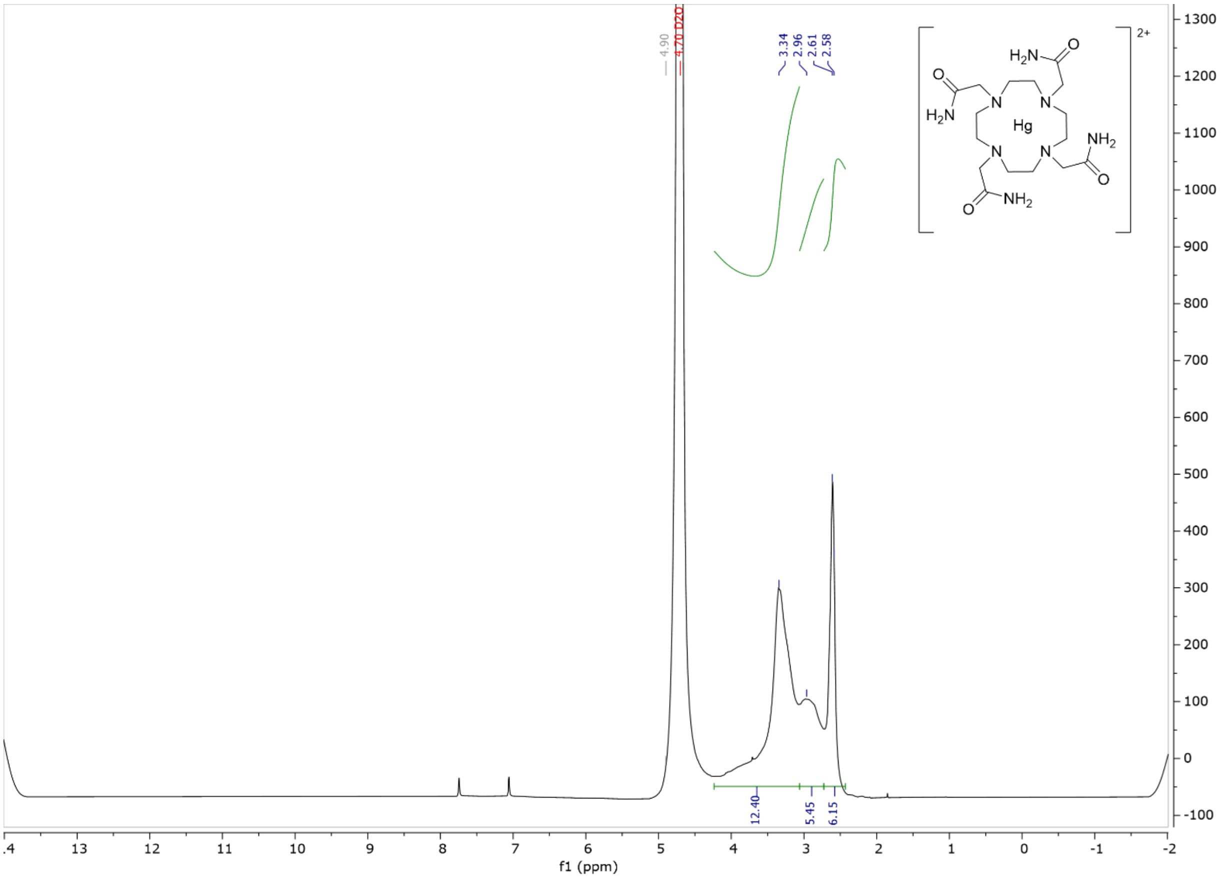

Figure S9. ^{1}H NMR (600 MHz, D_2O, 25 ºC): [^{nat}Hg][Hg(TCMC)]$^{2+}$ (pD 5.4).

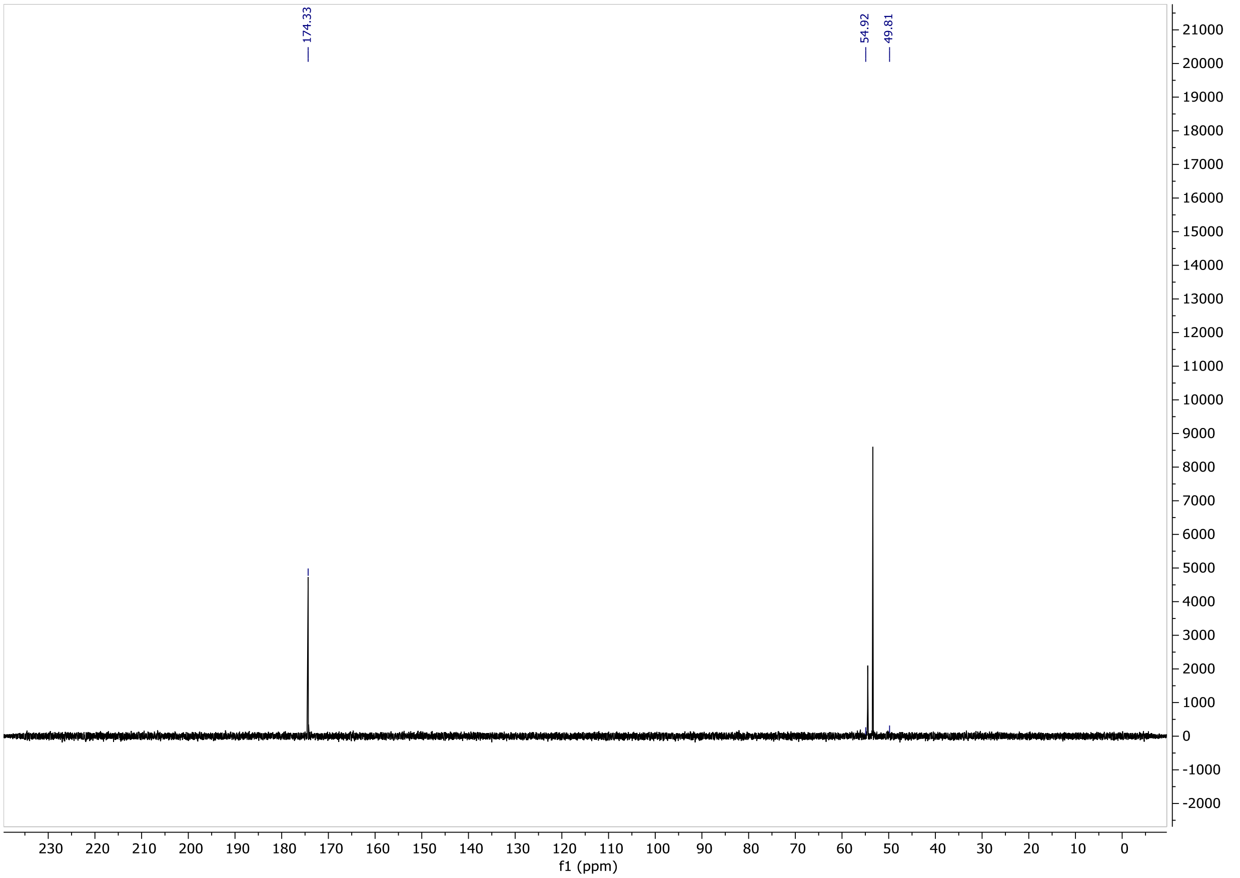

Figure S10. ^{13}C NMR (151 MHz, D_2O, 25 ºC): [^{nat}Hg][Hg(TCMC)]$^{2+}$ (pD 5.4).

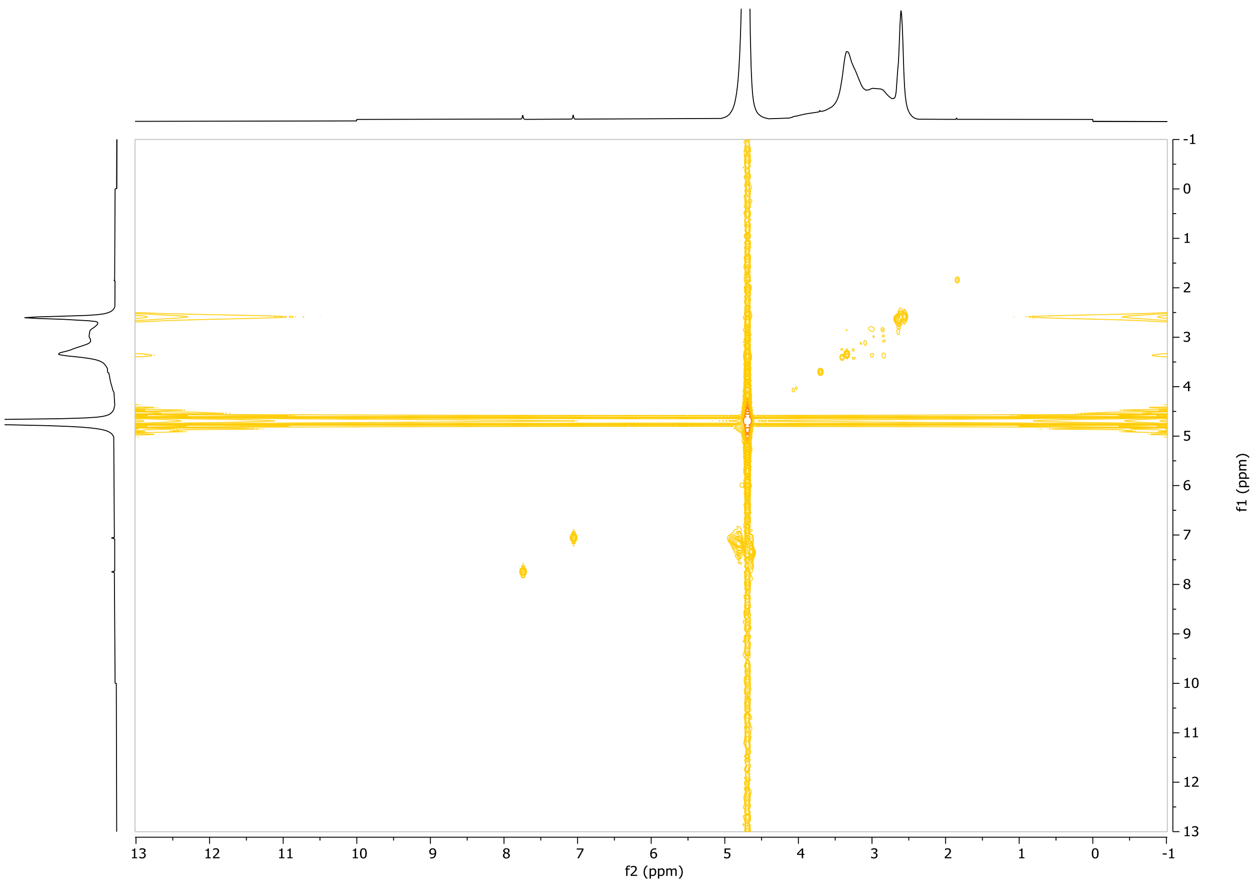

Figure S11. ^{1}H-^{1}H COSY NMR (600 MHz, D_2O, 25 ºC): [^{nat}Hg][Hg(TCMC)]$^{2+}$ (pD 5.4).

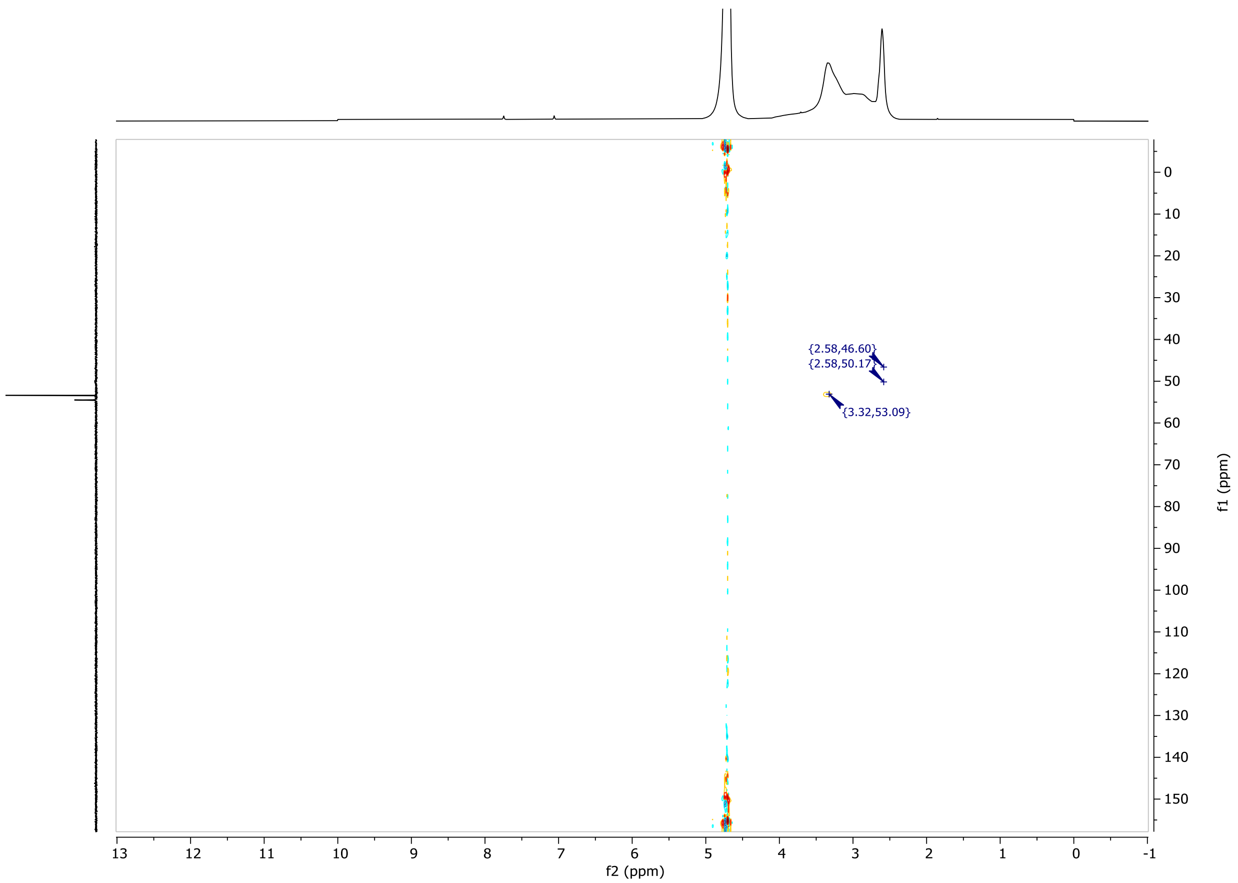

Figure S12. ^{1}H-^{13}C HSQC NMR (600 MHz, D_2O, 25 ºC): [^{nat}Hg][Hg(TCMC)]$^{2+}$ (pD 5.4).

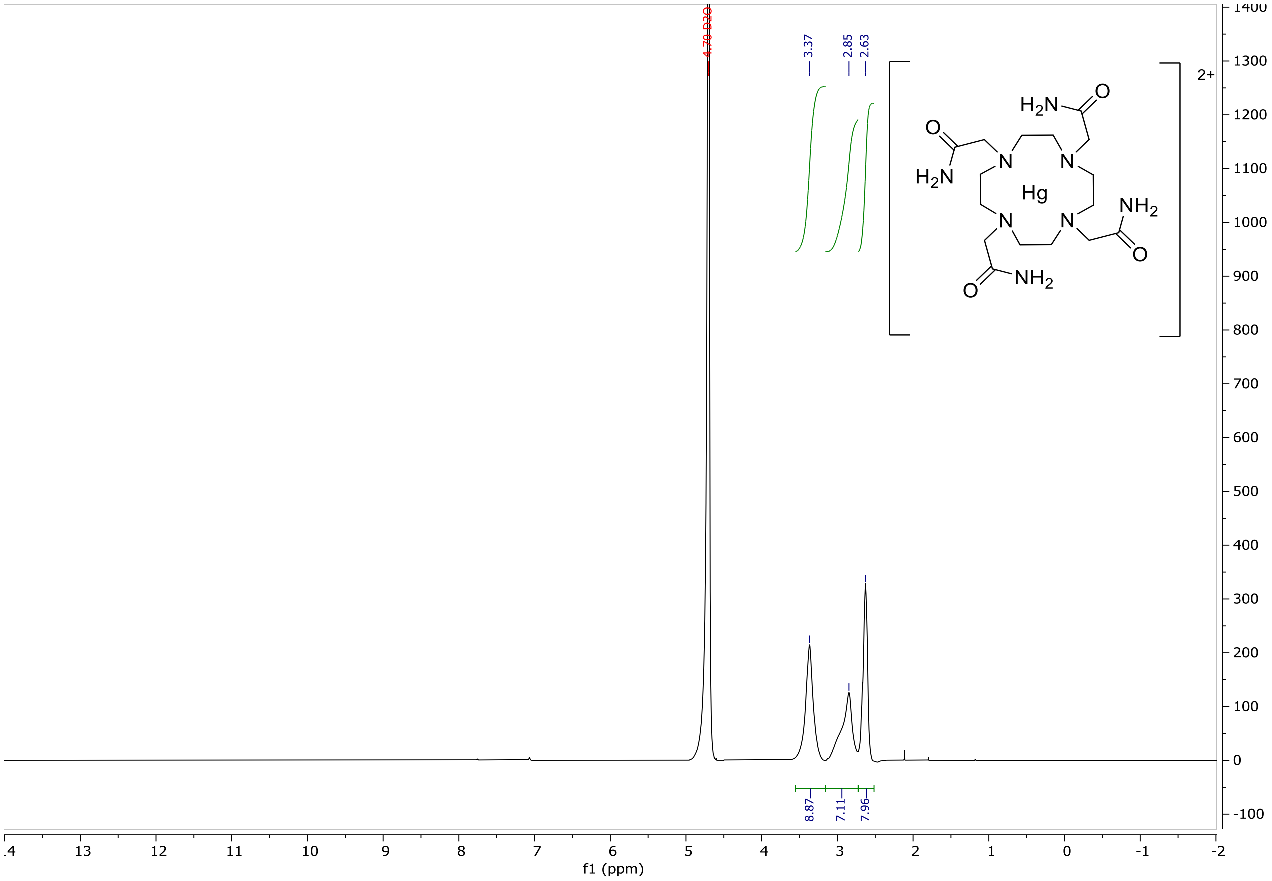

Figure S13. ^{1}H NMR (600 MHz, D_2O, 25 ºC): $[^{nat}Hg][Hg(TCMC)]^{2+}$ (pD 7.4).

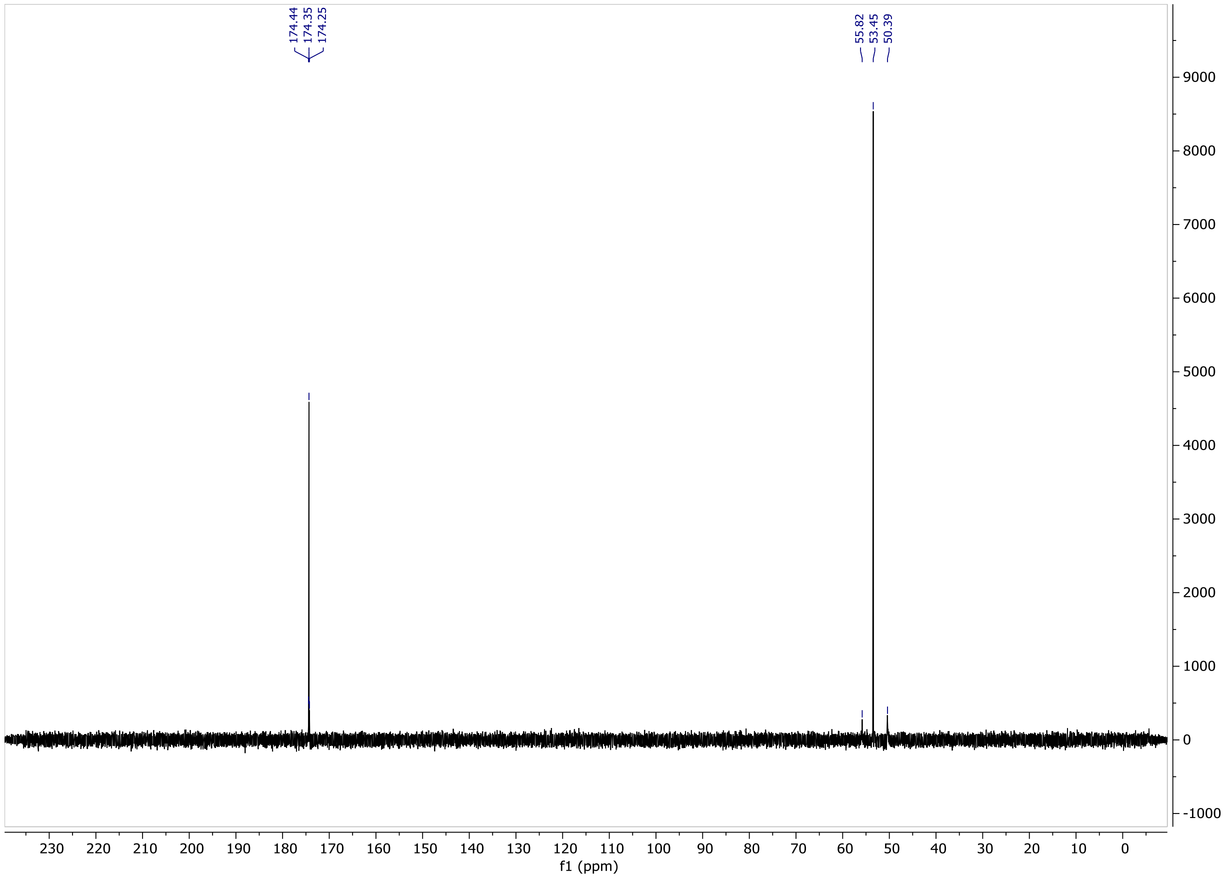

Figure S14. ^{13}C NMR (151 MHz, D_2O, 25 ºC): $[^{nat}Hg][Hg(TCMC)]^{2+}$ (pD 7.4).

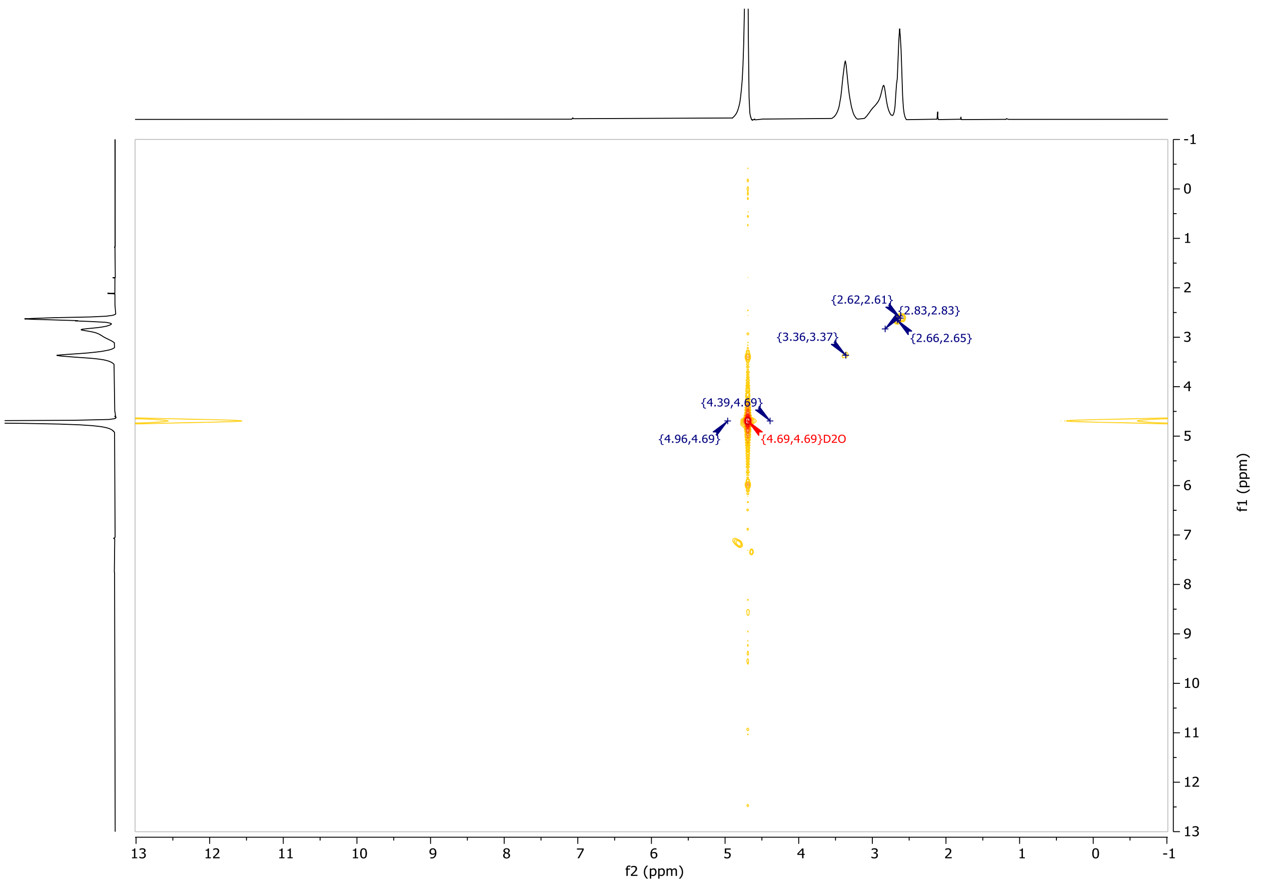

Figure S15. ^{1}H-^{1}H COSY NMR (600 MHz, D_2O, 25 ºC): [^{nat}Hg][Hg(TCMC)]$^{2+}$ (pD 7.4).

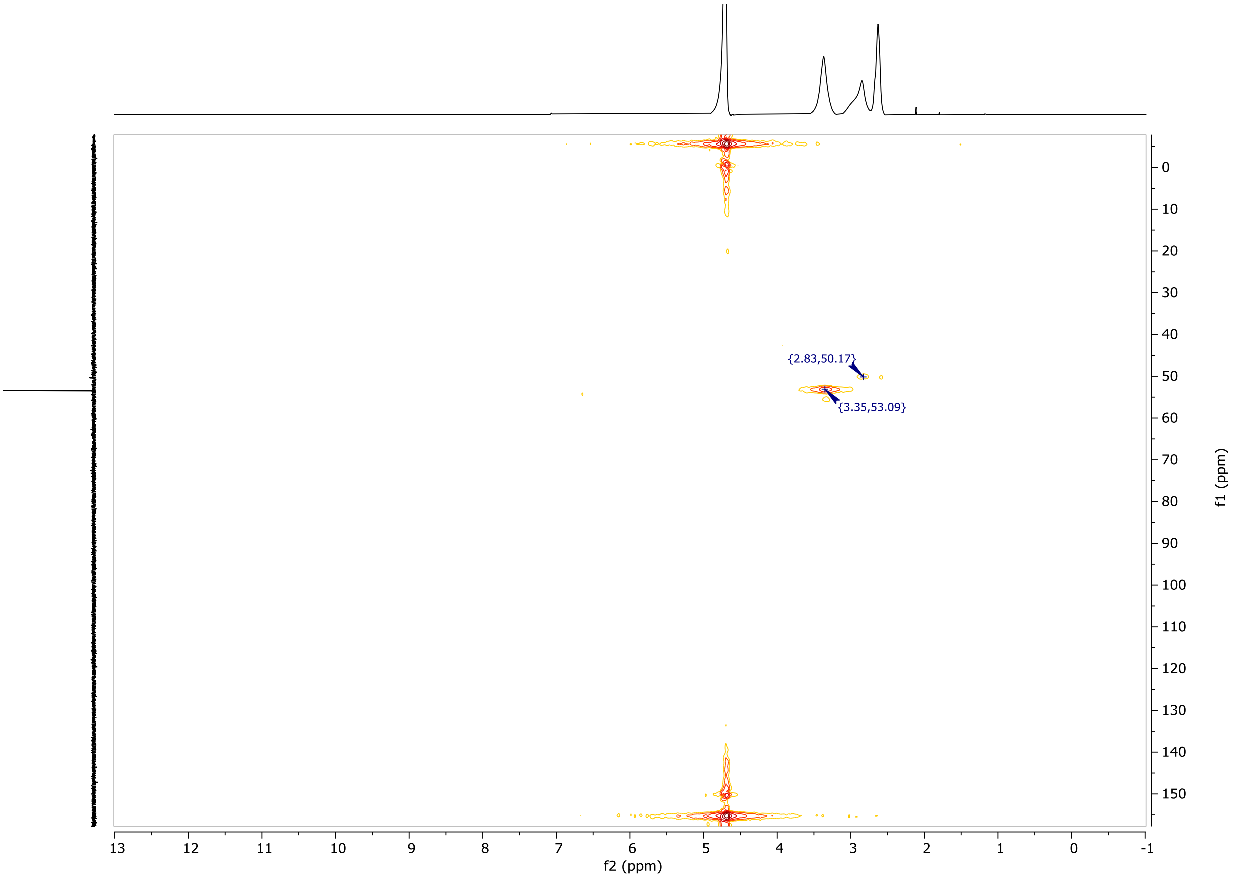

Figure S16. ^{1}H-^{13}C HSQC NMR (600 MHz, D_2O, 25 °C): [^{nat}Hg][Hg(TCMC)]$^{2+}$ (pD 7.4).

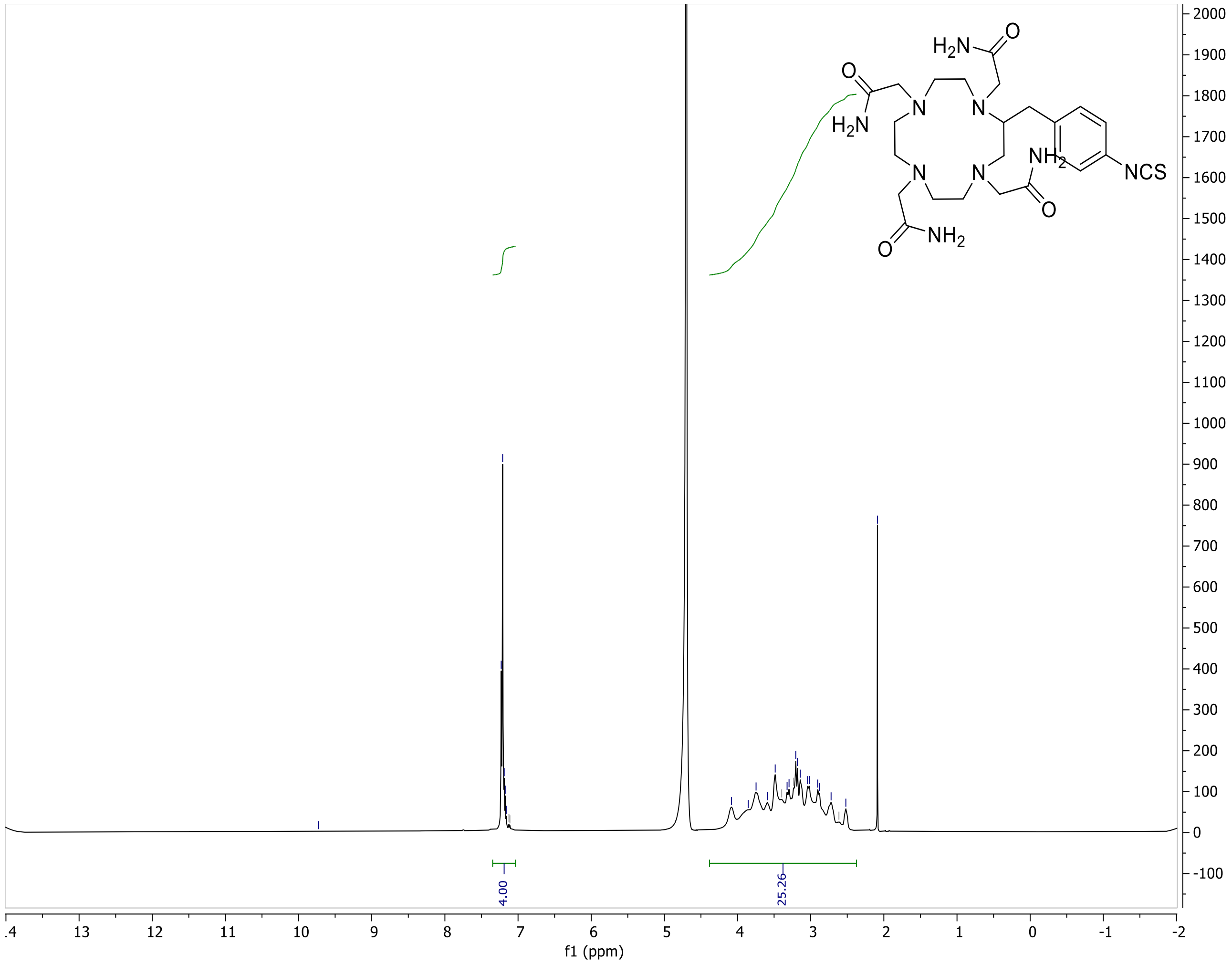

Figure S17. ^{1}H NMR (600 MHz, D_2O, 25 ºC): *p*-SCN-Bn-TCMC (pD 5.4).

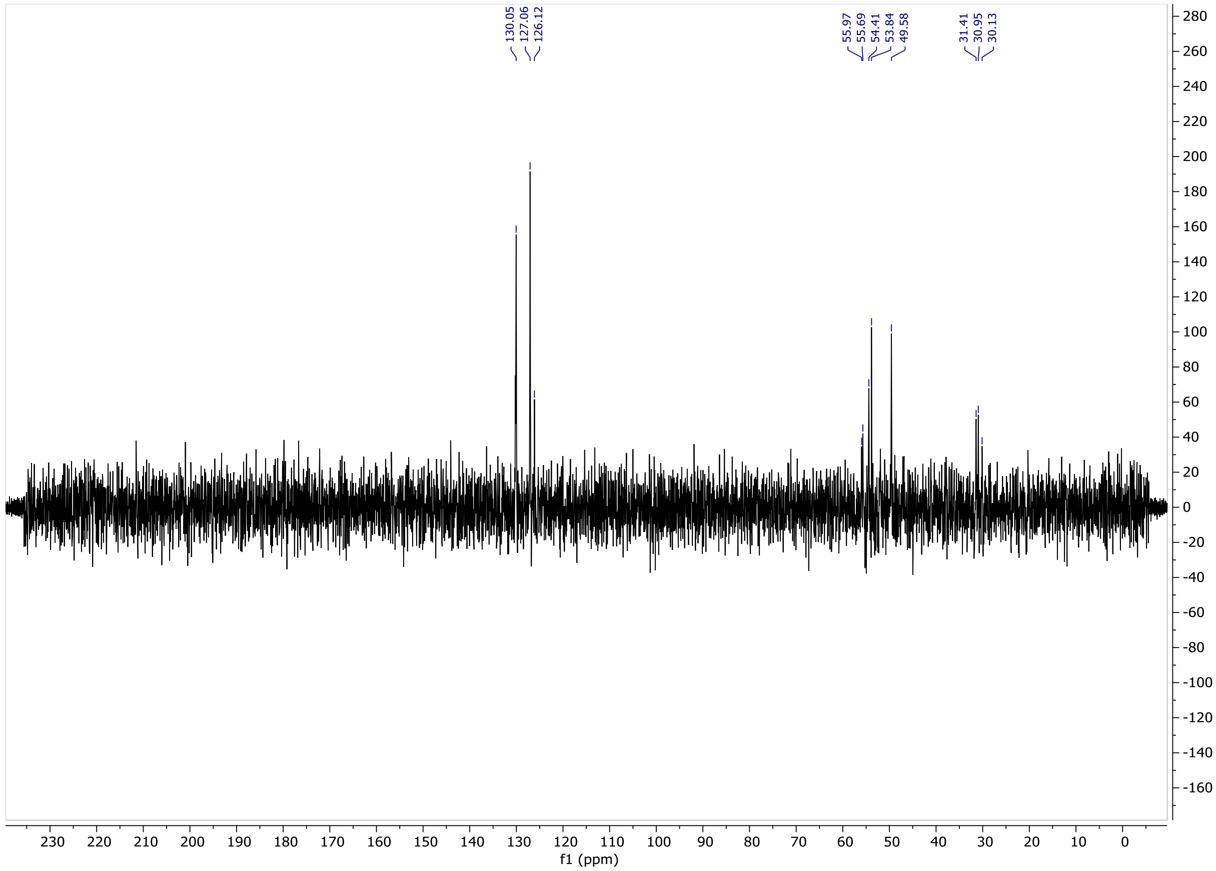

Figure S18. ^{13}C NMR (151 MHz, D_2O, 25 ºC): *p*-SCN-Bn-TCMC (pD 5.4).

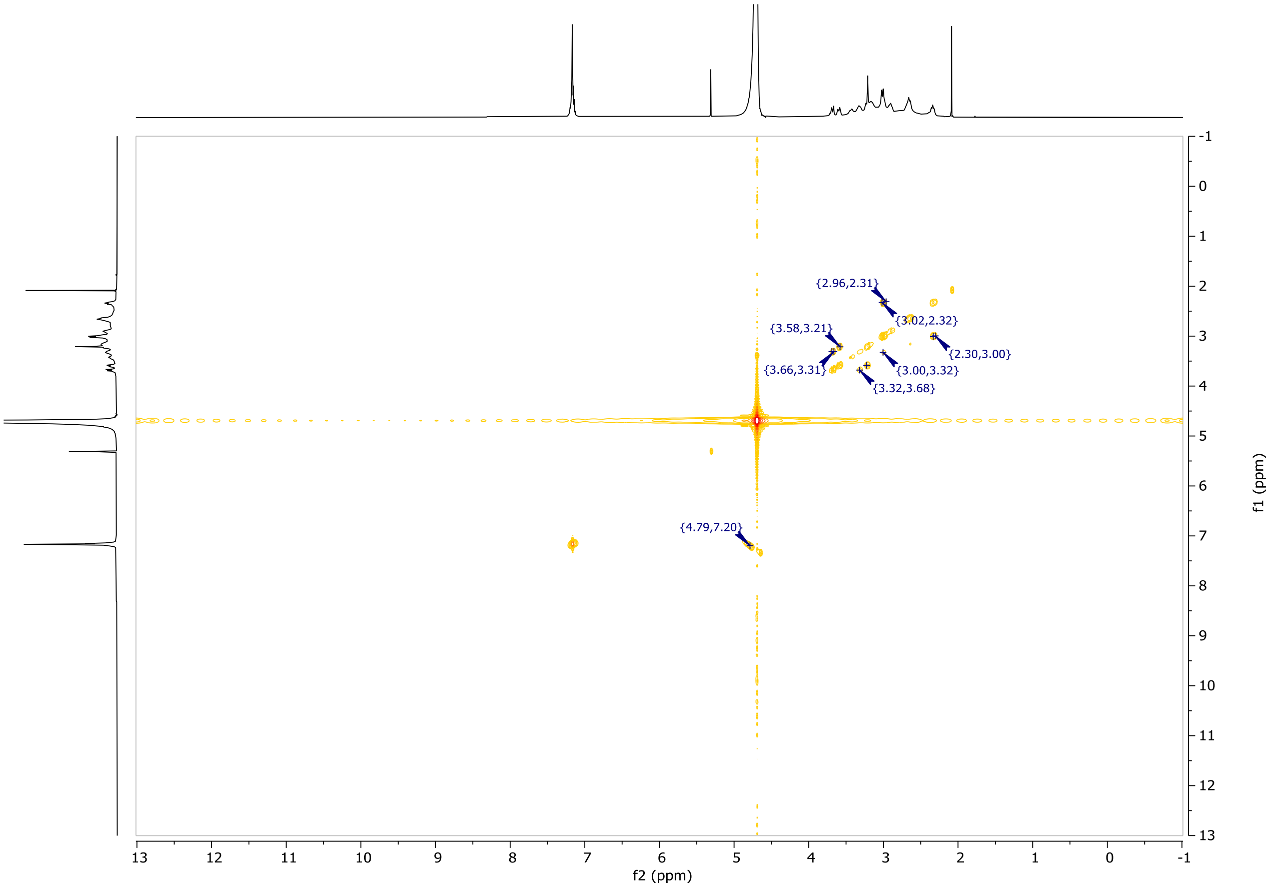

Figure S19. ^{1}H-^{1}H COSY NMR(600 MHz, D_2O, 25 °C): *p*-SCN-Bn-TCMC (pD 5.4).

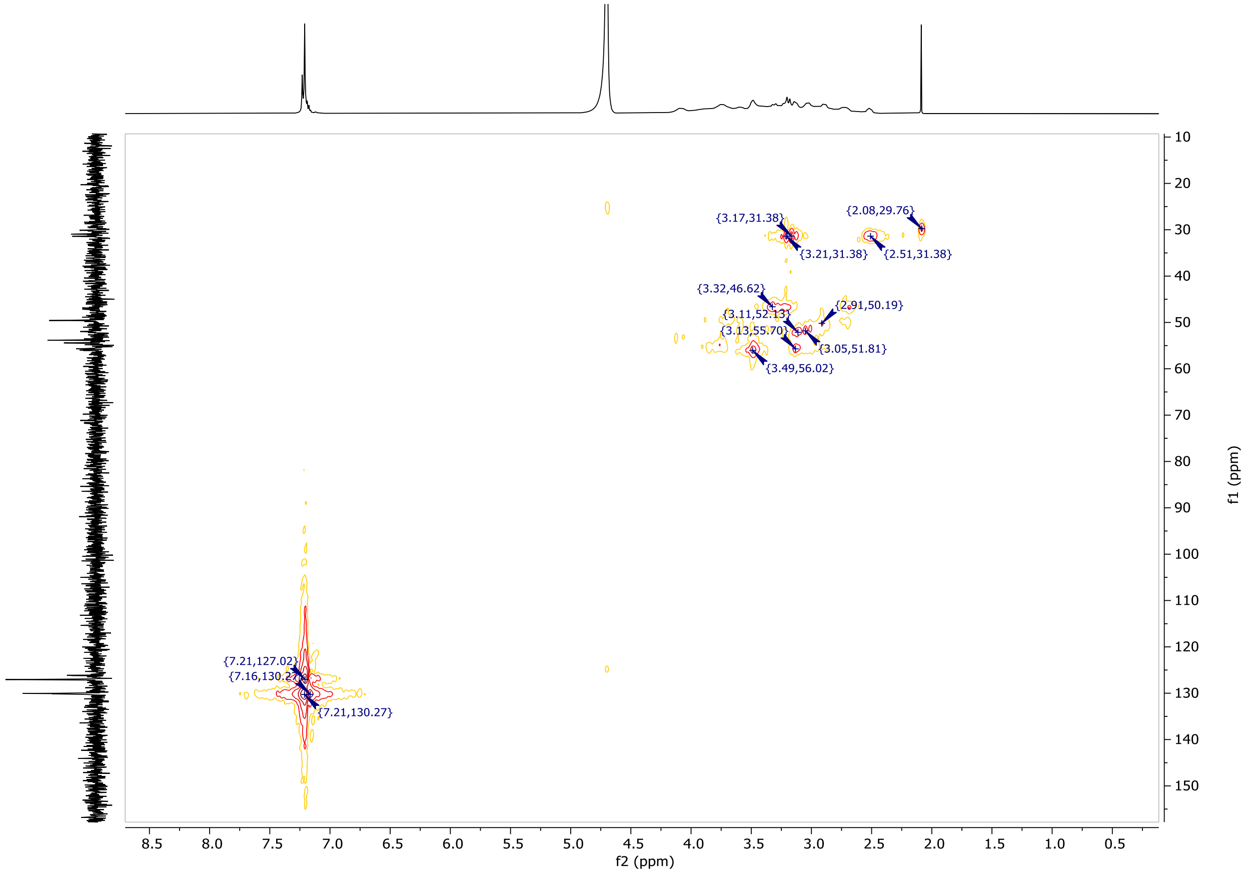

Figure S20. ^{1}H-^{13}C HSQC NMR(600 MHz, D_2O, 25 °C): *p*-SCN-Bn-TCMC (pD 5.4).

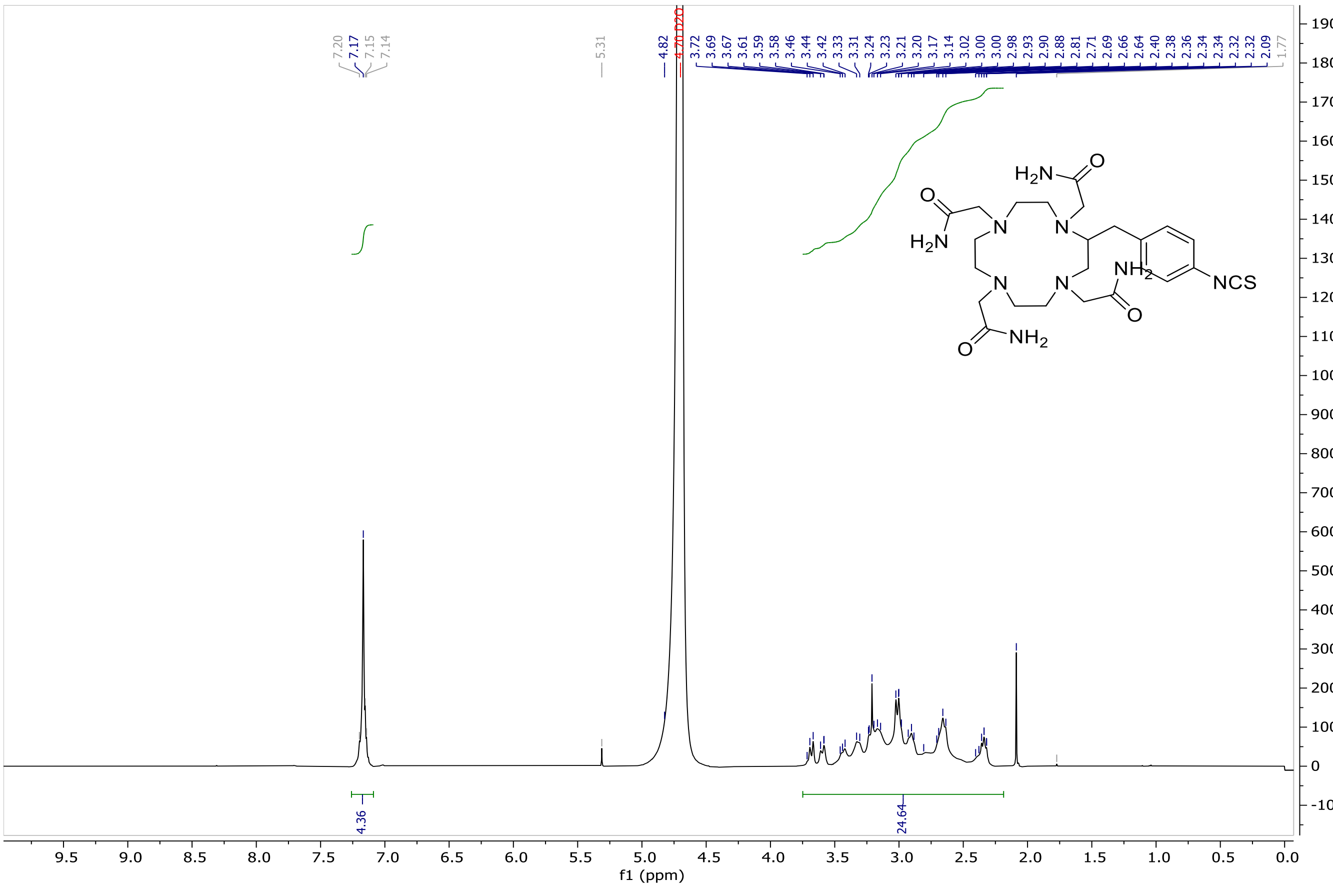

Figure S21. ^{1}H NMR(600 MHz, D_2O, 25 ºC): *p*-SCN-Bn-TCMC (pD 7.4).

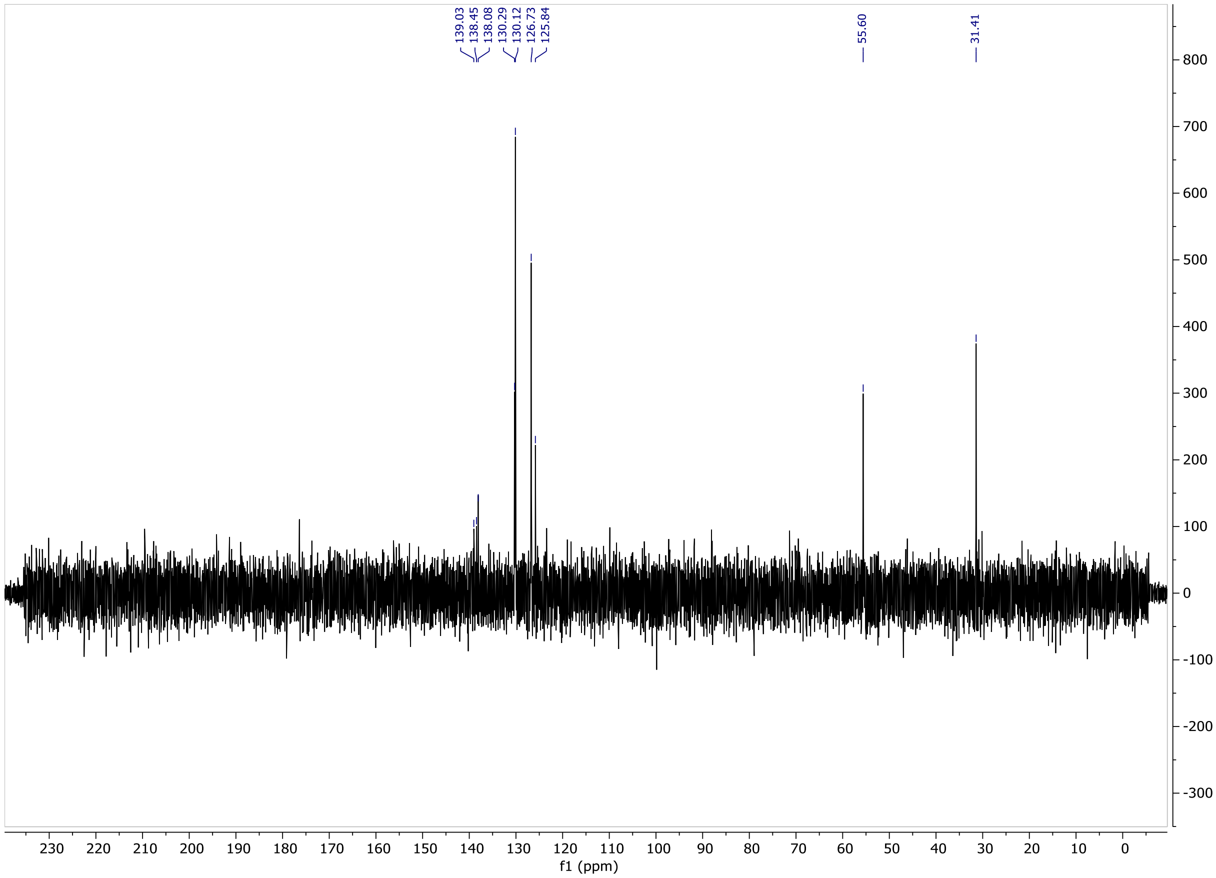

Figure S22. ^{13}C NMR(151 MHz, D_2O, 25 ºC): *p*-SCN-Bn-TCMC (pD 7.4).

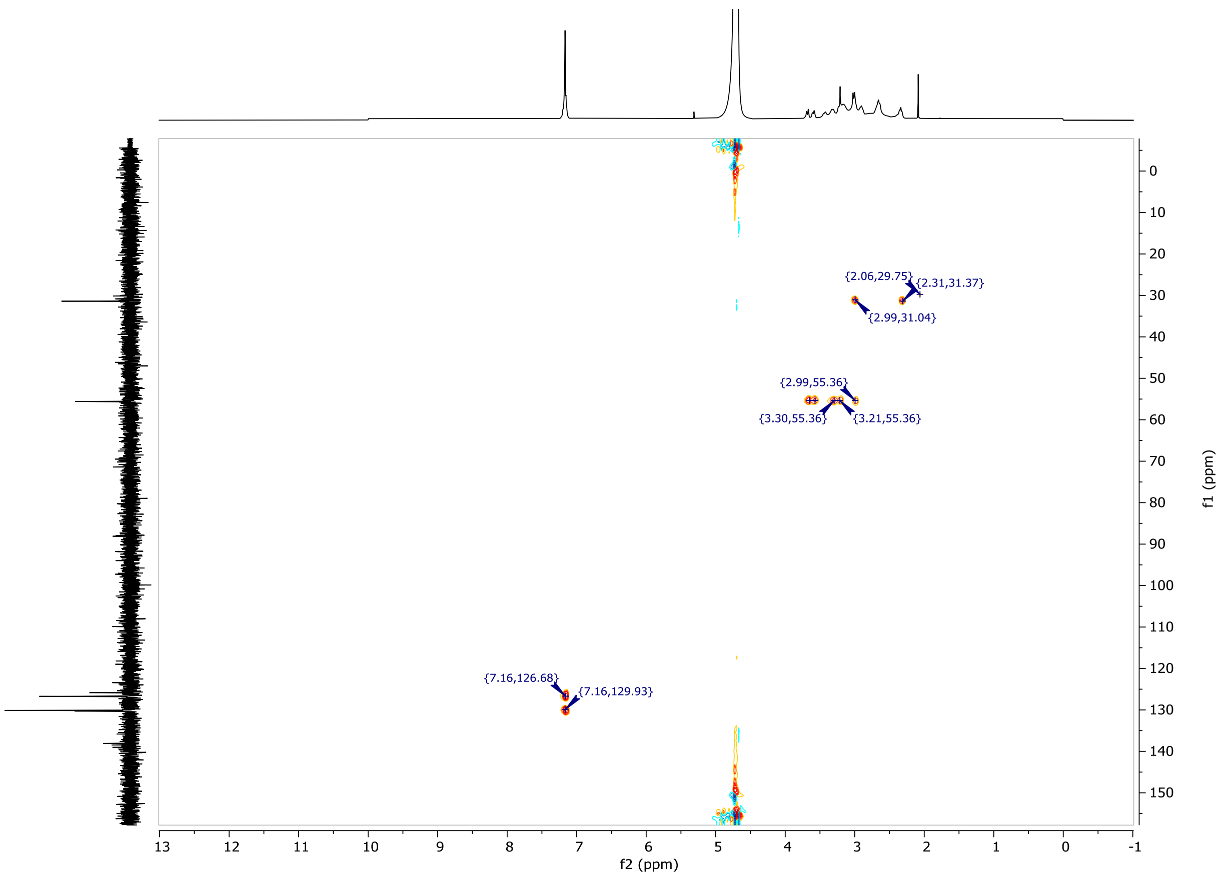

Figure S23. ^{1}H-^{1}H COSY NMR(600 MHz, D_2O, 25 ºC): *p*-SCN-Bn-TCMC (pD 7.4).

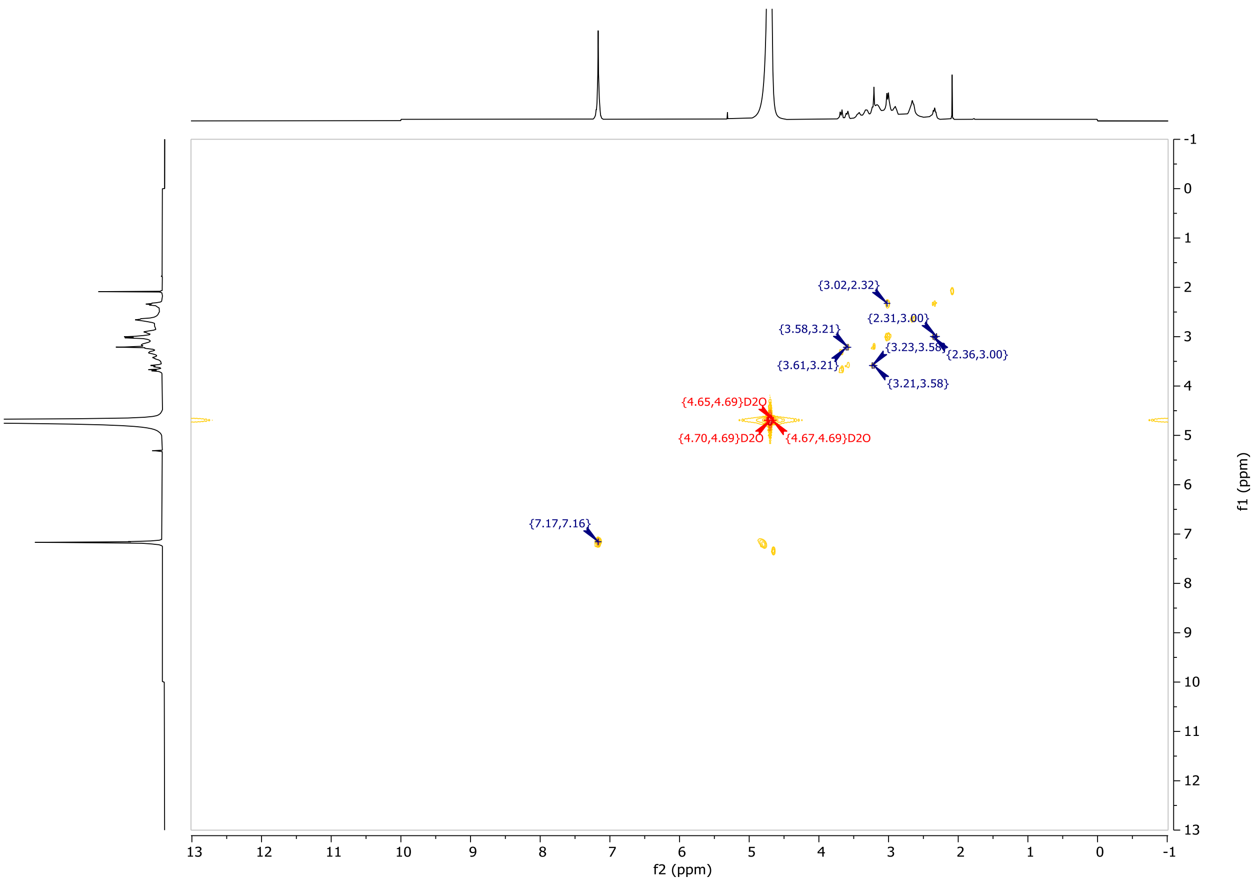

Figure S24. ^{1}H-^{13}C HSQC NMR(600 MHz, D_2O, 25 ºC): *p*-SCN-Bn-TCMC (pD 7.4).

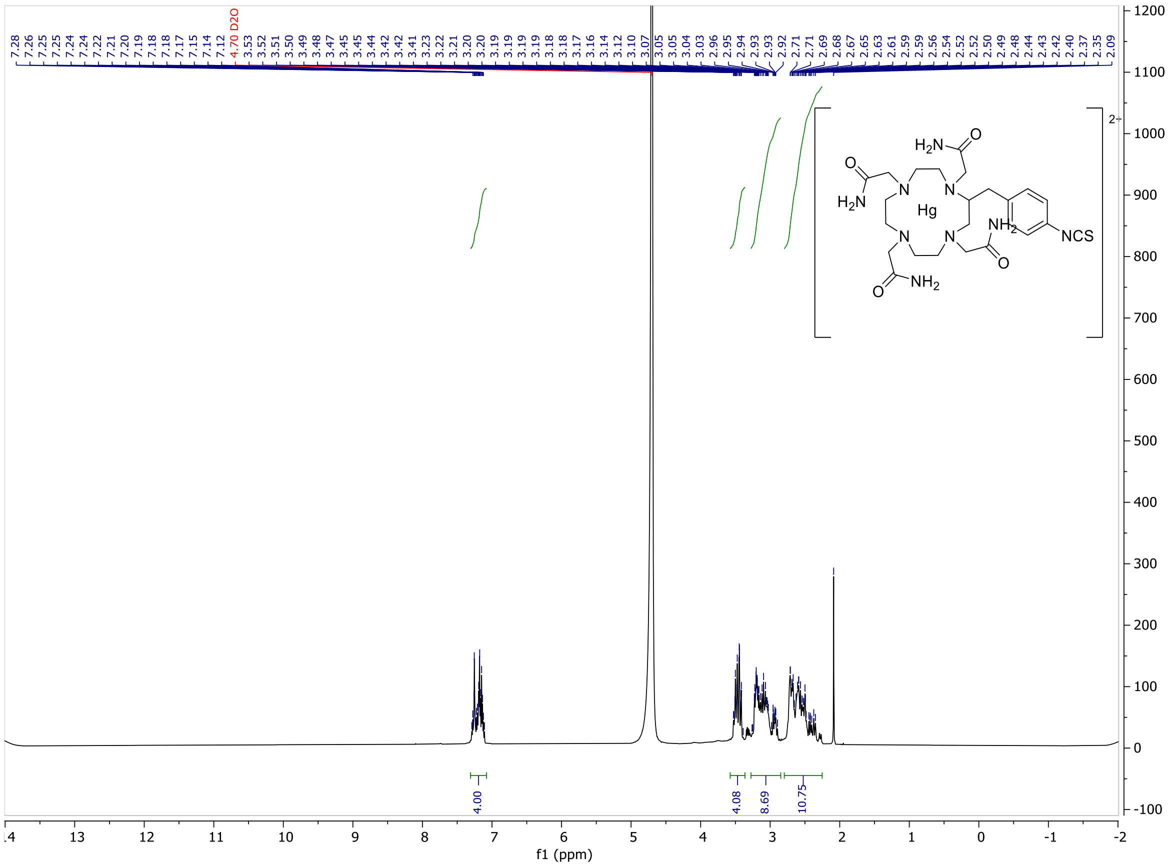

Figure S25. ^{1}H NMR (600 MHz, D_2O, 25 °C): [^{nat}Hg][Hg(*p*-SCN-Bn-TCMC)]$^{2+}$ (pD 5.4).

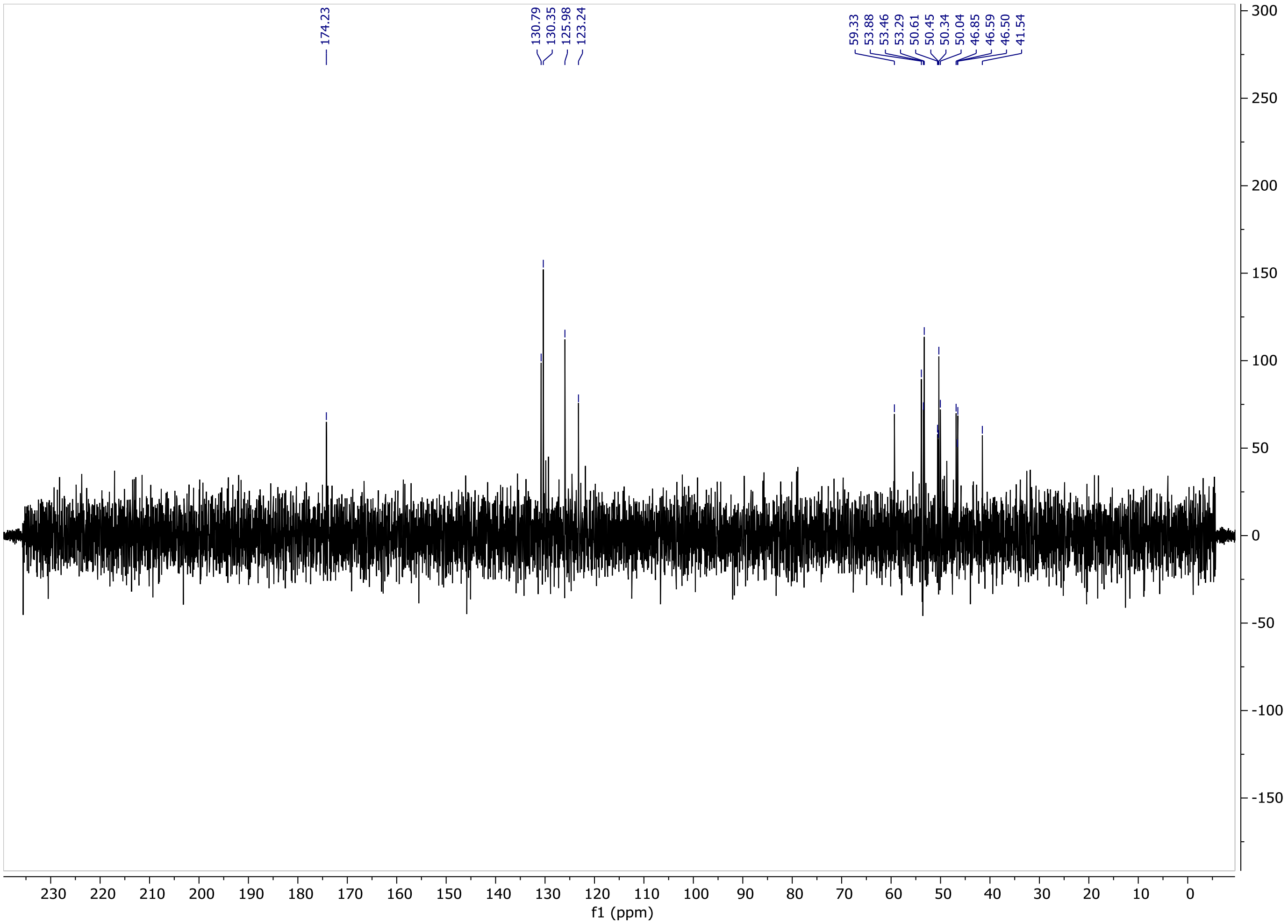

Figure S26. ^{13}C NMR (151MHz, D_2O, 25 °C): [^{nat}Hg][Hg(*p*-SCN-Bn-TCMC)]$^{2+}$ (pD 5.4).

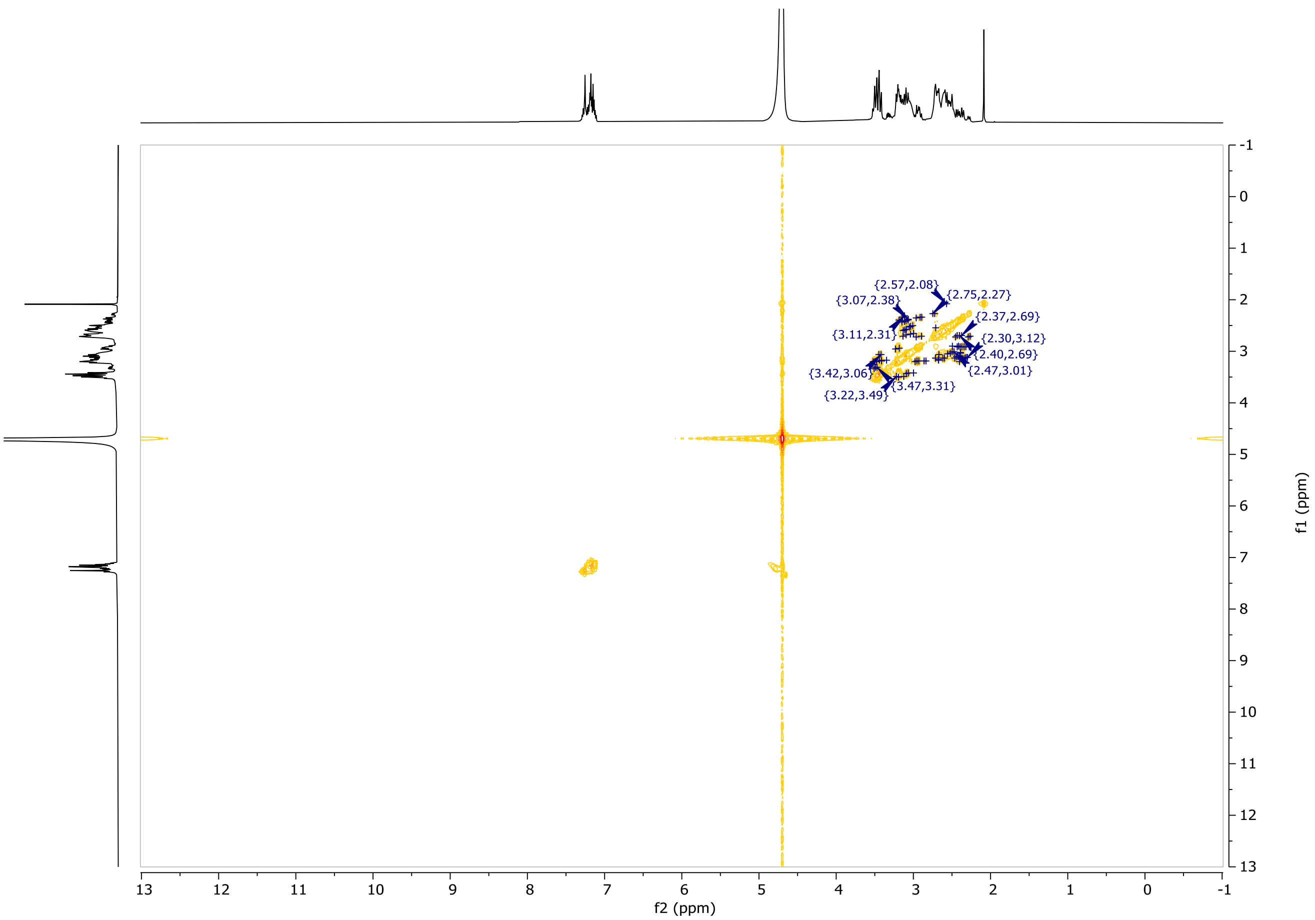

Figure S27. ^{1}H-^{1}H COSY NMR(600 MHz, D_2O, 25 ºC): [^{nat}Hg][Hg(*p*-SCN-Bn-TCMC)]$^{2+}$ (pD 5.4).

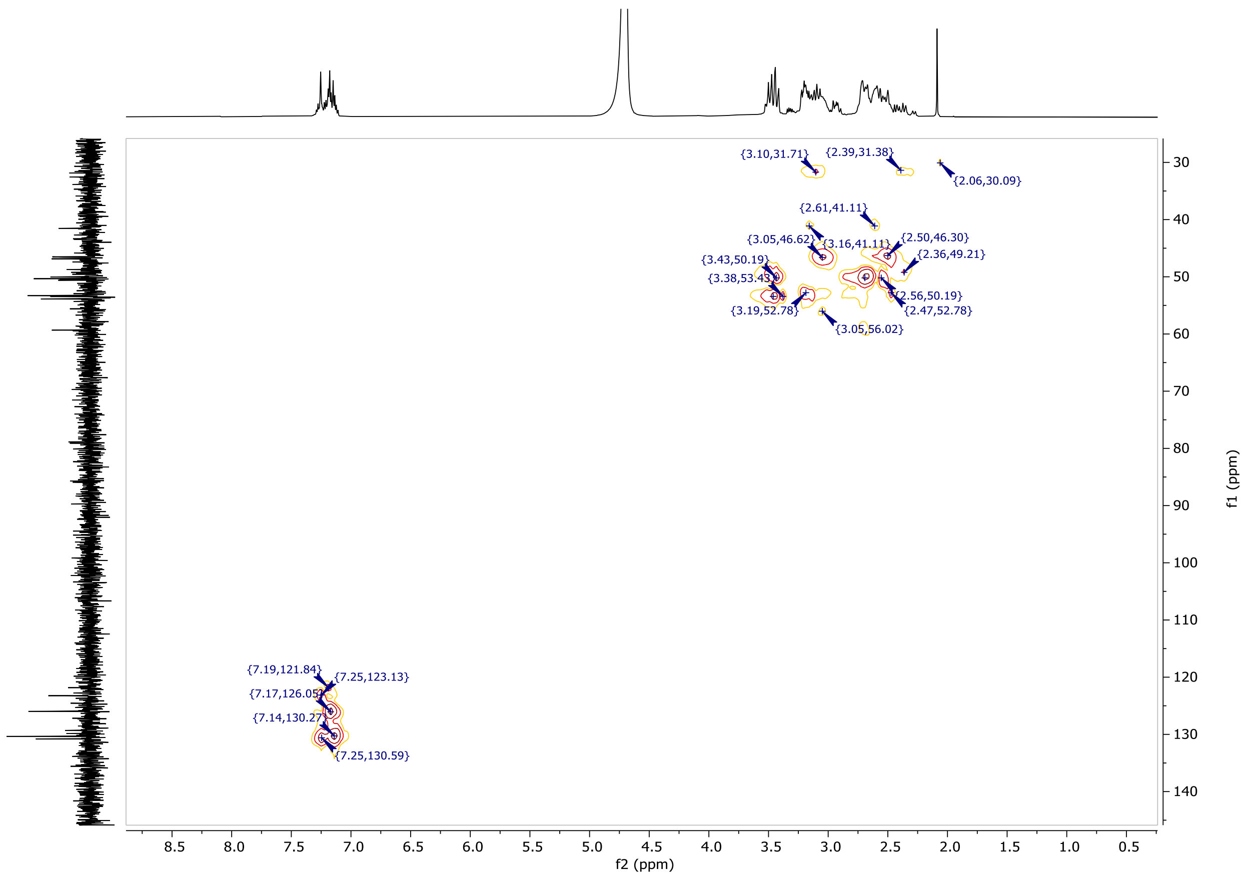

Figure S28. ^{1}H-^{13}C HSQC NMR (600 MHz, D_2O, 25 °C): [^{nat}Hg][Hg(*p*-SCN-Bn-TCMC)]$^{2+}$ (pD 5.4).

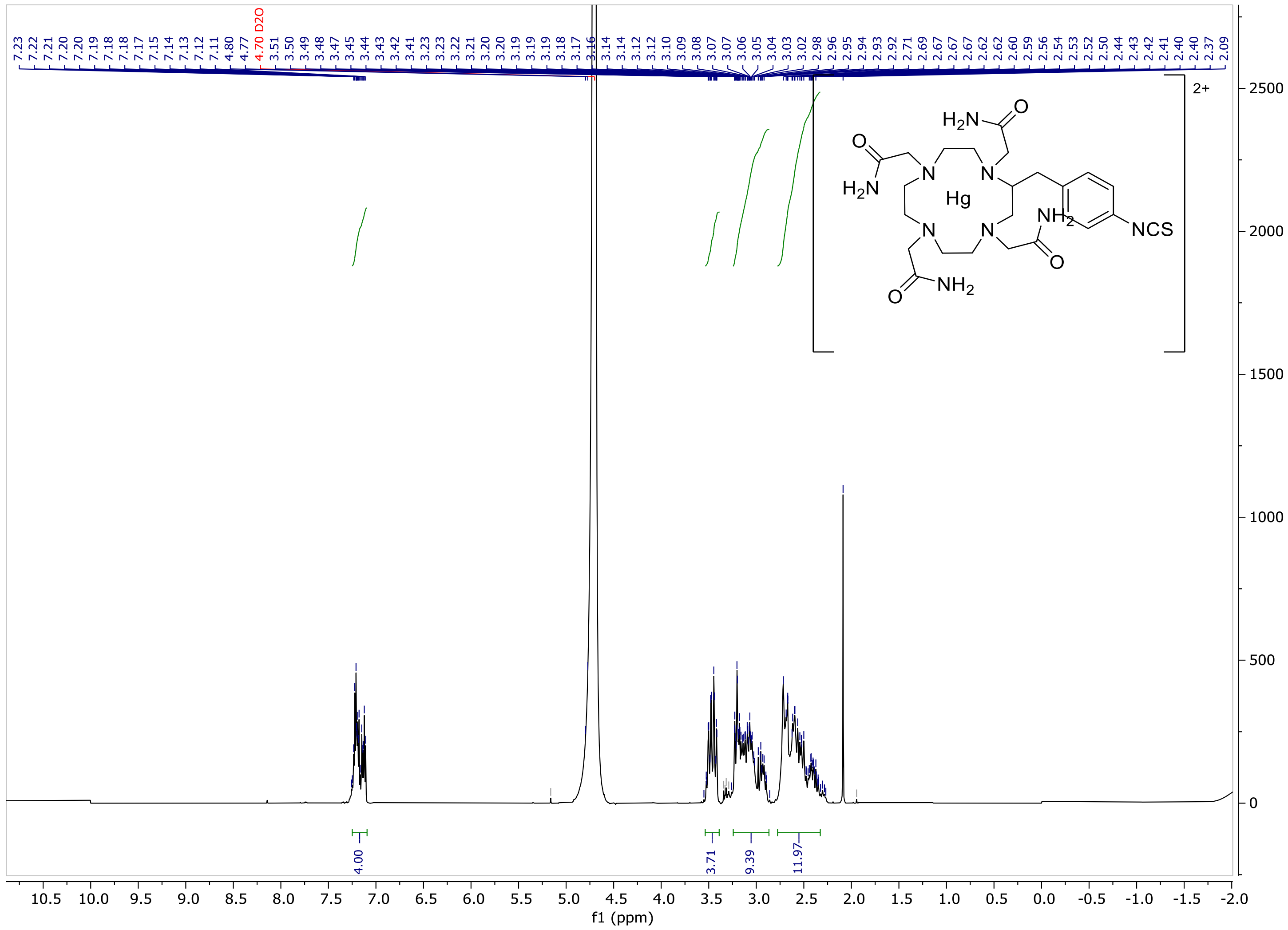

Figure S29. ^{1}H NMR (600 MHz, D_2O, 25 ºC): [^{nat}Hg][Hg(*p*-SCN-Bn-TCMC)]$^{2+}$ (pD 7.4).

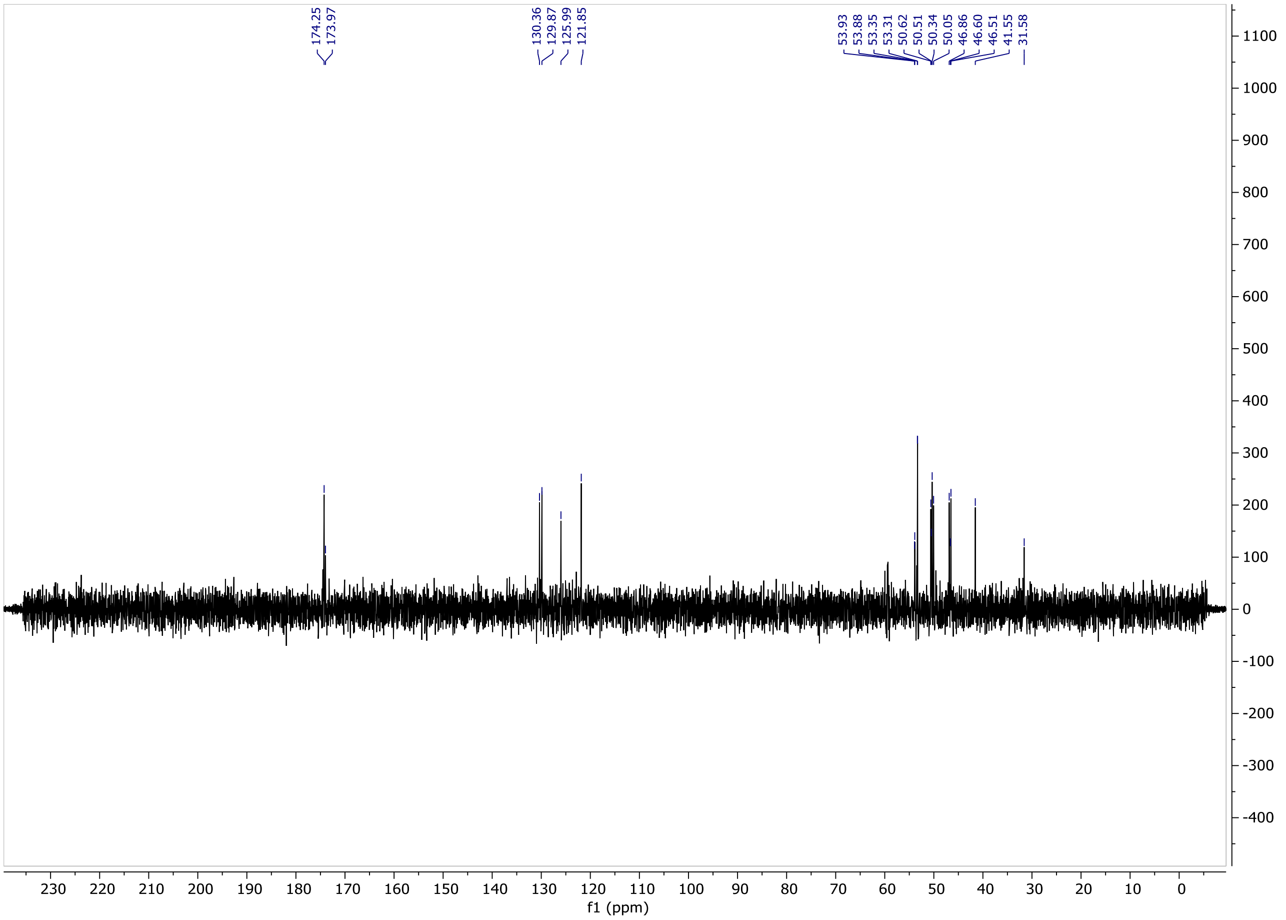

Figure S30. ^{13}C NMR (151 MHz, D_2O, 25 °C): [^{nat}Hg][Hg(*p*-SCN-Bn-TCMC)]$^{2+}$ (pD 7.4).

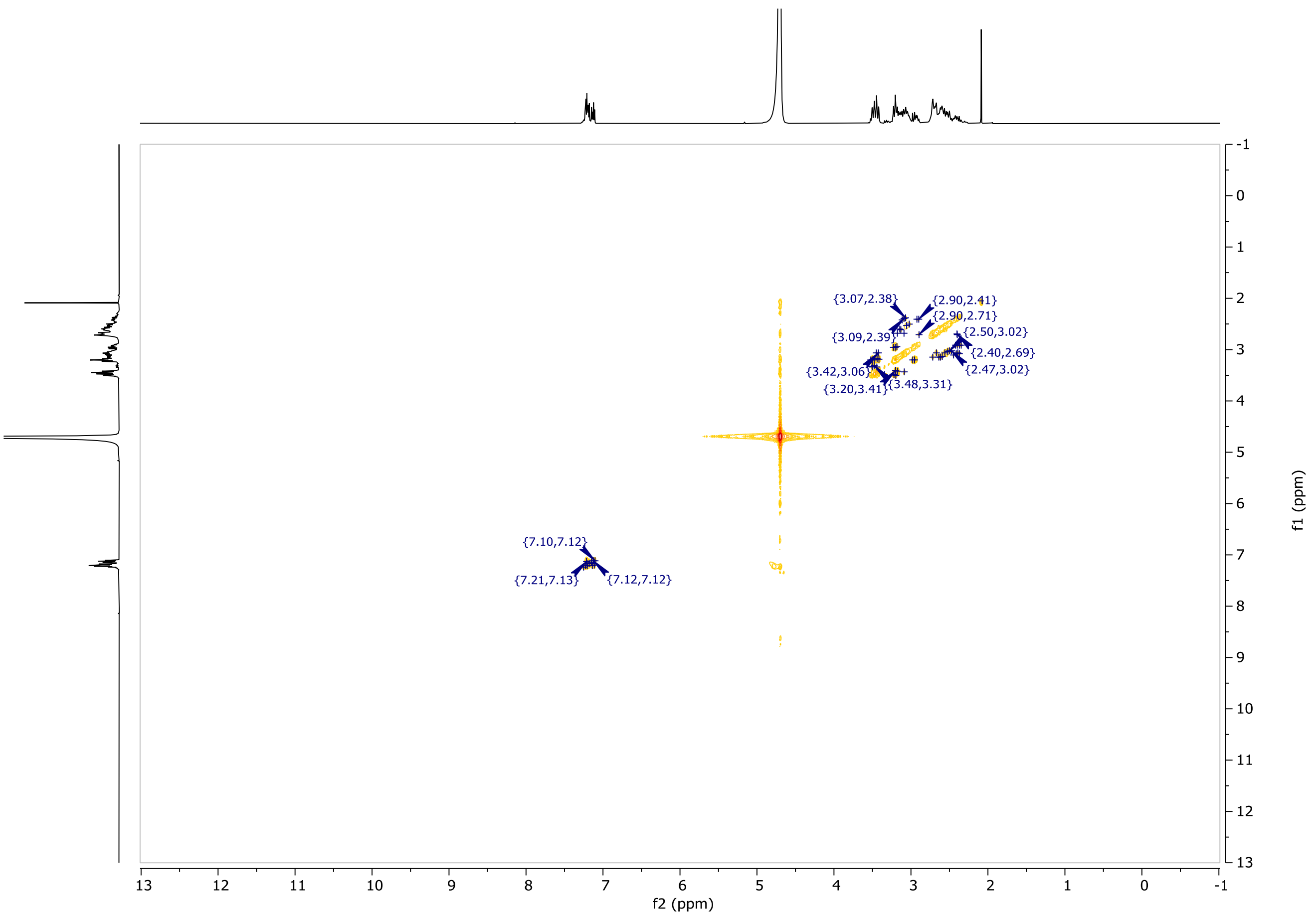

Figure S31. ^{1}H-^{1}H COSY NMR (600 MHz, D_2O, 25 °C): [^{nat}Hg][Hg(*p*-SCN-Bn-TCMC)]$^{2+}$ (pD 7.4).

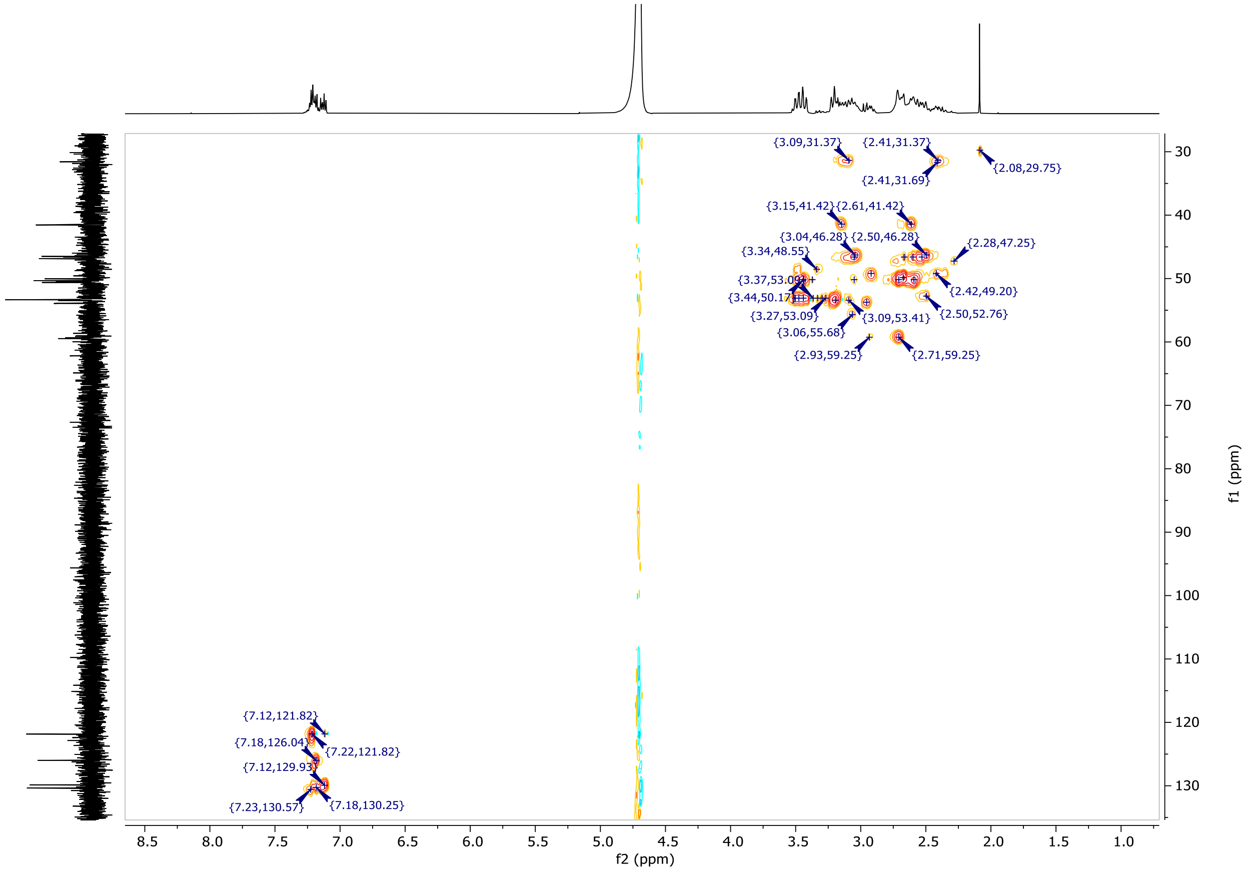

Figure S32. ^{1}H-^{13}C HSQC NMR(600 MHz, D_2O, 25 °C): [^{nat}Hg][Hg(*p*-SCN-Bn-TCMC)]$^{2+}$ (pD 7.4).

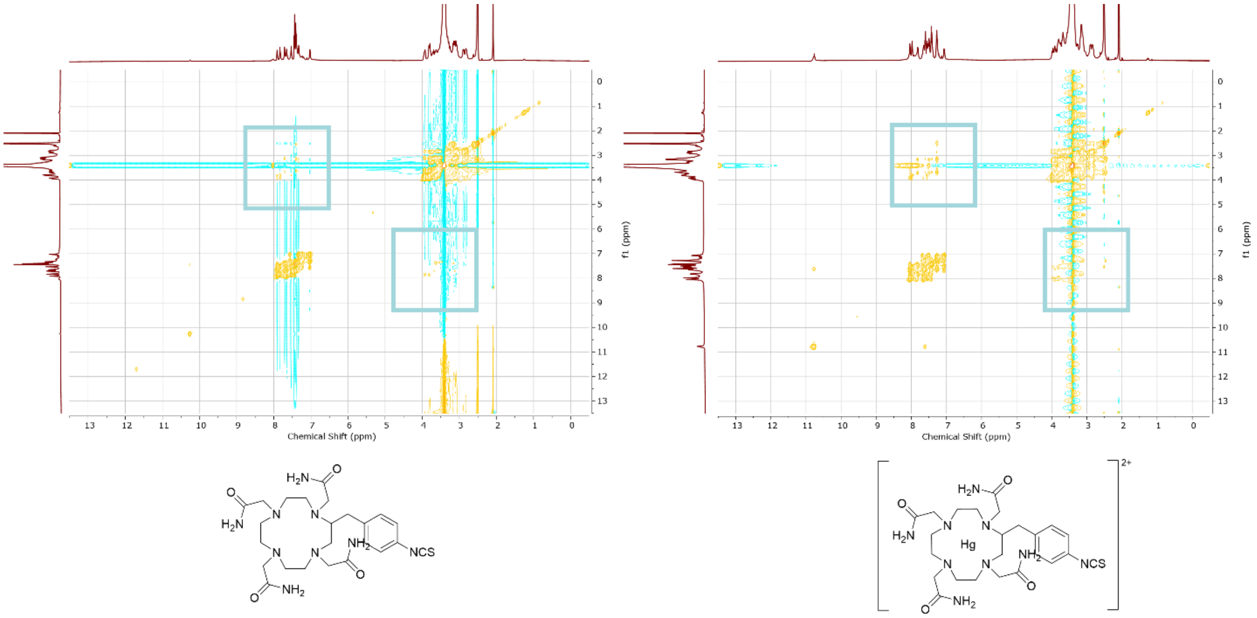

Figure S33. NOESY NMR (600 MHz, DMSO-d^6, 25 °C): *p*-SCN-Bn-TCMC (left) and [Hg(*p*-SCN-Bn-TCMC)]$^{2+}$ (right).

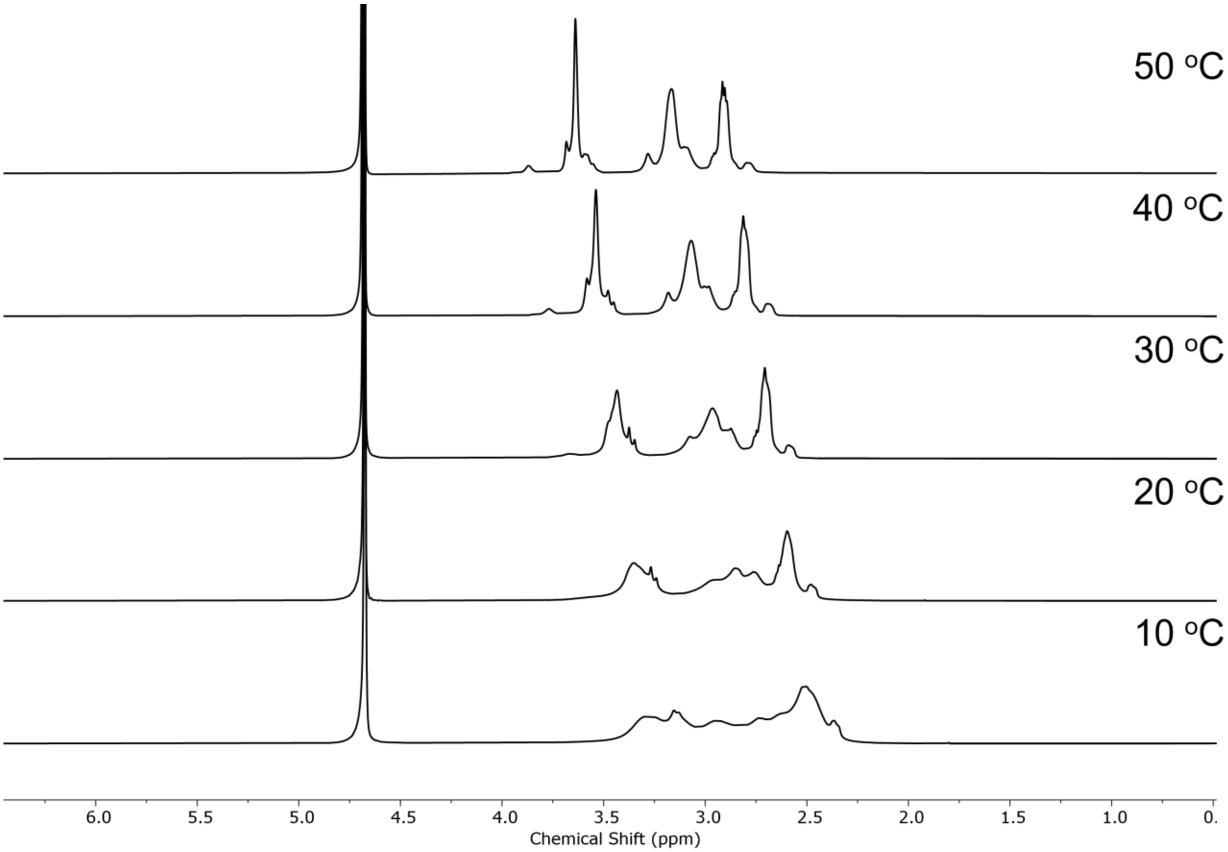

Figure S34. Variable temperature ^{1}H NMR (600 MHz, D_2O,10 – 50 ºC): [^{nat}Hg(TCMC)]$^{2+}$ (pD 7.4).

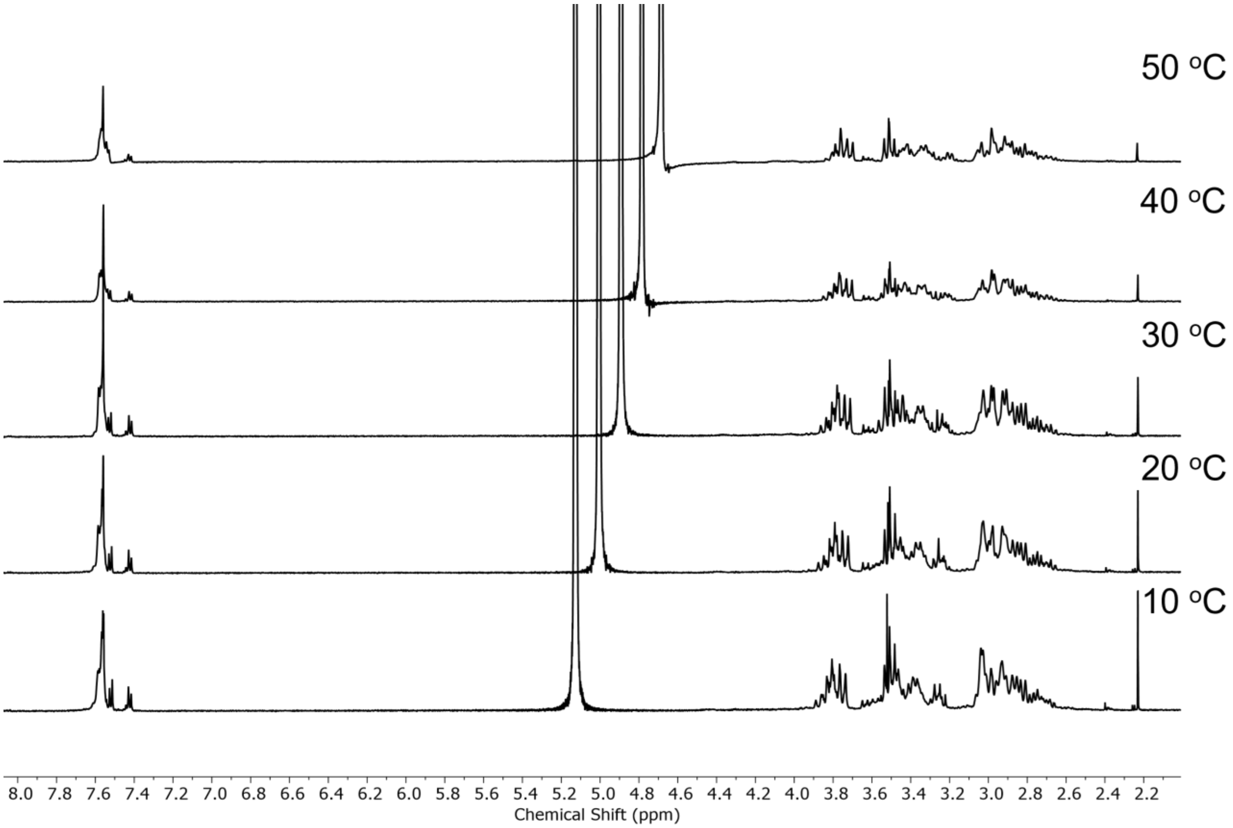

Figure S35. Variable temperature ^{1}H NMR (600 MHz, D_2O,10 – 50 ^oC): [^{nat}Hg][Hg(*p*-SCN-Bn-TCMC)]$^{2+}$ (pD 7.4).

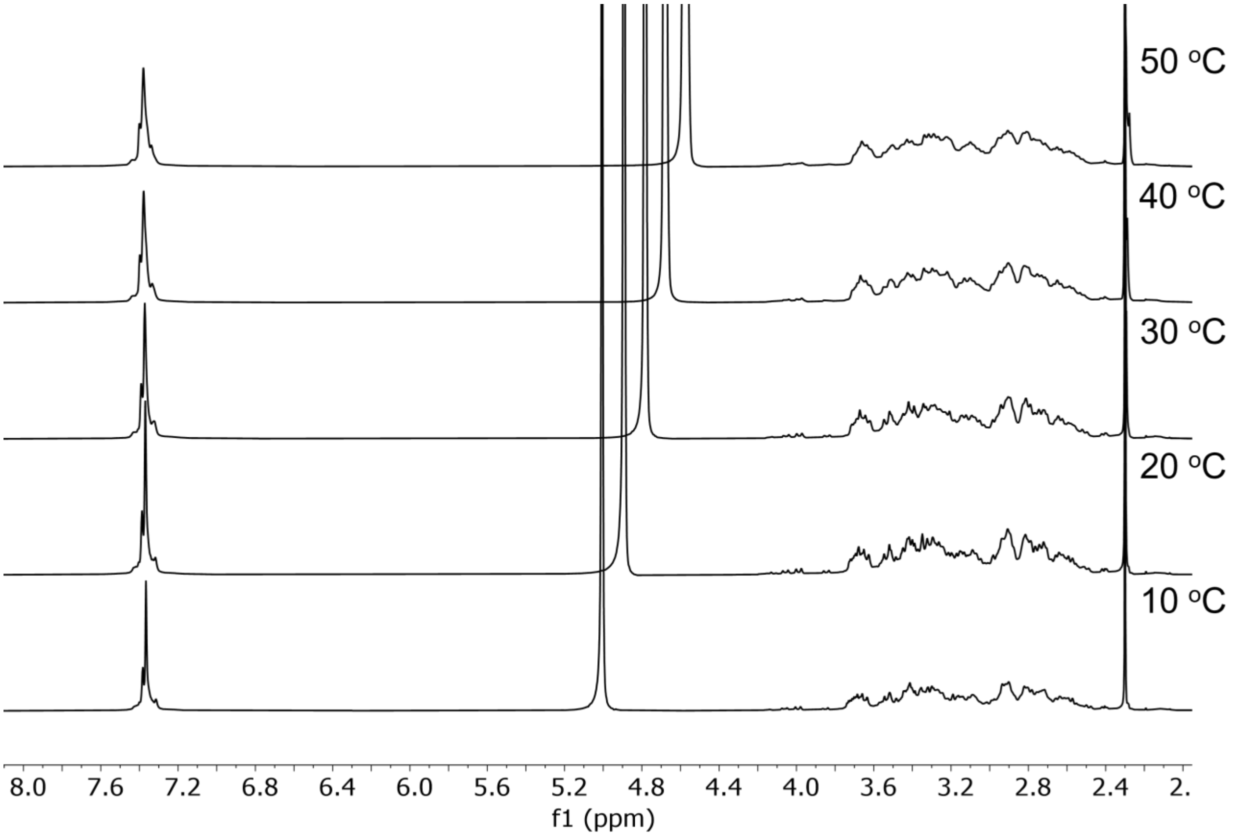

Figure S36. Variable temperature ^{1}H NMR (600 MHz, D_2O,10 – 50 °C): *p*-SCN-Bn-TCMC (pD 7.4)

MS Characterization

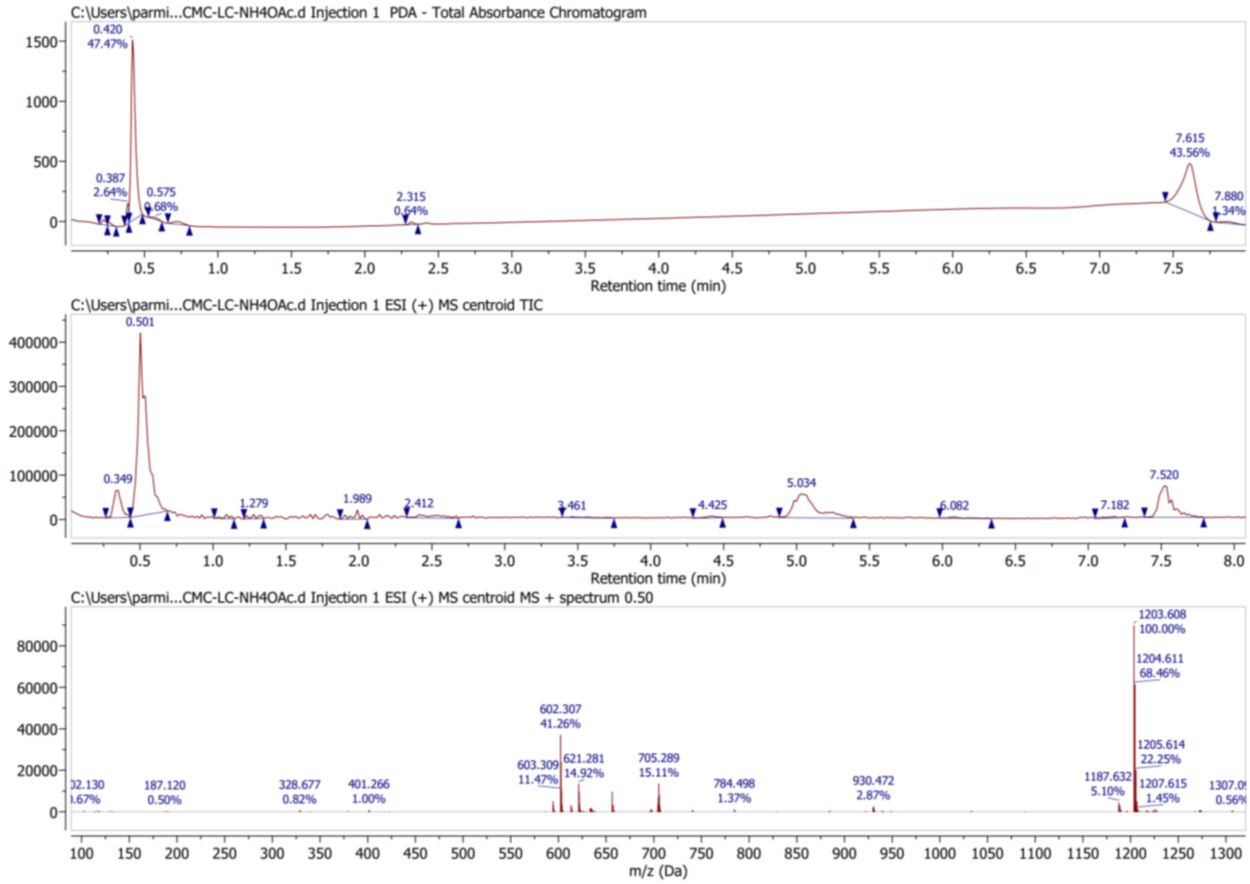

Figure S37. HR-LC-ESI-MS of TCMC-PSMA T_R = 5.034 min, ESI-HRMS *m*/*z* calcd. for $[C_{57}H_{82}N_{14}O_{13}S+H]^+$ 1203.598; found 1203.608 $[M+H]^+$.

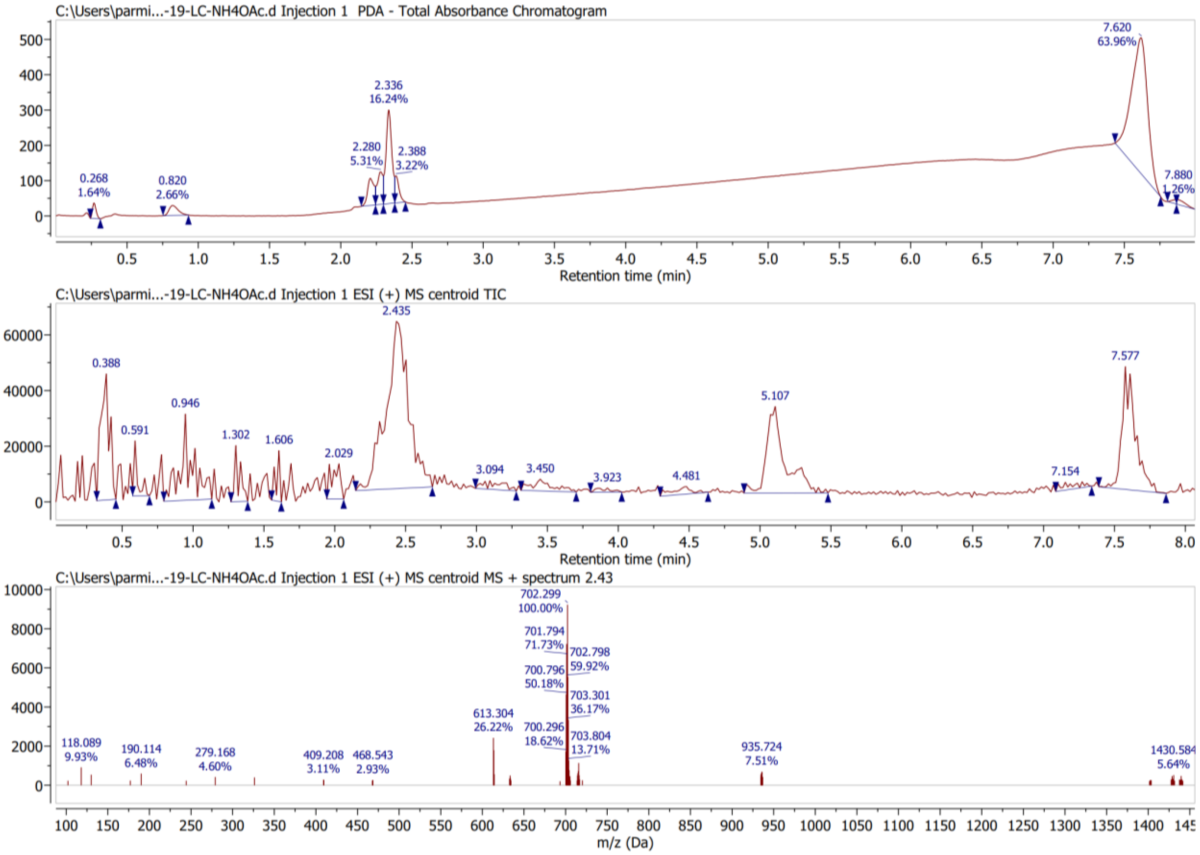

Figure S38. HR-LC-ESI-MS of [^{nat}Hg(TCMC-PSMA)]$^{2+}$ T_R = 2.435 min, ESI-HRMS *m*/*z* calcd. for [$C_{57}H_{82}N_{14}O_{13}SHg$]$^{2+}$ 702.281; found 702.200, [M]$^{2+}$.

Infrared Spectroscopy

Hg(TCMC)$^{2+}$ and [Hg(*p*-SCN-Bn-TCMC)]$^{+}$ Infrared Spectroscopy

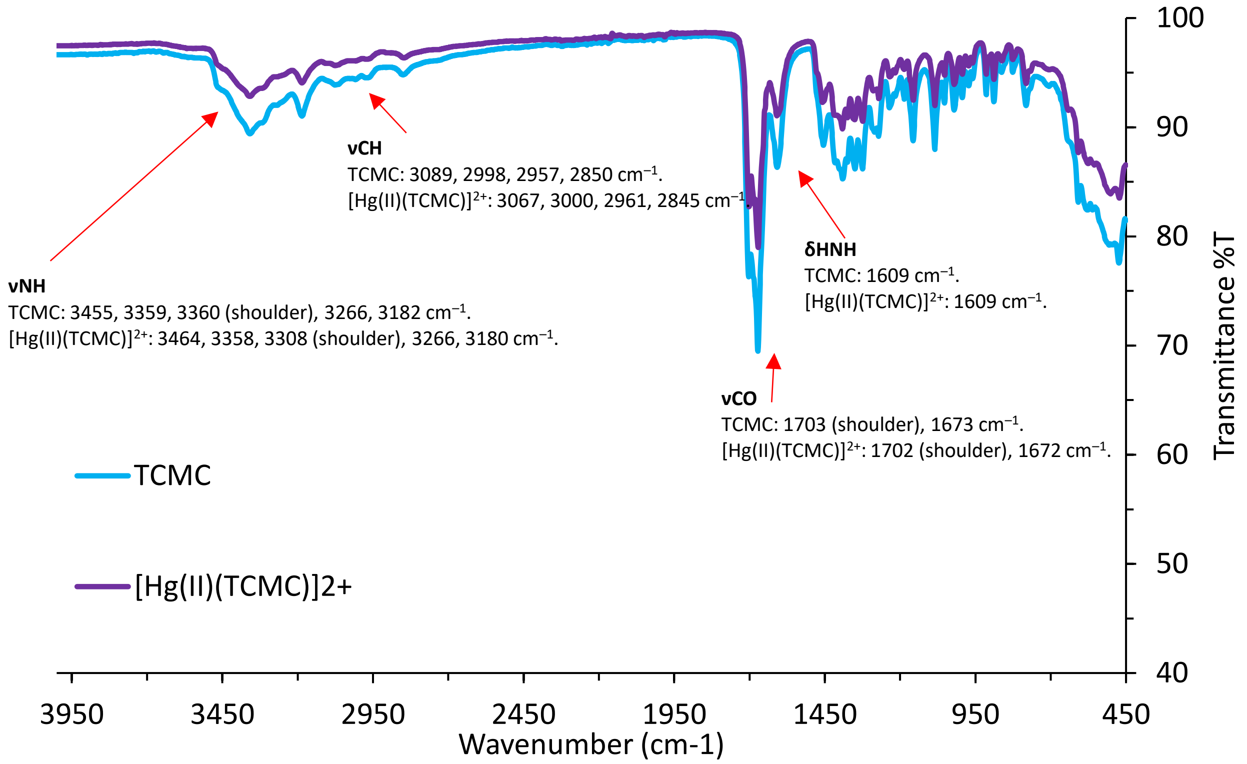

Figure S39. IR spectra of TCMC and [^{nat}Hg][Hg(TCMC)]$^{2+}$ with peak assignment.(Cuenot et al. 2008)

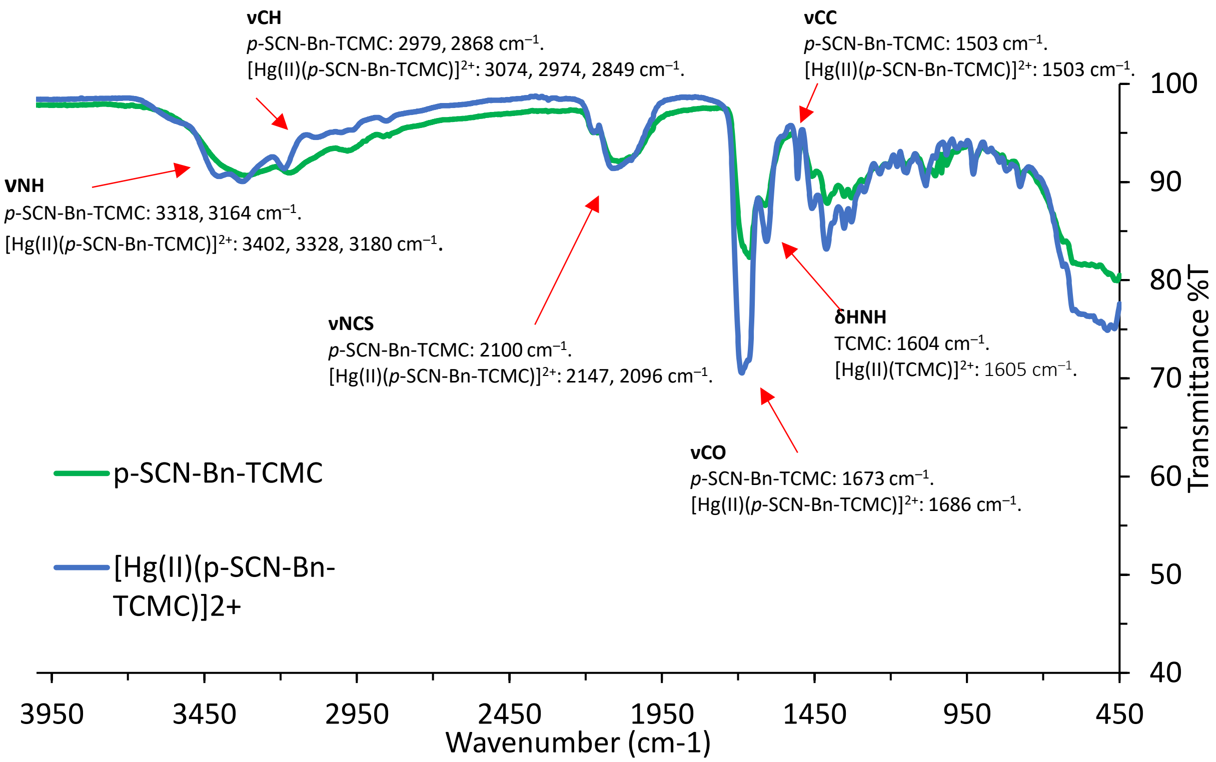

Figure S40. IR spectra of p-SCN-Bn-TCMC and [^{nat}Hg][Hg(*p*-SCN-Bn-TCMC)]$^{2+}$ with peak assignment.

UV-Vis Spectroscopy

[Hg(TCMC)]$^{2+}$ and [Hg(*p*-SCN-Bn-TCMC)]$^{2+}$ UV-Vis spectroscopy.

UV-Vis data was collected for the ligands (TCMC and *p*-SCN-Bn-TCMC) with varying molar ratio of $^{nat}Hg^{2+}$ (0-1 eq.). This data was collected to determine if the metal-ligand compound and free ligand absorbance could provide further information on the coordination complex in the solution. The absorbance of *p*-SCN-Bn-TCMC was found in the UV region, producing two overlapping peaks (λ_{max}= 272 nm, ε= 27,900 Lcm^{-1}mol^{-1} and λ_{max}= 282 nm, ε= 24,600 Lcm^{-1}mol^{-1}) (Gawronski, Kwit, and Skowronek 2009) (**Figure S41**); these bands were attributed to charge

transfer absorptions of the benzyl-isothiocyanate (Atkins et al. 2008). The absorbance of these peaks decreased with increasing metal ratio, which may indicate complex formation and subsequent precipitation.

The TCMC ligand had minimal absorbance above baseline (**Figure S41**). This absorbance decreased as the metal concentration increased, similar to the *p*-SCN-Bn-TCMC spectra. Again, this indicated that the metal-ligand complex is precipitating, but these decreases in absorbance were not as drastic as those observed with the *p*-SCN-Bn-TCMC complexes.

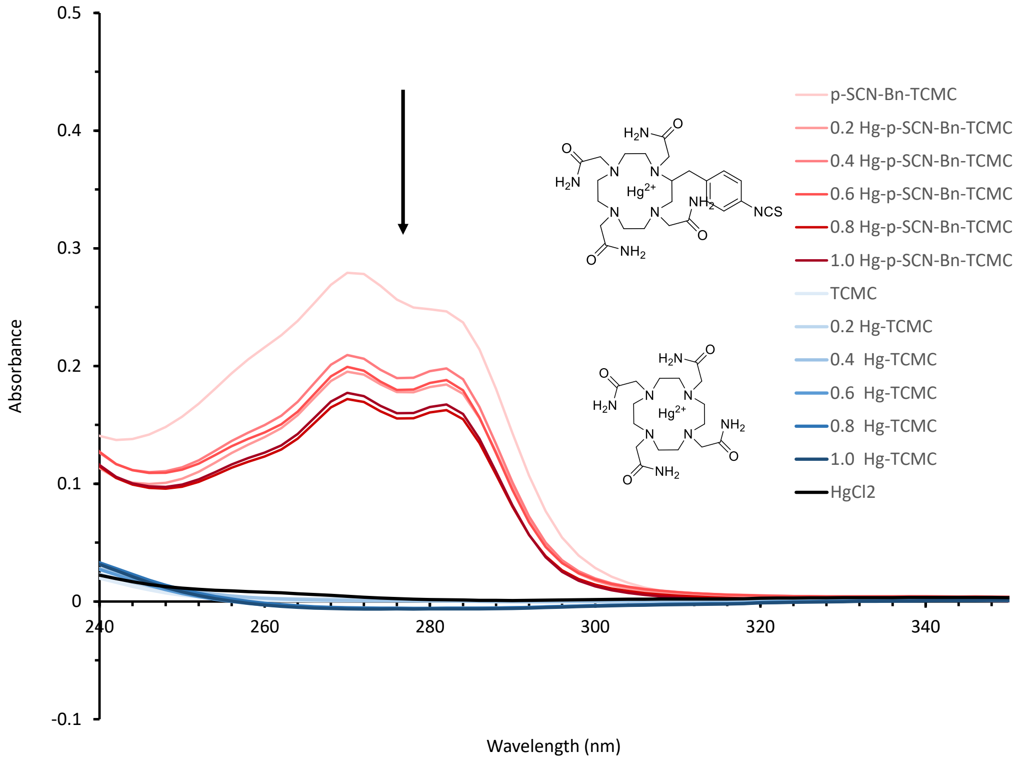

Figure S41. UV-Vis spectra of TCMC and p-SCN-Bn-TCMC with addition of increasing amounts (by 0.2 eq.) of $HgCl_2$.

Radiolabeling and Radiolabeling Method Development

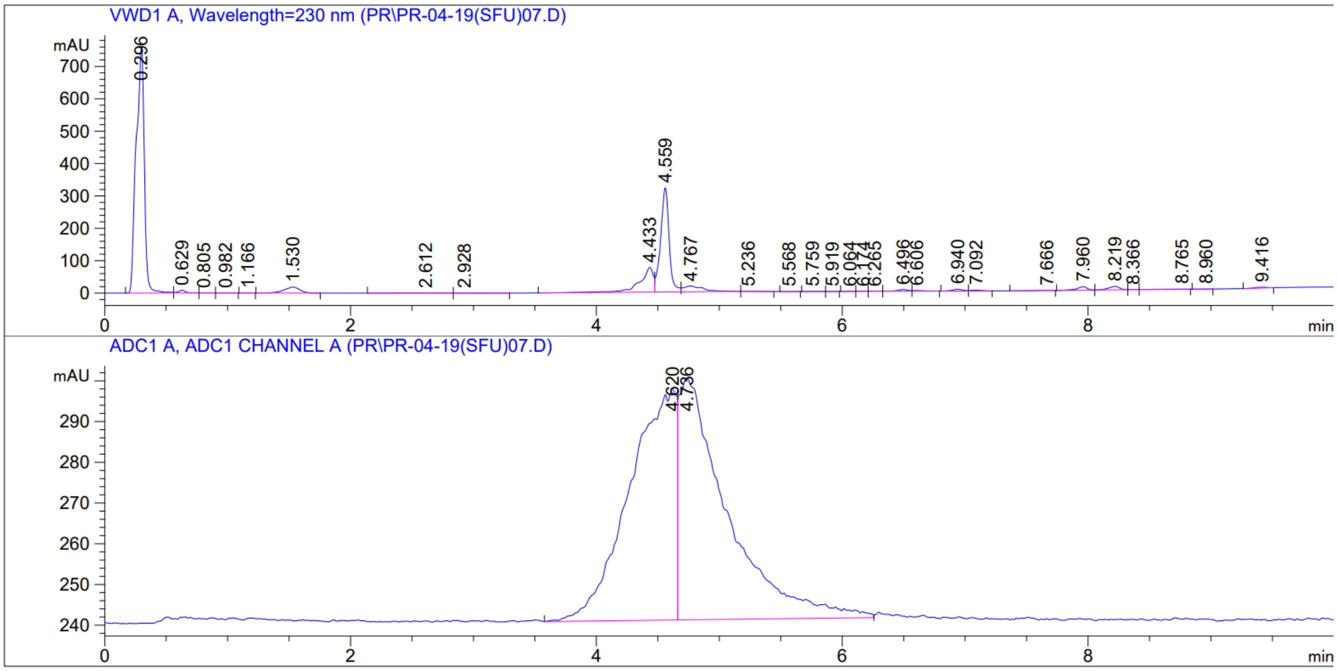

Figure S42. Radio-HPLC of chromatogram of **[$^{197m/g}$Hg][Hg(TCMC-PSMA)]$^{2+}$**, T_R = 4.736 min; Method; A: 90% H_2O 10% MeCN 6 mM NH_4OAc, B: 10% H_2O 90% MeCN 6 mM NH_4OAc. Method: 0% B (0-0.5 min), 0-100% B (0.5-7 min), 100% B (7-8 min), 100-0% B (8-9.5 min), 0% B (9.5-10 min).

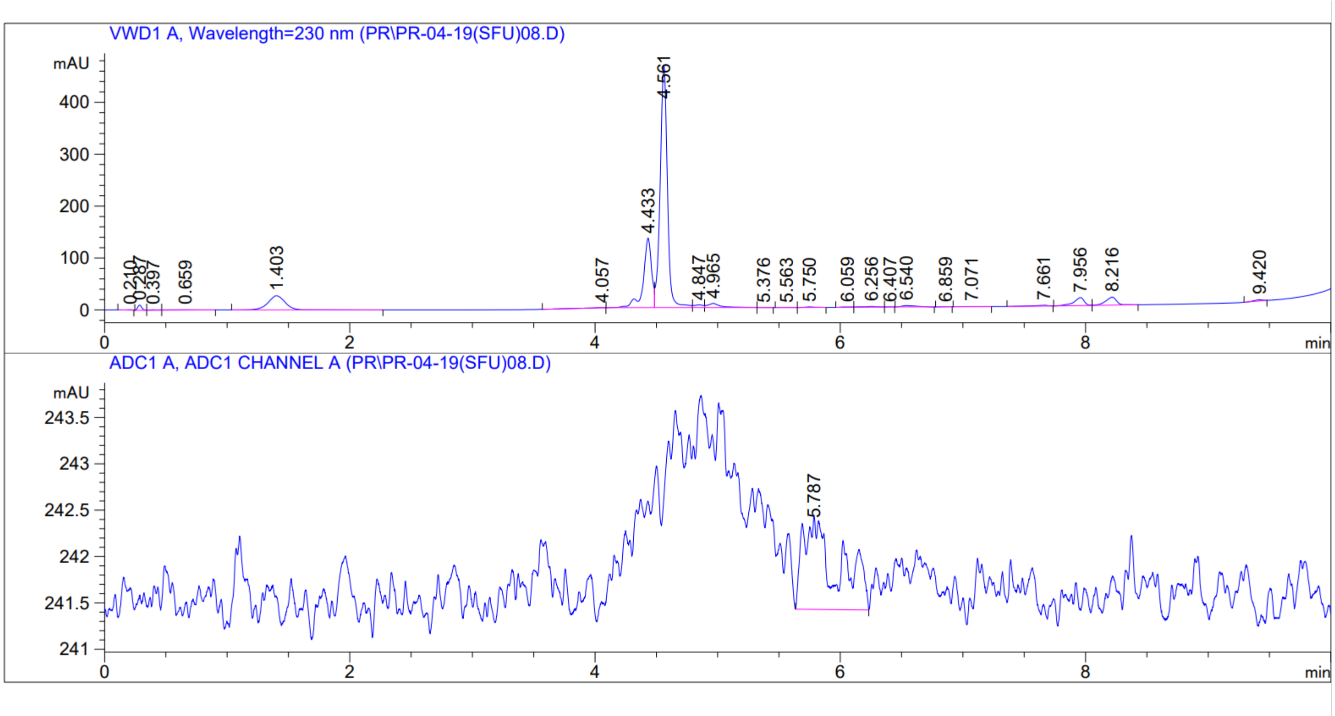

Figure S43. HPLC chromatogram of **[^{nat}Hg(TCMC-PSMA)]$^{2+}$**, T_R = 4.561 min; Method; A: 90% H_2O 10% MeCN 6 mM NH_4OAc, B: 10% H_2O 90% MeCN 6 mM NH_4OAc. Method: 0% B (0-0.5 min), 0-100% B (0.5-7 min), 100% B (7-8 min), 100-0% B (8-9.5 min), 0% B (9.5-10 min).

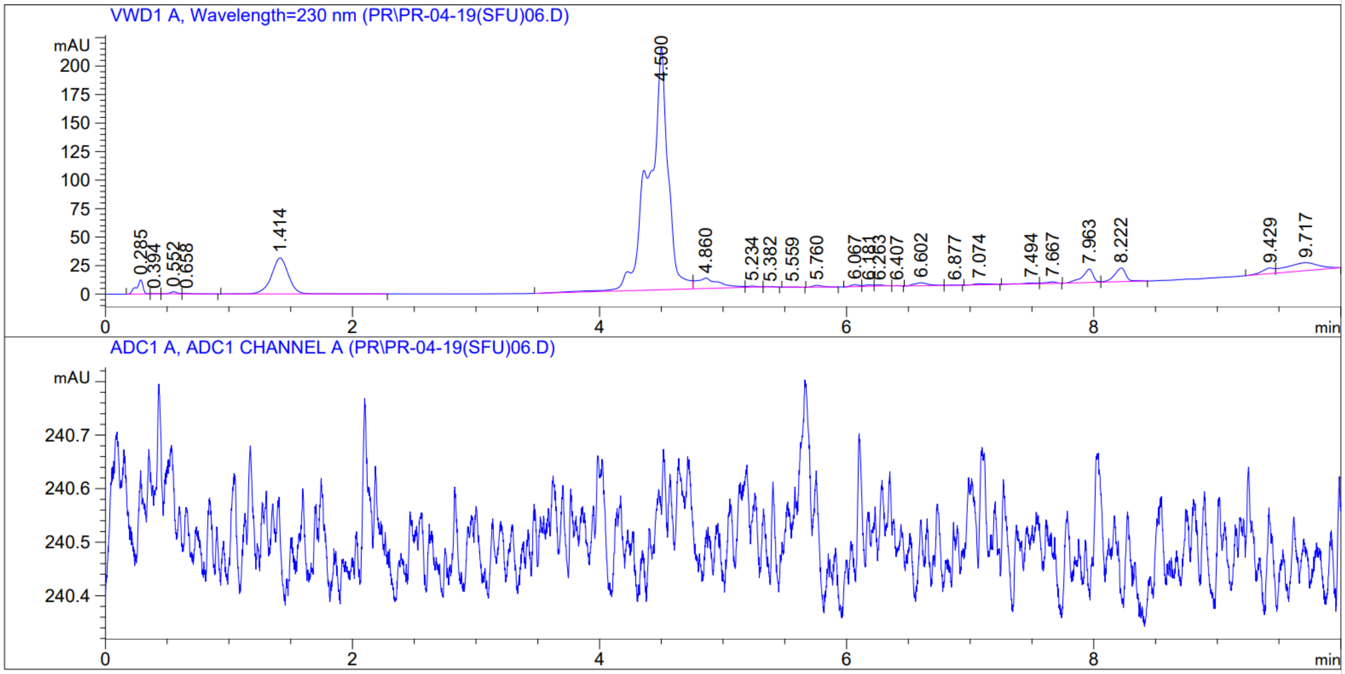

Figure S44. HPLC chromatogram of **TCMC-PSMA**, T_R = 4.500 min; Method; A: 90% H_2O 10% MeCN 6 mM NH_4OAc, B: 10% H_2O 90% MeCN 6 mM NH_4OAc. Method: 0% B (0-0.5 min), 0-100% B (0.5-7 min), 100% B (7-8 min), 100-0% B (8-9.5 min), 0% B (9.5-10 min).

Improvements made to the HPLC method for [$^{197m/g}$Hg][Hg(TCMC-PSMA)]$^{2+}$.

Improvements that led to the successful HPLC conditions could be the exchange of slightly acidic mobile phases (H_2O and MeCN, 0.1% TFA) to neutral mobile phases (H_2O and MeCN, 6 mM NH_4OAc) and the change in column length from 100 mm to 50 mm. Regardless of these achievements the non-isolated radiochemical yield (%RCY) of the reaction can not be confirmed by radio-HPLC at this time, as under all tested conditions thus far the free activity will not elute off the HPLC. Further optimization is still required for radio-HPLC uses as a tool for $^{197m/g}Hg^{2+}$ tracer purification, aiming to achieve the tracer in a small volume with an appropriate amount of activity for animal studies (2-10 MBq in 100 µL).

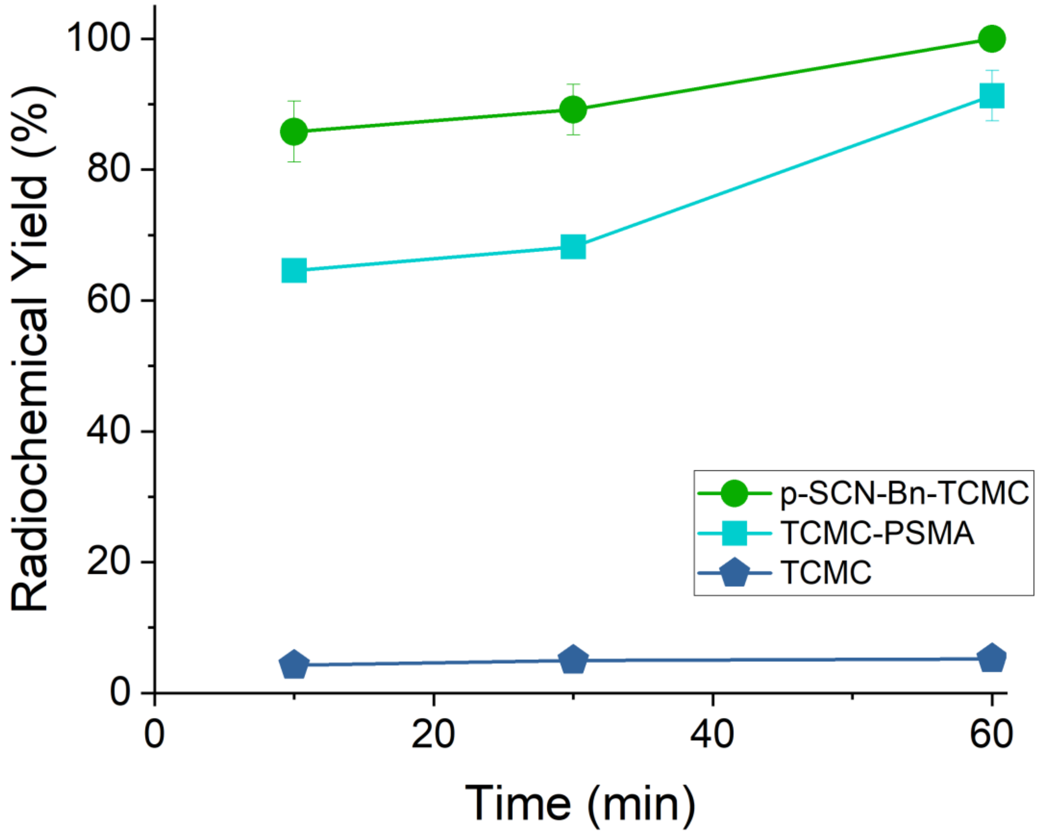

Figure S45. The %RCY of TCMC, *p*-SCN-Bn-TCMC and TCMC-PSMA at [ligand] = 10^{-4} M over 60 min, at 80ºC (n = 3).

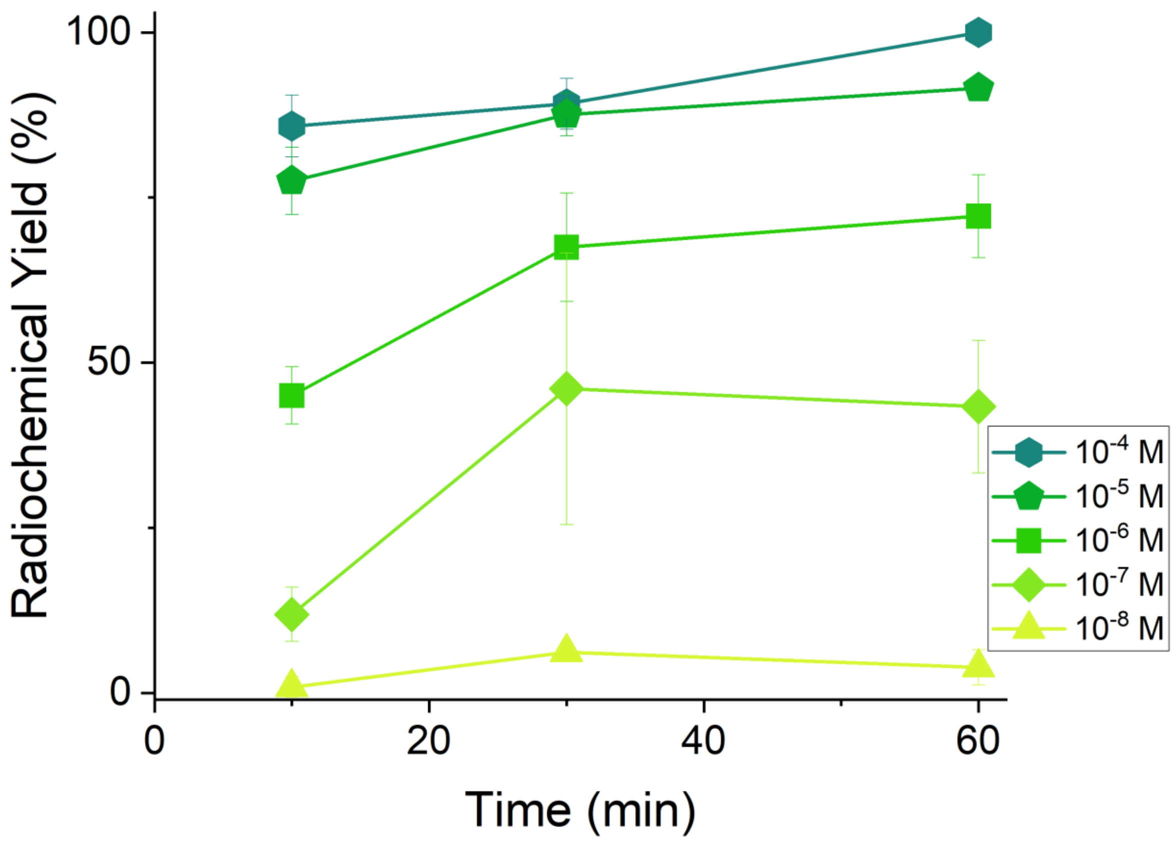

Figure S46. The %RCY of *p*-SCN-Bn-TCMC at varying ligand concentrations 10^{-4} M – 10^{-8} M between 10 – 60 min at 80 ºC (n = 3).

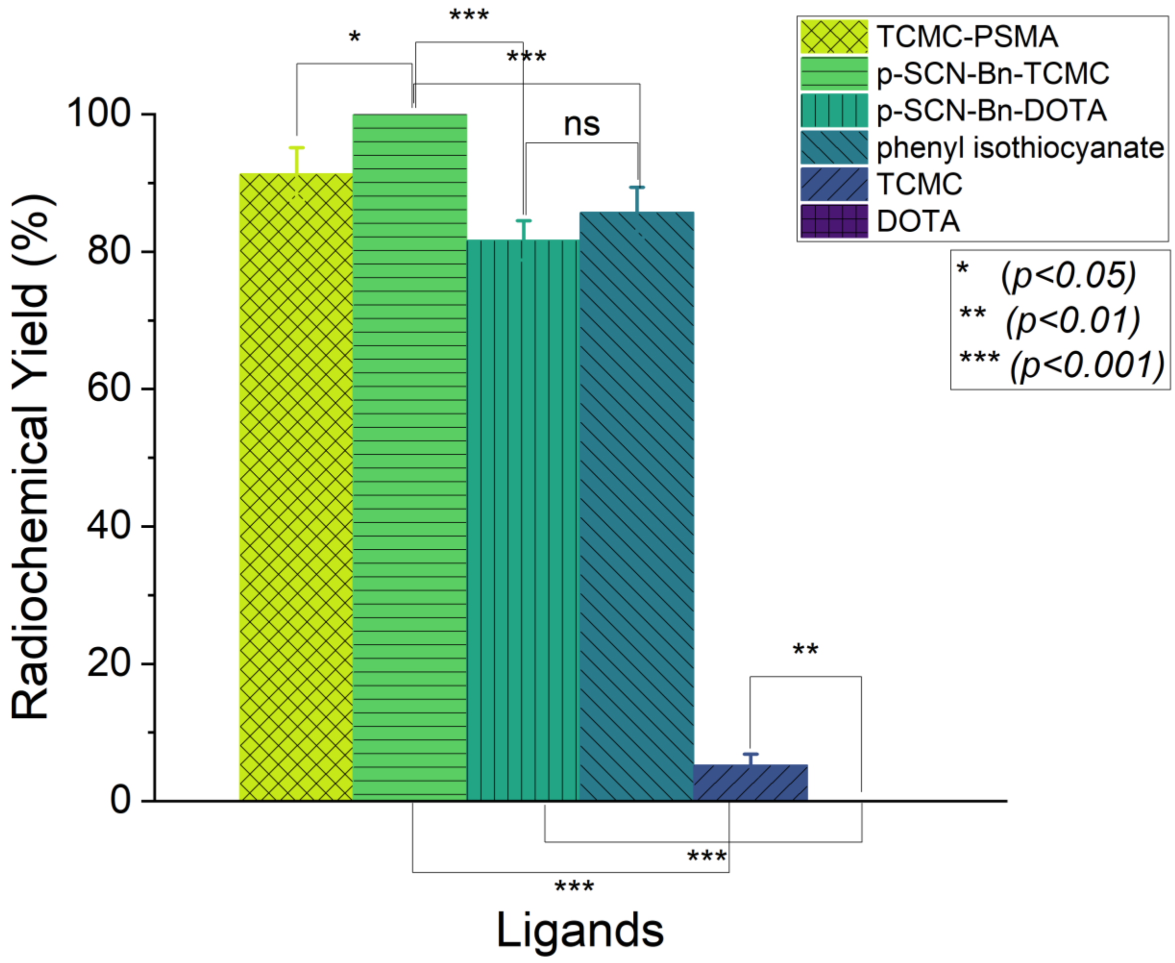

Figure S47. The %RCY of Commercal Cylen ligands (TCMC-PSMA, *p*-SCN-Bn-TCMC, *p*-SCN-Bn-DOTA, phenyl isothiocyanate, TCMC and DOTA) at a ligand concentration of 10^{-4} M after 60 min at 80 ºC (n = 3). Error bars repersent the SD, a two-way ANOVA was used to calculate the significant differences.

DFT Calculations – Cartesian Coordinates & Electrostatic Potential Maps

Table S1. Cartesian coordinates for TCMC at the B3LYP-D3/6-311G**/IEFPCM(H_2O) level of theory.

Atom	X	Y	Z
**O**	1.319389	3.101397	1.376915
**O**	3.101484	-1.31943	1.377109
**O**	-1.31929	-3.10115	1.377182
**O**	-3.10164	1.319385	1.376755
**N**	1.684487	1.357157	-1.06419

**N**	1.357284	-1.68453	-1.06415
**N**	-1.6844	-1.3572	-1.06408
**N**	-1.35724	1.684449	-1.06428
**N**	2.2822	1.247721	2.25286
**H**	2.721463	0.339655	2.091771
**H**	1.830849	1.42467	3.139453
**N**	1.247364	-2.2816	2.252826
**H**	0.339229	-2.72068	2.091647
**H**	1.424187	-1.83007	3.139351
**N**	-2.28222	-1.24747	2.253022
**H**	-2.72137	-0.33937	2.091858
**H**	-1.83086	-1.42435	3.139621
**N**	-1.24754	2.281563	2.252572
**H**	-0.33948	2.720815	2.091474
**H**	-1.42439	1.830031	3.13909
**C**	2.185814	0.454175	-2.09908
**H**	3.093062	0.862881	-2.58345
**H**	1.426891	0.403232	-2.88077
**C**	2.50722	-0.9676	-1.62429
**H**	2.94759	-1.51753	-2.47703
**H**	3.275596	-0.92846	-0.85549
**C**	0.454277	-2.18593	-2.09897
**H**	0.862915	-3.09328	-2.58319
**H**	0.403395	-1.42712	-2.88077
**C**	-0.96749	-2.50721	-1.62408
**H**	-1.51746	-2.9477	-2.47674
**H**	-0.92834	-3.27547	-0.85517
**C**	-2.18576	-0.45433	-2.09905
**H**	-3.09303	-0.86307	-2.58333
**H**	-1.42687	-0.40349	-2.88078
**C**	-2.50715	0.967472	-1.62438
**H**	-2.9475	1.517327	-2.47718
**H**	-3.27555	0.928422	-0.85559
**C**	-0.4542	2.185749	-2.09913
**H**	-0.86285	3.093032	-2.58347
**H**	-0.40328	1.426842	-2.88084
**C**	0.967559	2.507111	-1.62429
**H**	1.517507	2.947515	-2.47701
**H**	0.928419	3.275457	-0.85546
**C**	2.698283	1.755998	-0.09056
**H**	3.279814	2.637049	-0.41356

**H**	3.394569	0.934538	0.053118
**C**	2.038927	2.096198	1.245363
**C**	1.756066	-2.69825	-0.09042
**H**	2.637159	-3.27979	-0.41327
**H**	0.934594	-3.39453	0.053217
**C**	2.096125	-2.03875	1.245477
**C**	-2.6982	-1.7559	-0.09041
**H**	-3.27977	-2.63696	-0.41329
**H**	-3.39446	-0.93439	0.05317
**C**	-2.03885	-2.09598	1.24556
**C**	-1.75618	2.698225	-0.09068
**H**	-2.6373	3.279659	-0.41367
**H**	-0.9348	3.39461	0.052988
**C**	-2.09629	2.038724	1.245195

Table S2. Cartesian coordinates for *p*-SCN-Bn-TCMC at the B3LYP-D3/6-311G**/IEFPCM(H_2O) level of theory.

Atom	X	Y	Z
**O**	-4.09548	-2.87757	1.254473
**O**	-5.55446	1.662005	0.086678
**O**	-1.23549	3.425073	1.629043
**O**	0.235331	-1.10726	2.750243
**N**	-3.31337	-1.3948	-1.31304
**N**	-2.98492	1.644027	-1.57253
**N**	-0.15972	1.370458	-0.37494
**N**	-0.43041	-1.68616	-0.1864
**N**	-5.20294	-0.90767	1.418156
**H**	-5.50098	-0.03467	0.983985
**H**	-5.15954	-0.95015	2.42656
**N**	-4.05578	2.72982	1.408804
**H**	-3.13141	3.145032	1.521446
**H**	-4.53459	2.404674	2.236805
**N**	-0.7079	1.642393	2.9239
**H**	-0.27066	0.725142	3.007274
**H**	-1.43072	1.890302	3.584704
**N**	-1.84934	-1.98538	2.857489
**H**	-2.62906	-2.45055	2.393232
**H**	-2.04174	-1.45801	3.697405
**C**	-3.38264	-0.64124	-2.56975
**H**	-4.05386	-1.13365	-3.29778

**H**	-2.39039	-0.66169	-3.0222
**C**	-3.84398	0.813782	-2.42467
**H**	-3.9297	1.239316	-3.44201
**H**	-4.84546	0.838699	-1.99774
**C**	-1.7404	2.041385	-2.23681
**H**	-1.90393	2.887845	-2.92938
**H**	-1.4178	1.20748	-2.86149
**C**	-0.60568	2.436043	-1.28569
**H**	0.235012	2.805629	-1.9022
**H**	-0.92491	3.275211	-0.66981
**C**	0.689966	0.387785	-1.05771
**H**	1.69937	0.798425	-1.23395
**H**	0.265462	0.210485	-2.04747
**C**	0.8471	-0.96075	-0.33327
**H**	1.174503	-0.7601	0.68593
**C**	-0.94373	-2.30829	-1.41274
**H**	-0.45123	-3.27342	-1.62401
**H**	-0.68994	-1.65577	-2.24918
**C**	-2.45499	-2.58147	-1.42028
**H**	-2.67822	-3.14864	-2.34359
**H**	-2.70862	-3.23959	-0.59078
**C**	-4.64812	-1.73701	-0.80432
**H**	-5.03394	-2.67532	-1.23634
**H**	-5.34377	-0.94397	-1.0716
**C**	-4.62354	-1.89403	0.715651
**C**	-3.71111	2.784621	-1.00371
**H**	-4.4019	3.248234	-1.72718
**H**	-2.99629	3.547395	-0.69984
**C**	-4.52609	2.34244	0.212142
**C**	0.486644	1.930894	0.819923
**H**	1.137741	2.788219	0.581736
**H**	1.109253	1.167654	1.28224
**C**	-0.56424	2.402098	1.826237
**C**	-0.43387	-2.5952	0.965708
**H**	0.507328	-3.1586	1.07086
**H**	-1.23058	-3.32566	0.839605
**C**	-0.65343	-1.82724	2.270347
**C**	1.971097	-1.8074	-1.01068
**H**	1.789045	-1.87774	-2.08632
**H**	1.910268	-2.82671	-0.6156
**C**	3.368956	-1.28453	-0.77272

**C**	4.146251	-0.77975	-1.82259
**C**	3.928186	-1.29805	0.513819
**C**	5.433827	-0.30164	-1.60913
**H**	3.739436	-0.76198	-2.82753
**C**	5.211481	-0.82488	0.750975
**H**	3.351949	-1.68712	1.346172
**C**	5.968586	-0.32288	-0.31599
**H**	6.027127	0.084635	-2.42826
**H**	5.635831	-0.84143	1.746899
**N**	7.243474	0.148712	-0.09131
**C**	8.331413	0.55383	0.097937
**S**	9.814626	1.105088	0.355556

Table S3. Cartesian coordinates for $[Hg(TCMC)]^{2+}$ at the B3LYP-D3/6-311G**/SDD/IEFPCM(H_2O) level of theory.

Atom	X	Y	Z
**O**	-0.71193	3.324916	-1.34294
**O**	-3.32829	-0.70213	-1.39172
**O**	0.688674	-3.33061	-1.42678
**O**	3.313968	0.695975	-1.38434
**N**	-1.34801	1.584884	1.046317
**N**	-1.62151	-1.37496	1.009664
**N**	1.338512	-1.64703	1.001424
**N**	1.610252	1.311478	1.03604
**N**	-2.02764	1.727851	-2.27222
**H**	-2.67158	0.949555	-2.13893
**H**	-1.59331	1.852397	-3.17549
**N**	-1.73212	-2.01906	-2.32204
**H**	-0.95748	-2.66717	-2.18787
**H**	-1.84568	-1.57444	-3.22179
**N**	1.996668	-1.70643	-2.32031
**H**	2.645457	-0.93519	-2.17073
**H**	1.557475	-1.80628	-3.22433
**N**	1.696533	2.013273	-2.27532
**H**	0.924815	2.661331	-2.12506
**H**	1.805696	1.58612	-3.18399
**C**	-2.09901	0.846397	2.099023
**H**	-2.92522	1.459858	2.47258
**H**	-1.42121	0.688114	2.93781
**C**	-2.65916	-0.48336	1.599658

**H**	-3.15997	-0.99003	2.43056
**H**	-3.4021	-0.30983	0.826516
**C**	-0.89418	-2.13865	2.058418
**H**	-1.51107	-2.96965	2.415438
**H**	-0.74534	-1.4715	2.907341
**C**	0.441414	-2.6921	1.568883
**H**	0.93851	-3.2014	2.400387
**H**	0.277712	-3.42684	0.785806
**C**	2.093575	-0.93427	2.066651
**H**	2.921102	-1.55647	2.422465
**H**	1.418884	-0.79659	2.911456
**C**	2.651411	0.407122	1.599035
**H**	3.154632	0.893048	2.440817
**H**	3.391819	0.253341	0.819283
**C**	0.888645	2.050096	2.108795
**H**	1.508023	2.871893	2.482332
**H**	0.743168	1.362319	2.941657
**C**	-0.44843	2.615894	1.635873
**H**	-0.94298	3.106362	2.480153
**H**	-0.28609	3.368421	0.869545
**C**	-2.29962	2.206674	0.084451
**H**	-2.68564	3.147416	0.488873
**H**	-3.1344	1.528888	-0.06486
**C**	-1.6134	2.482464	-1.24658
**C**	-2.23229	-2.31264	0.027407
**H**	-3.17813	-2.70303	0.415497
**H**	-1.55367	-3.14635	-0.12409
**C**	-2.49183	-1.60934	-1.2984
**C**	2.283921	-2.24707	0.019869
**H**	2.670465	-3.19759	0.400083
**H**	3.119081	-1.5672	-0.11762
**C**	1.589015	-2.48928	-1.31355
**C**	2.217505	2.271945	0.073411
**H**	3.164522	2.654038	0.466556
**H**	1.537717	3.108026	-0.05871
**C**	2.471361	1.59466	-1.26669
**Hg**	-0.00777	-0.0229	0.146558

Table S4. Calculated Energies for all Investigated Conformers of the for $[Hg(TCMC)]^{2+}$ at the B3LYP-D3/6-311G**/SDD/IEFPCM(H_2O) level of theory.

Coordination Number	E_{ZP}, unscaled (Hartree)	G_{corr} (Hartree)	ΔG_{solv} (Hartree)	ΔG (kcal/mol)
4	0.144099	0.101266	-0.164891	0.0
6	0.144099	0.101266	-0.199786	286.8
8	0.144099	0.101266	0.213672	324.2

Table S5. Cartesian coordinates for $[Hg(p\text{-SCN-Bn-TCMC})]^{2+}$ at the B3LYP-D3/6-311G**/SDD/IEFPCM(H_2O) level of theory.

Atom	X	Y	Z
**O**	-3.82543	-2.82142	1.644741
**O**	-5.48473	1.516032	0.205798
**O**	-1.17539	3.505153	1.18248
**O**	0.544657	-0.83129	2.638088
**N**	-3.31478	-1.48103	-1.0455
**N**	-3.04028	1.422167	-1.58389
**N**	-0.20719	1.301731	-0.66259
**N**	-0.46058	-1.62341	-0.14481
**N**	-4.99072	-0.88052	1.821271
**H**	-5.39307	-0.06477	1.362816
**H**	-4.90233	-0.8774	2.827411
**N**	-3.9924	2.767279	1.372271
**H**	-3.09389	3.24647	1.395641
**H**	-4.43927	2.535684	2.247977
**N**	-0.49795	1.885728	2.622566
**H**	0.028207	1.029724	2.790278
**H**	-1.1583	2.19031	3.323571
**N**	-1.56181	-1.59018	3.01669
**H**	-2.35434	-2.15601	2.716195
**H**	-1.6583	-1.0189	3.843914
**C**	-3.56306	-0.89668	-2.39387
**H**	-4.32984	-1.475	-2.91856
**H**	-2.64352	-0.99456	-2.97021
**C**	-4.00072	0.563645	-2.32785
**H**	-4.12962	0.941812	-3.34739
**H**	-4.96006	0.648243	-1.82496
**C**	-1.90101	1.828962	-2.44744
**H**	-2.20804	2.626306	-3.13215

**H**	-1.62992	0.972463	-3.06436
**C**	-0.69739	2.309163	-1.64491
**H**	0.108719	2.574326	-2.3364
**H**	-0.95177	3.202453	-1.08104
**C**	0.656302	0.288914	-1.31731
**H**	1.638078	0.720137	-1.53392
**H**	0.208793	0.025914	-2.27528
**C**	0.846029	-0.96224	-0.45409
**H**	1.224413	-0.65452	0.517594
**C**	-0.99072	-2.4375	-1.27373
**H**	-0.47187	-3.39786	-1.34032
**H**	-0.78377	-1.9004	-2.19842
**C**	-2.48736	-2.71598	-1.14707
**H**	-2.80579	-3.30627	-2.01228
**H**	-2.68916	-3.30791	-0.25859
**C**	-4.6182	-1.78375	-0.38598
**H**	-5.02443	-2.72276	-0.774
**H**	-5.31454	-0.98471	-0.62201
**C**	-4.45679	-1.89345	1.125313
**C**	-3.72769	2.62001	-1.02826
**H**	-4.42266	3.035013	-1.76479
**H**	-2.98005	3.374313	-0.80128
**C**	-4.49693	2.259286	0.23903
**C**	0.535617	1.984398	0.435689
**H**	1.14283	2.79903	0.031923
**H**	1.200216	1.269019	0.910598
**C**	-0.44795	2.543599	1.456307
**C**	-0.3637	-2.44708	1.096346
**H**	0.563305	-3.02529	1.108234
**H**	-1.19581	-3.1444	1.110363
**C**	-0.40783	-1.56196	2.338695
**C**	1.900032	-1.89871	-1.08014
**H**	1.684757	-2.06973	-2.13661
**H**	1.854819	-2.87136	-0.5842
**C**	3.289731	-1.3299	-0.921
**C**	4.024541	-0.88865	-2.02361
**C**	3.856932	-1.21118	0.354702
**C**	5.293324	-0.34183	-1.86885
**H**	3.600816	-0.9709	-3.01761
**C**	5.12052	-0.66866	0.530331
**H**	3.302945	-1.54413	1.2253

**C**	5.841422	-0.23141	-0.58906
**H**	5.861501	0.000043	-2.72393
**H**	5.556623	-0.58051	1.516866
**N**	7.100011	0.311795	-0.43031
**C**	8.085457	0.6963	0.096277
**S**	9.444451	1.235835	0.735678
**Hg**	-2.01737	0.076098	-0.00848

Table S6. Cartesian coordinates for [*p*-SCN-Bn-TCMC···Hg]$^{2+}$ at the B3LYP-D3/6-311G**/SDD/IEFPCM(H_2O) level of theory.

Atom	X	Y	Z
**O**	-3.88991	-2.82212	1.379198
**O**	-5.32603	1.691659	0.356327
**O**	-0.89581	3.375358	1.486612
**O**	0.515699	-1.17637	2.51622
**N**	-3.33986	-1.36385	-1.2355
**N**	-2.92125	1.635869	-1.51025
**N**	-0.05221	1.300789	-0.57358
**N**	-0.45837	-1.72941	-0.32657
**N**	-4.86604	-0.79953	1.671488
**H**	-5.19954	0.077194	1.268154
**H**	-4.71888	-0.84968	2.669146
**N**	-3.65546	2.61693	1.57449
**H**	-2.72598	3.036786	1.610277
**H**	-4.07138	2.287668	2.433576
**N**	-0.36297	1.546973	2.713173
**H**	0.069976	0.624472	2.76566
**H**	-1.0361	1.803211	3.420908
**N**	-1.62207	-1.86506	2.804775
**H**	-2.45265	-2.32694	2.430755
**H**	-1.70219	-1.32779	3.655881
**C**	-3.48943	-0.60731	-2.47884
**H**	-4.23308	-1.07668	-3.14881
**H**	-2.53753	-0.65576	-3.00895
**C**	-3.88811	0.856069	-2.28492
**H**	-4.04958	1.299279	-3.28456
**H**	-4.84298	0.910419	-1.76634
**C**	-1.74467	2.002951	-2.29473
**H**	-1.9576	2.848277	-2.97466
**H**	-1.50018	1.157212	-2.93817

**C**	-0.52324	2.373105	-1.45506
**H**	0.277571	2.699976	-2.14346
**H**	-0.75797	3.230145	-0.82709
**C**	0.680486	0.269375	-1.30499
**H**	1.688264	0.624121	-1.57999
**H**	0.159657	0.088849	-2.24581
**C**	0.825	-1.05242	-0.5443
**H**	1.192401	-0.82392	0.452678
**C**	-1.04055	-2.35168	-1.51588
**H**	-0.57523	-3.32602	-1.74362
**H**	-0.8231	-1.70771	-2.36828
**C**	-2.55111	-2.58309	-1.42525
**H**	-2.86294	-3.12022	-2.33958
**H**	-2.76879	-3.24659	-0.59112
**C**	-4.62706	-1.6407	-0.59497
**H**	-5.10659	-2.55227	-0.98756
**H**	-5.30161	-0.80717	-0.77651
**C**	-4.4329	-1.81108	0.907576
**C**	-3.53994	2.784149	-0.8476
**H**	-4.26863	3.303004	-1.49148
**H**	-2.76621	3.499947	-0.57874
**C**	-4.25884	2.321129	0.41524
**C**	0.714732	1.838339	0.553835
**H**	1.365267	2.676897	0.258809
**H**	1.351891	1.05823	0.964297
**C**	-0.24804	2.32774	1.629794
**C**	-0.42821	-2.62653	0.829636
**H**	0.470357	-3.26381	0.856586
**H**	-1.29258	-3.28425	0.786993
**C**	-0.46843	-1.8268	2.127984
**C**	1.91358	-1.94195	-1.21622
**H**	1.74086	-2.01449	-2.2923
**H**	1.835562	-2.9537	-0.80819
**C**	3.292667	-1.40595	-0.94471
**C**	4.08101	-0.85665	-1.96194
**C**	3.787518	-1.39491	0.367994
**C**	5.31721	-0.28676	-1.68824
**H**	3.714996	-0.86004	-2.98152
**C**	5.021694	-0.84101	0.665824
**H**	3.187769	-1.80919	1.169994
**C**	5.775072	-0.27576	-0.36898

**H**	5.919695	0.152268	-2.47223
**H**	5.402031	-0.82671	1.678594
**N**	6.978748	0.329541	-0.06697
**C**	7.945133	0.884158	0.263669
**S**	9.328485	1.655397	0.6808
**Hg**	8.431603	2.895867	2.648972

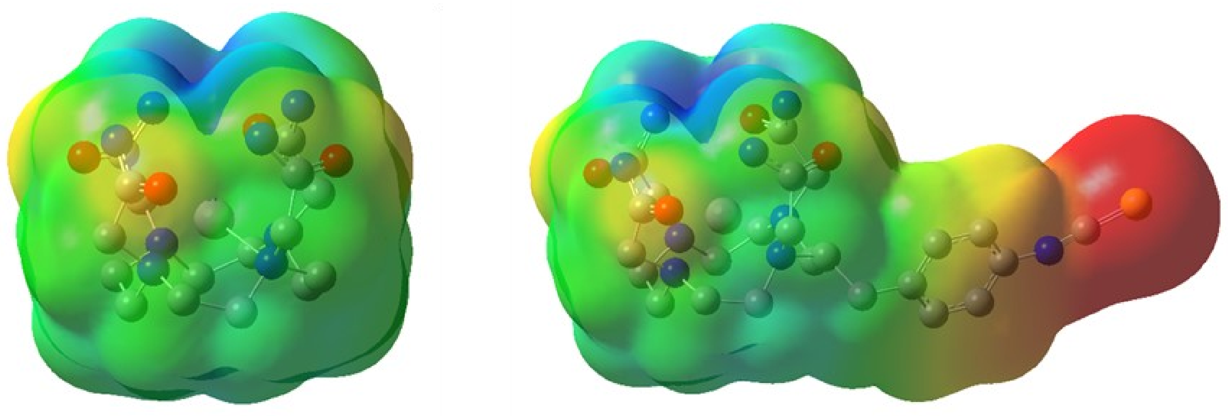

Figure S48. Molecular electrostatic potential (MEP) maps of the DFT-optimized structures of $[Hg(TCMC)]^{2+}$ (left) and $[Hg(p\text{-SCN-Bn-TCMC})]^{2+}$ (right) mapped onto the total electron density (positive MEP = blue, negative MEP = red). Minimum and maximum potentials (arbitrary units) are mapped onto electron density isosurfaces of 0.0004 $Å^{-3}$. Red indicates regions of relative negative charge and blue regions of positive charge.

References

Atkins, Peter, Tina Overton, Jonathan Rourke, Mark Weller, Fraser Armstrong, and Michael Hagerman. 2008. *Inorganic Chemistry*. Edited by Jenifer Armstrng, Sandra Dann, Russ Egdell, Jennifer Creen, Martin Jones, and Rob Deeth. *Advanced Information and Knowledge Processing*. New York: W.H. Freeman and Company. https://doi.org/10.1007/978-1-84628-986-6_2.

Cuenot, François, Michel Meyer, Enrique Espinosa, Arnaud Bucaille, Romain Burgat, Roger Guilard, and Claire Marichal-westrich. 2008. "New Insights into the Complexation of Lead (II) by 1 , 4 , 7 , 10- Structural , Thermodynamic , and Kinetic Studies," no. Ii: 267–83. https://doi.org/10.1002/ejic.200700819.

Gawronski, Jacek, Marcin Kwit, and Pawel Skowronek. 2009. "Thiourea and Isothiocyanate - Two Useful Chromophores for Stereochemical Studies. A Comparison of Experiment and Computation." *Organic and Biomolecular Chemistry* 7 (8): 1562–72. https://doi.org/10.1039/b821335f.
